# BPAGS: a web application for bacteriocin prediction via feature evaluation using alternating decision tree, genetic algorithm, and linear support vector classifier

**DOI:** 10.3389/fbinf.2023.1284705

**Published:** 2024-01-10

**Authors:** Suraiya Akhter, John H. Miller

**Affiliations:** ^1^ School of Electrical Engineering and Computer Science, Washington State University, Pullman, WA, United States; ^2^ School of Engineering and Applied Sciences, Washington State University Tri-Cities, Richland, WA, United States

**Keywords:** antimicrobial resistance, antimicrobial peptides, bacteriocin prediction, drug discovery, feature selection, machine learning, web application

## Abstract

The use of bacteriocins has emerged as a propitious strategy in the development of new drugs to combat antibiotic resistance, given their ability to kill bacteria with both broad and narrow natural spectra. Hence, a compelling requirement arises for a precise and efficient computational model that can accurately predict novel bacteriocins. Machine learning’s ability to learn patterns and features from bacteriocin sequences that are difficult to capture using sequence matching-based methods makes it a potentially superior choice for accurate prediction. A web application for predicting bacteriocin was created in this study, utilizing a machine learning approach. The feature sets employed in the application were chosen using alternating decision tree (ADTree), genetic algorithm (GA), and linear support vector classifier (linear SVC)-based feature evaluation methods. Initially, potential features were extracted from the physicochemical, structural, and sequence-profile attributes of both bacteriocin and non-bacteriocin protein sequences. We assessed the candidate features first using the Pearson correlation coefficient, followed by separate evaluations with ADTree, GA, and linear SVC to eliminate unnecessary features. Finally, we constructed random forest (RF), support vector machine (SVM), decision tree (DT), logistic regression (LR), *k*-nearest neighbors (KNN), and Gaussian naïve Bayes (GNB) models using reduced feature sets. We obtained the overall top performing model using SVM with ADTree-reduced features, achieving an accuracy of 99.11% and an AUC value of 0.9984 on the testing dataset. We also assessed the predictive capabilities of our best-performing models for each reduced feature set relative to our previously developed software solution, a sequence alignment-based tool, and a deep-learning approach. A web application, titled BPAGS (Bacteriocin Prediction based on ADTree, GA, and linear SVC), was developed to incorporate the predictive models built using ADTree, GA, and linear SVC-based feature sets. Currently, the web-based tool provides classification results with associated probability values and has options to add new samples in the training data to improve the predictive efficacy. BPAGS is freely accessible at https://shiny.tricities.wsu.edu/bacteriocin-prediction/.

## 1 Introduction

The overutilization and improper application of antibiotics have the potential to lead to the development and proliferation of antibiotic-resistant bacteria, resulting in an upsurge of infections and mortality rates. The prevailing scenario characterized by the elevation and diffusion of antibiotic-resistant bacteria poses a significant and pressing concern within the realms of public health and medicine. Annually, over 2.8 million people get infected and at least 35,000 patients die in the United States due to antibiotic resistance (Chowdhury et al., 2019a; Control CfD and Prevention, 2019). A study in 2019 examining the impact of bacterial antimicrobial resistance (AMR) for 23 pathogens and 88 pathogen-drug combinations in 204 countries and territories estimated that at least 1.27 million deaths were caused by AMR, surpassing the number of deaths from other major diseases such as malaria and HIV/AIDS (Murray et al., 2022). Traditional drugs face huge challenges due to the loss of their sensitivity to antibiotic-resistant bacteria, and it is necessary to invent novel antimicrobial compounds for the treatment of antibiotic-resistant patients (Magana et al., 2020). Bacteriocins are peptides with antimicrobial attributes that bacteria generate during ribosome synthesis in their metabolic process (Guder et al., 2000; Willey and Van Der Donk, 2007; Correia and Weimann, 2021). They exhibit potent activity against both related and unrelated bacterial strains and have become an appealing substitute for conventional antibiotics due to their broad and narrow spectrum of activity, low toxicity, and high specificity (Riley and Wertz, 2002; Hamid and Friedberg, 2017; Fields et al., 2020). Several traditional approaches such as screening assays, chromatography, and mass spectrometry are used to identify and characterize bacteriocins (Zendo et al., 2008; Zhang et al., 2018; Desiderato et al., 2021). However, detecting bacteriocins using these methods can be time-consuming, tedious, and expensive. These conventional methods also suffer from missing or underestimating the variety and originality of bacteriocins in complex microbial communities (Perez et al., 2014).

To overcome these limitations, sequence-matching computational methods such as BLASTP can be used to predict bacteriocins based on known patterns or motifs in the sequences of bacteriocins (Johnson et al., 2008; Boratyn et al., 2013). Several other online computational mining tools have been developed to help in identifying bacteriocins. BACTIBASE is an integrated open database that uses microbial information from PubMed and protein analysis tools to characterize bacteriocins (Hammami et al., 2010). BAGEL is another search tool that classifies bacteriocins sequences based on homology information (Van Heel et al., 2013). Both BACTIBASE and BAGEL maintain databases of experimentally validated bacteriocin sequences. Like BLASTP, these methods depend on sequence alignment to measure the sequence similarity between query and reference known bacteriocin sequences; hence, they have limited ability to identify novel or divergent bacteriocins that do not match these patterns due to excessive variations or mutations. Another platform, antiSMASH, utilizes hidden Markov models and BLAST searches against a known bacteriocin biosynthetic gene clusters (BGCs) database to identify and annotate putative BGCs in bacterial genomes (Medema et al., 2011; Blin et al., 2014; Weber et al., 2015). While some bacteriocin prediction tools, such as BOA have been introduced to address the issues of high diversity of bacteriocins, these platforms still depend on homology-based genome identification that can restrict their capability to identify highly dissimilar bacteriocin sequences that do not match conserved context genes of the bacteriocin operon (Morton et al., 2015).

Machine learning algorithms provide an alternative to sequence matching techniques for predicting bacteriocins, discerning patterns and characteristics within bacteriocin sequences that extend beyond their similarity to established ones. For example, we can use machine learning algorithms to analyze the physicochemical properties, sequence profiles and secondary structure of bacteriocin protein sequences to identify novel bacteriocins that may have high dissimilarity to known bacteriocins. Recently, several machine learning-based bacteriocin prediction methods were developed that use *k*-mer features and word embedding techniques. In the *k*-mer technique, features are generated from the subsequences of length *k*, whereas word embedding tactics represent peptide sequences as vectors in high dimensional space (Mikolov et al., 2013; Hamid and Friedberg, 2019). Additionally, a deep learning-based technique RMSCNN was designed to predict the presence of bacteriocins using a convolutional neural network (CNN) (Gu et al., 2018; Su et al., 2019; Cui et al., 2021). Primary and secondary structure of peptides, which play a crucial role in detecting diverse bacteriocins, were not analyzed in the existing approaches. In addition, none of those solutions employed any feature selection methods to eliminate irrelevant features that might impair the performance of a machine learning classifier. Recently, we introduced a machine learning-based software tool BaPreS (Akhter and Miller, 2023) to identify novel bacteriocins with reasonable accuracy using a support vector machine (SVM) (Cortes and Vapnik, 1995) and *a t*-test-based feature evaluation technique. Nonetheless, there is still room for enhancing prediction performance, which motivated the current study.

The objective of our work was to create a web-based application using machine learning techniques to identify bacteriocins. To achieve this, we created predictive models that leverage the physical and chemical attributes along with the sequence profiles and structural characteristics of protein sequences. We used a set of methods, including the Pearson correlation coefficient, alternating decision tree (ADTree) (Freund and Mason, 1999; Pfahringer et al., 2001), genetic algorithm (GA) (Whitley, 1994), and linear support vector classifier (linear SVC) (Pedregosa et al., 2011), to assess and select subsets of potential features for our models. Subsequently, we employed machine learning models, namely, random forest (RF), support vector machine (SVM), decision tree (DT), logistic regression (LR), *k*-nearest neighbors (KNN), and Gaussian naïve Bayes (GNB) to predict bacteriocins using the reduced feature sets, and assessed the predictive performance of these models for bacteriocin identification (Leo, 2001; Mucherino et al., 2009; Pedregosa et al., 2011; Sammut and Webb, 2011; McCullagh, 2019). Finally, we developed a web-based tool called BPAGS (Bacteriocin Prediction based on ADTree, GA, and linear SVC) where users have the freedom to choose ADTree-, GA-, or linear SVC -based selected features to obtain prediction results, and the BPAGS will automatically generate the required features of the user-supplied protein sequences. Furthermore, the web application has the option to use our previously developed BaPreS predictive tool (Akhter and Miller, 2023) to compare prediction results for the testing sequences. Users can test multiple sequences simultaneously and add new sequences to the training data to boost the predictive ability of the machine learning models. We compared the effectiveness of our web application BPAGS with the BaPreS tool, the deep learning model, RMSCNN (Cui et al., 2021), and the sequence matching tool, BLASTP (Johnson et al., 2008; Boratyn et al., 2013).

## 2 Methods

**FIGURE 1:**
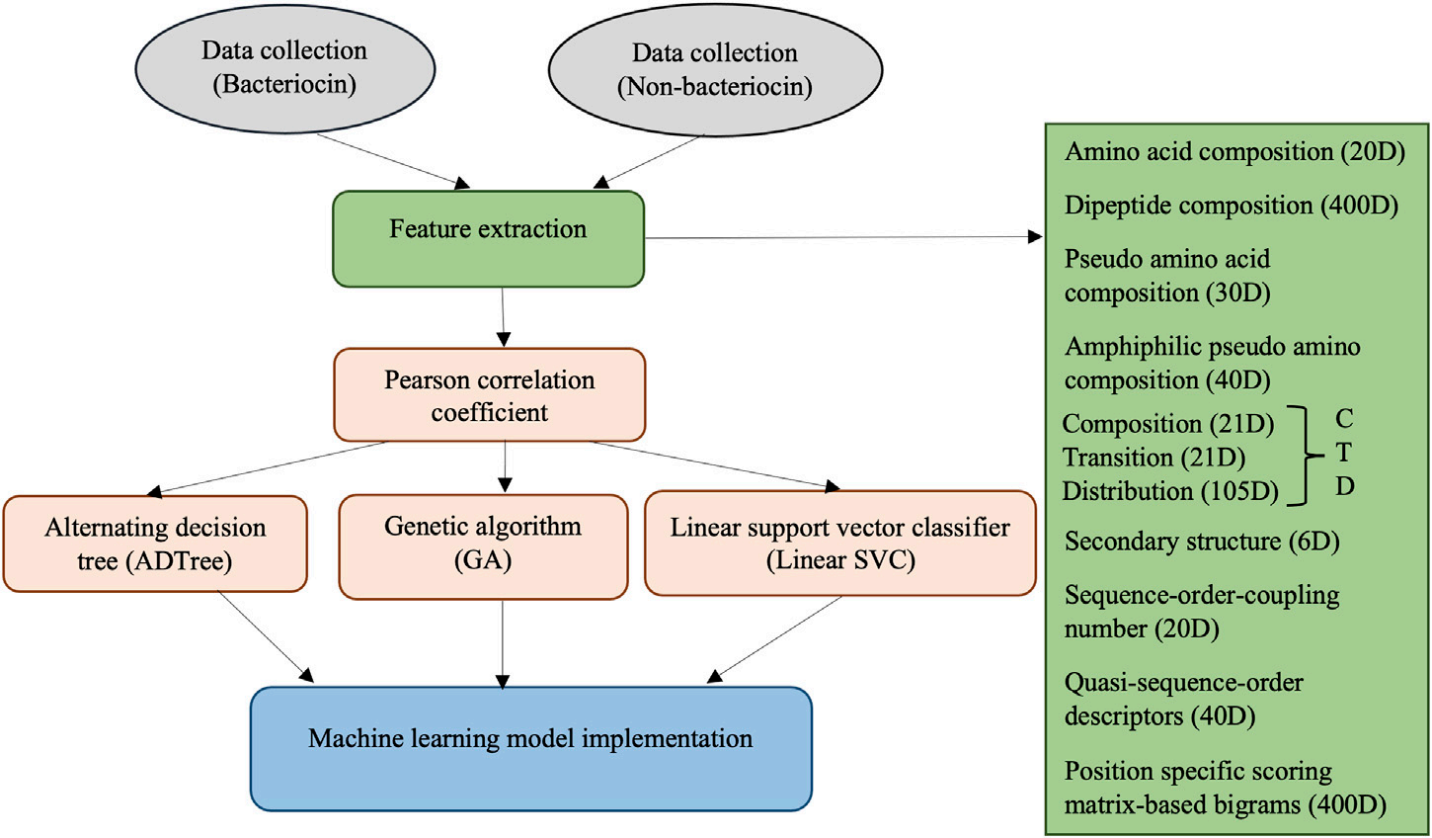
Overview of the bacteriocin prediction methods.

A depiction of the steps involved in our methodology is shown in Figure 1. The methods involve collecting bacteriocin (positive) and non-bacteriocin (negative) datasets, generating candidate features from the protein sequences, measuring the correlation among features to remove highly correlated ones, evaluating features using ADTree, GA, and linear SVC approaches to eliminate the weakest and irrelevant features, followed by constructing machine learning models using the chosen set of features. Finally, we compared the predictive efficacy of the novel models with our prior software application, sequence alignment, and deep learning techniques.

### 2.1 Data collection

The datasets used in this study were identical to those utilized in the development of our previously implemented software tool, BaPreS (Akhter and Miller, 2023). BACTIBASE (Hammami et al., 2010) and BAGEL (Van Heel et al., 2013) databases were considered to retrieve experimentally annotated and validated positive sequences. We collected negative sequences from RMSCNN (Cui et al., 2021). Initially, the dataset comprised 483 positive and 500 negative sequences. To eliminate duplicate sequences and obtain unique positive and negative sequences, we employed the CD-HIT tool (Fu et al., 2012). Sequences with a similarity greater than 90% were removed to prevent the machine learning model from being biased by duplicate or highly similar sequences. A high similarity threshold was selected due to the heterogeneity of bacteriocins, which are a diverse class of bacterial peptides (proteins) (Mesa-Pereira et al., 2018; Lertampaiporn et al., 2021). The final dataset comprises 283 unique positive sequences and 497 unique negative sequences. We performed random sampling to address the issue of imbalanced dataset by reducing the number of negative sequences from 497 to 283, thereby achieving an equal number of bacteriocin and non-bacteriocin sequences. We allocated 80% of the dataset for training purposes, while the remaining 20% was set aside for testing. The training and testing datasets are available in the Supplementary Material.

### 2.2 Feature extraction

The extraction of potential candidate features is crucial for developing a machine learning model with robust prediction capabilities. Feature vectors were formulated for the protein sequences, encompassing a 20-dimensional amino acid composition (AAC), a 400-dimensional dipeptide composition (DC), a 30-dimensional pseudo amino acid composition (PseAAC), and a 40-dimensional amphiphilic pseudo amino acid composition (APseAAC). Furthermore, we employed the composition/transition/distribution (CTD) model (Dubchak et al., 1995) to generate 147-dimensional feature vectors, considering diverse physicochemical amino acid characteristics. The detailed description of these feature vectors was discussed in our previously developed BaPreS software tool (Akhter and Miller, 2023).

We also derived 6-dimensional feature vectors for the secondary structure (SS) of individual protein sequences. This feature extraction process starts with a determination of secondary structure, resulting in a sequence characterized by three states, H (alpha-helix), E (beta-strand), and C (coil), followed by their spatial arrangements (Chowdhury et al., 2019b). Additional SS features involve analyzing consecutive E and H states in the sequence, as well as examining the frequency of EHE patterns after excluding coil segments and consolidating consecutive Hs and Es into single H and E states. We used the distance matrix between amino acids to extract sequence-order-coupling number (SOCN) feature vectors of 20D and quasi-sequence-order (QSO) feature vectors of 40D for each protein sequence (Xiao et al., 2015). Finally, we used the position-specific scoring matrix (PSSM) to extract sequence profile/evolutionary-related features from protein sequences. This process entailed generating the PSSM from protein sequences using PSI-BLAST (Altschul et al., 1997; Pande et al., 2023) and computing transition scores between adjacent amino acids to create a 400-dimensional feature vector for each sequence (Saini et al., 2016; Mohammadi et al., 2022). Table 1 lists 1,103 collected features.

**TABLE 1:** Candidate features.

Feature	Dimension
Amino acid composition	20
Dipeptide composition	400
Pseudo amino acid composition	30
Amphiphilic pseudo amino composition	40
Composition	21
Transition	21
Distribution	105
Secondary structure	6
Sequence-order-coupling number	20
Quasi-sequence-order descriptors	40
Position specific scoring matrix-based bigrams	400

### 2.3 Feature evaluation

To uphold the predictive effectiveness of a machine learning model, it is imperative to exclude unnecessary features prior to constructing the model. To avoid information leakage between training and testing datasets, we only assessed the features of the training data. Initially, we analyzed the correlation among the features. We used the Pearson correlation coefficient to determine the statistical relationship between two features, measuring the strength and direction of their linear relationship. The formula for estimating Pearson correlation coefficient $\rho_{x,y}$ between two features is given in Eq. 1.

$$\rho_{x,y}=\frac{E\left[\left(x-{µ}_{x}\right)\left(y-{µ}_{y}\right)\right]}{\sigma_{x\;}\sigma_{y}}$$ (1)

Here, two features are *x* and *y*, E stands for the expectation, ${µ}_{x}$ and ${µ}_{y}$ are mean values, and $\sigma_{x}$ and $\sigma_{y}$ indicate the standard deviation of x and y , respectively. The values of $\rho_{x,y}$ fall between −1 and +1, where 0 denotes no correlation, and −1 and +1 represent perfect negative and perfect positive correlations, respectively. In this work, we consider only the absolute value of $\rho_{x,y}.$ When two features are highly correlated (i.e., ≥ a threshold of $\left.0.9\right)$ , we can choose one of them and disregard the other. As a result, the feature count was decreased from 1,103 to 602. Supplementary Table S1 (Supplementary Material) lists the reduced feature set where “aac”, “dipep”, “pseudo”, “amphipseudo”, “comp”, “tran”, “dist”, “ss”, “qso”, and “pssm” indicate AAC, DC, PseAAC, APseAAC, composition (CTD), transition (CTD), distribution (CTD), SS, QSO, and PSSM-based features, respectively. The AAC feature *aac_i* (where *i* = 1, 2, 3, …., 20) represents amino acid composition for the *i*th amino acids in the order A, R, N, D, C, E, Q, G, H, I, L, K, M, F, P, S, T, W, Y, V, respectively. The DC feature *dipep_i* (where *i* = 1, 2, 3, …., 400) gives the dipeptide composition of the amino acids in the same order mentioned above. The PseAAC and APseAAC features are *pseudo_i* (where *i* = 1, 2, 3, …., 30) and *amphipseudo_i* (where *i* = 1, 2, 3, …., 40), respectively. The amino acid attributes are represented as *comp_i* (where *i* = 1, 2, 3, …., 21) and *tran_i* (where *i* = 1, 2, 3, …., 21) in a specific order. The first group of amino acid attributes is related to group 1, the second group is related to group 2, and so on. The distribution feature is represented as *dist_i* (where *i* = 1, 2, 3, …., 105), with the first 15 features being related to the distribution estimates of group 1, group 2, and group 3 for the first amino acid attribute, and so on. A detailed explanation of the amino acid attributes and how the amino acids are grouped into three categories is available in the *protr* R package (Xiao et al., 2015). The SS feature is represented as *ss_i* (where *i* = 1, 2, 3, …., 6). To represent the location-associated features for H, E, and C, we use the notations {*ss_1*, *ss_2*, *ss_3*} respectively. Similarly, the normalized maximum spatial consecutive E and H, and the existence of segmented sequences “EHE” are denoted as {*ss_4*, *ss_5*, *ss_6*}. The QSO descriptors and PSSM-based bigrams are represented by *qso_i* (where *i* = 1, 2, 3, …., 40) and *pssm_i* (where *i* = 1, 2, 3, …., 400), respectively.

We considered ADTree (Freund and Mason, 1999; Pfahringer et al., 2001), GA (Whitley, 1994), and linear SVC (Pedregosa et al., 2011) separately to further eliminate less important features from the feature set obtained after correlation analysis. The ADTree algorithm integrates the intuitive and easily interpretable structure of a single decision tree with the enhanced predictive performance achieved through boosting techniques. It represents knowledge using a decision tree structure that combines tree stumps, a commonly used model in boosting. A significant characteristic of this representation of a tree is that its branches are no longer limited to being exclusive of each other. The root node serves as a predictor node with a numerical score, while the subsequent layer of nodes comprises decision nodes that contain a set of decision tree stumps. Subsequent layers alternate between prediction and decision nodes. In the ADTree, decision nodes are defined by a predicate condition or criterion, while prediction nodes comprise a numerical value. It is important to note that prediction nodes always serve as both the root and leaves of an ADTree, reflecting the unique structure and functionality of this decision tree model.

The ADTree builds a set of rules, each consisting of a precondition, a condition, and two scores. A condition is expressed as a predicate in the structure of “attribute <comparison> value,” while a prerequisite is formed through a logical conjunction of conditions. Rules are evaluated with nested if statements, and the scores associated with each rule are used to determine the prediction for a given instance. The algorithm starts with a root rule, which has a precondition of “true” and a condition of “true,” and its scores are calculated based on the weighted training instances. Initially, the weights of each training instance set to $\frac{1}{t}$ , where t is the total number of training instances. The algorithm then iteratively creates new rules by finding the best combination of precondition and condition that minimizes the value of a function *z*. The value of *z* is a measure of how well a rule divides the positively and negatively labeled instances, and it is used to determine the optimal combination of precondition and condition for the new rule. In each iteration, the algorithm calculates new scores for the new rule using a boosting technique. The weights of the training instances are updated based on how well the new rule correctly predicts the label of each instance. Training persists until a halting condition is fulfilled, which could involve reaching a maximum iteration count or achieving a minimal enhancement in accuracy. The set of rules generated by the ADTree algorithm forms an alternating decision tree, where prediction nodes contain a single number, and the tree structure is determined by the preconditions used in each successive rule. The unique features that are used to construct the ADTree consist of a subset of the total features. We considered 50 randomly generated values for *B* (the number of boosting iterations) in the implementation of the ADTree. The decision tree built using the ADTree algorithm is presented in Supplementary Figure S1 (Supplementary Material), showing the selected features out of 602. We obtained 43 ADTree-reduced features, which are listed in Supplementary Table S2 (Supplementary Material).

**FIGURE 2:**
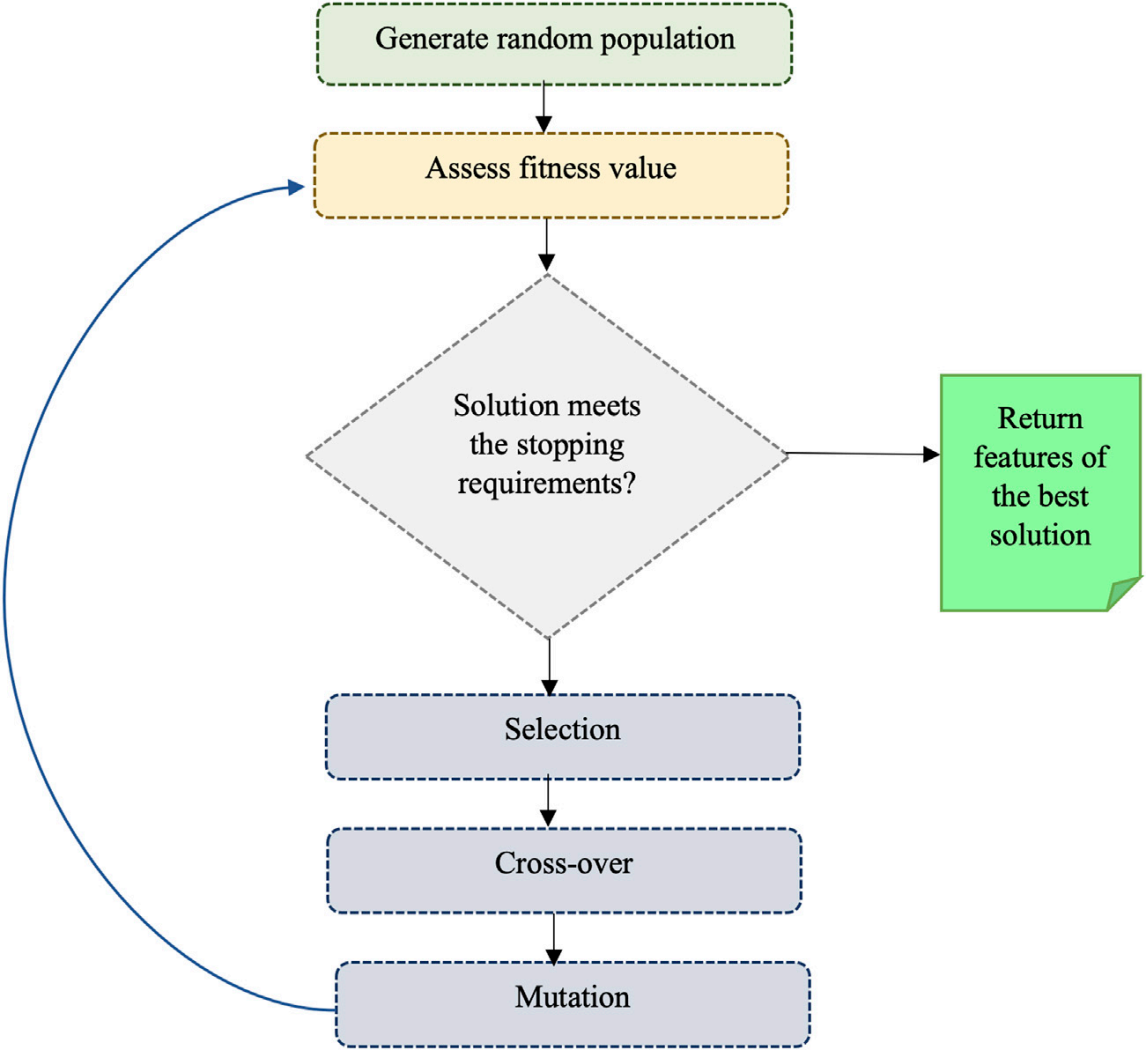
Illustrating the process of a genetic algorithm.

We also utilized the genetic algorithm (GA) which is a metaheuristic optimization algorithm that simulates the natural selection process of biological evolution, and it is commonly employed to tackle complex problems. The step of GA is given in Figure 2. To use GA for the feature selection, chromosomes (i.e., strings of bits) are generated randomly where each chromosome represents a subset of features and every bit in a string indicates whether the respective feature is present or not in the subset. A fitness function is applied to assess the quality of each chromosome, which denotes the efficiency of a specific subset of features in predicting the outcome variable. The area under the receiver operating characteristic curve (AUC-ROC) was used as the fitness function in this study. Basic GA operations such as selection (choosing the fittest chromosomes based on the highest AUC-ROC), crossover (combining the genetic information of two parent chromosomes to create a new offspring chromosome), and mutation (randomly flipping bits in the chromosome to introduce new genetic information) are performed to optimize fitness. In the GA, we used RF with 5-fold cross-validation to get AUC-ROC values for a subset of features. During the estimation of AUC-ROC, we generated square root values of the number of feature subsets to set the *mtry* parameter in RF. We set *crossover* = *gabin_uCrossover* (cross-over method), *pmutation* = 0.03 (mutation rate probability), *popSize* = 50 (the number of individuals/solutions), and *maxiter* = 50 (total runs or generations) in the genetic algorithm implementation. The algorithm terminates when a stopping criterion is met (in our case, a maximum number of generations) and provides the best subset of feature (i.e., reduced feature set). Out of 602 features, we obtained 234 GA-reduced features. The list of features obtained from GA is provided in Supplementary Table S3 (Supplementary Material).

The linear support vector classifier (linear SVC) (Pedregosa et al., 2011) offers penalty-based feature selection. This approach produces sparse solutions with numerous coefficients set to zero, enabling efficient identification and selection of non-zero coefficients. The value of *C* (regularization parameter) in linear SVC plays a pivotal role in controlling sparsity, allowing us to fine-tune the number of selected features. We chose *C* = 0.01 and a penalty of *l1* for feature selection. We selected 39 features out of 602 using this method, and they are listed in Supplementary Table S4 (Supplementary Material).

### 2.4 BPAGS web application

**FIGURE 3:**
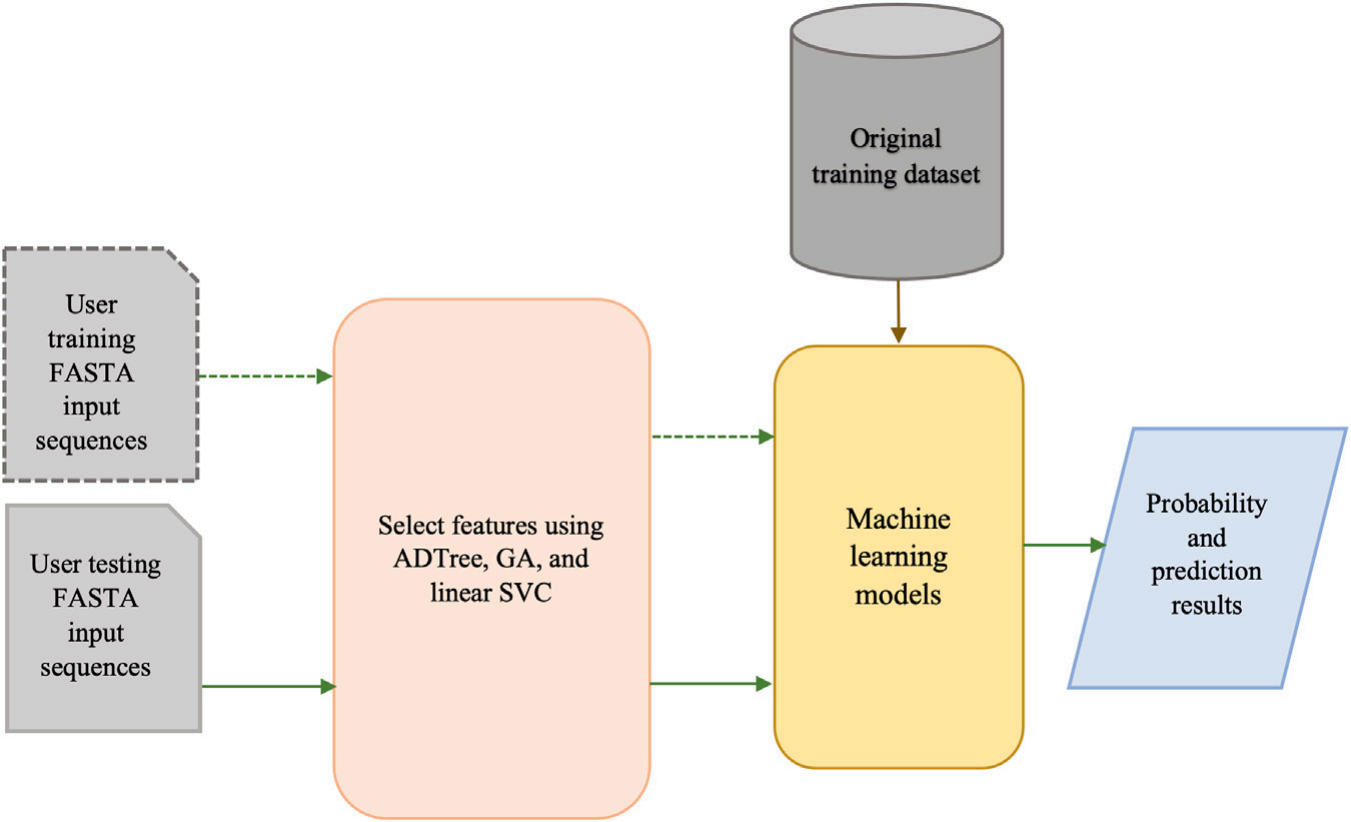
Architecture of the BPAGS web application.

The architecture of our BPAGS web application is depicted in Figure 3. Our machine learning-based web application can automatically generate all required features for user-supplied training and testing sequences. After machine-learning models are generated using the training dataset, classification and probability results are provided for the testing dataset.

**FIGURE 4:**
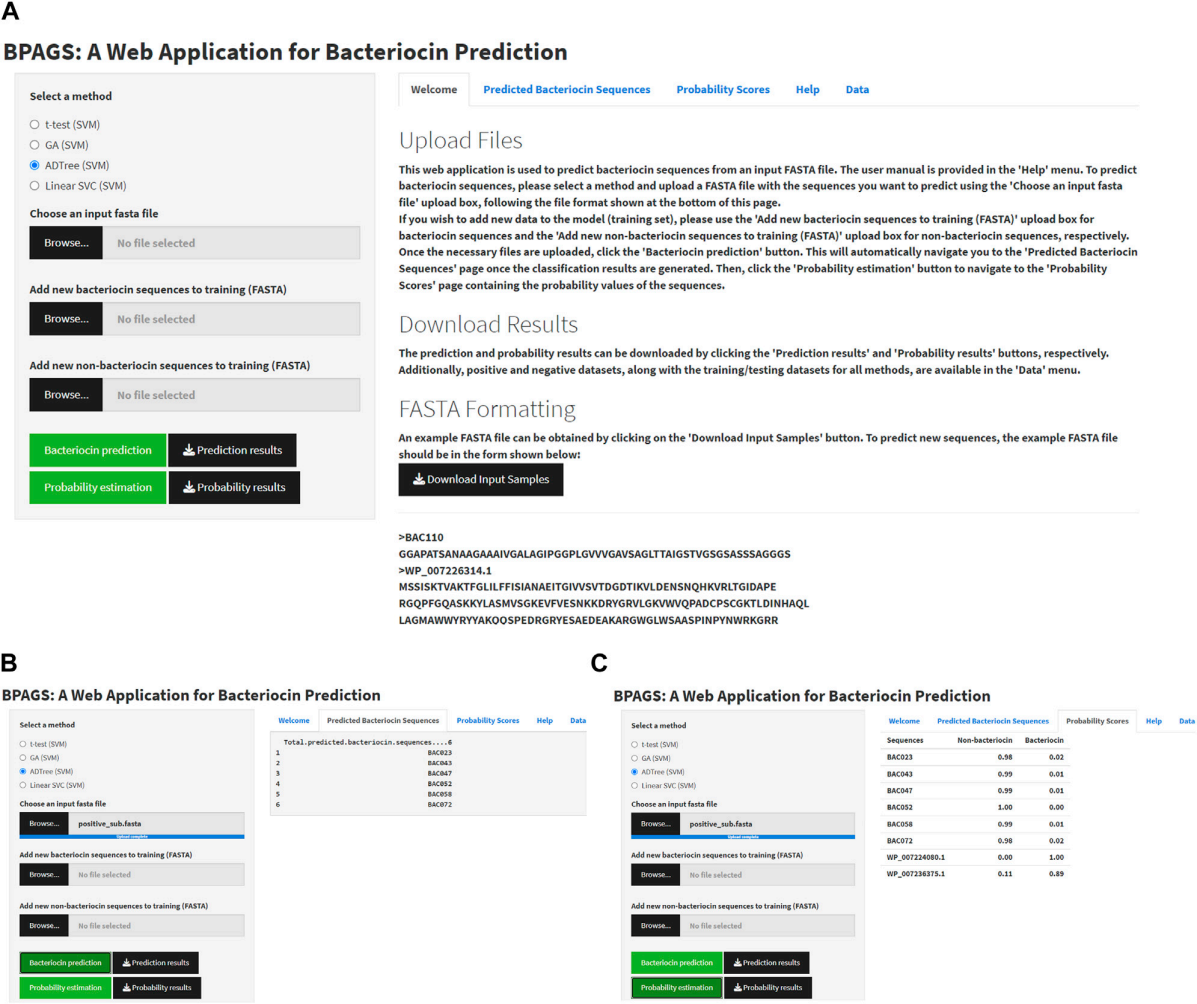
Illustrating the interface of the BPAGS web application. **(A)** depicts the GUI; **(B)** shows sample prediction results; **(C)** shows sample probability results.

Figure 4 shows the graphical user interface (GUI), service menu, and sample outputs of the web tool BPAGS. Features required for the web application are generated in R. R Shiny was utilized to design the GUI. In this web application, users can upload input files containing protein sequences in FASTA format. In addition to machine learning models based on ADTree-reduced features, GA-reduced features, and linear SVC-reduced features, BPAG provides an additional option for testing sequences using our previously developed BaPreS software tool (Akhter and Miller, 2023). After selecting the appropriate feature selection method and uploading an input FASTA file with the sequences for prediction, the user should click on the ‘Bacteriocin prediction’ button first to view the binary classification results for the testing sequences in their input. Subsequently, to obtain the predicted probability values for the testing sequences, the user can click on the “Probability estimation” button. Buttons are provided in the web application to download and save these results. The web tool includes positive and negative datasets, along with the current training/testing files for all methods, which are available for users to download. Furthermore, the web application allows users to augment the training dataset with new protein sequences, whether bacteriocin or non-bacteriocin, to enhance prediction accuracy. More specifically, users have the option to collect and upload new sequences for retraining the model and obtaining prediction results for test sequences. Additionally, the webserver provides a user manual for download. Our BPAGS web application is publicly available for all users and can be found at https://shiny.tricities.wsu.edu/bacteriocin-prediction/.

## 3 Results

We considered several machine learning algorithms to build predictive models with the selected feature sets. We assessed the efficacy of these models and contrasted the optimal model with our previously introduced machine learning-oriented software tool, as well as two other preexisting tools based on deep learning and sequence matching.

### 3.1 Prediction performance

We trained RF, SVM, DT, LR, KNN, and GNB machine learning models using the reduced feature sets obtained from ADTree, GA, and Linear SVC methods. During training, we tuned these algorithms to find the optimal models by identifying the most suitable parameters. The list of parameters to tune the models, and their best values are provided in Supplementary Table S5 (Supplementary Material). We assessed the predictive capability using Eqs 2–6. TP denotes instances where positive outcomes were predicted correctly, TN represents instances where negative outcomes were predicted correctly, FP signifies cases where there was an incorrect prediction of positive outcomes, while FN represents cases where there was an incorrect prediction of negative outcomes. Matthews correlation coefficient (MCC) was also computed to evaluate the effectiveness of our predictive models. This metric, which is particularly useful when dealing with imbalanced datasets, ranges between −1 and +1, with a score of +1 indicating a flawless prediction, 0 indicating a random prediction, and −1 indicating complete divergence between the prediction and ground-truth observation. To further evaluate the performance of our models, we computed recall, which measures the fraction of true positive instances that were correctly identified, and precision, which measures the fraction of positive predictions that are true. The F1 score is a metric that considers both precision and recall by calculating their harmonic mean, thereby providing a balanced measure of a model’s performance.

$$Test_{acc}=\frac{TP+TN}{TP+TN+FP+FN}$$ (2)

$$Test_{MCC}=\frac{TP\times TN-FP\times FN}{\sqrt{\left(TP+FP\right)\left(TP+FN\right)\left(TN+FP\right)\left(TN+FN\right)}}$$ (3)

$$Test_{recall}=\frac{TP}{TP+FN}$$ (4)

$$Test_{precision}=\frac{TP}{TP+FP}$$ (5)

$${Test}_{F1}=2\times \frac{\left({Test}_{precision}\times {Test}_{recall}\right)}{\left({Test}_{precision}+{Test}_{recall}\right)}$$ (6)

Confidence intervals are also estimated to provide the range of values in which the true performance metrics of the models are likely to fall, based on a 95% level of confidence, which means if the same experiment were repeated many times, the true performance metric would expect to lie within this interval in 95% of the experiments. A wider confidence interval indicates higher uncertainty of the prediction s. The area under the curve (AUC) $Test_{AUC}$ were calculated to measure the performance of binary classification models. AUC measures the overall discriminative power of a classification model. Higher AUC values indicate better performance, with 1 being perfect and 0.5 random chance.

**FIGURE 5:**
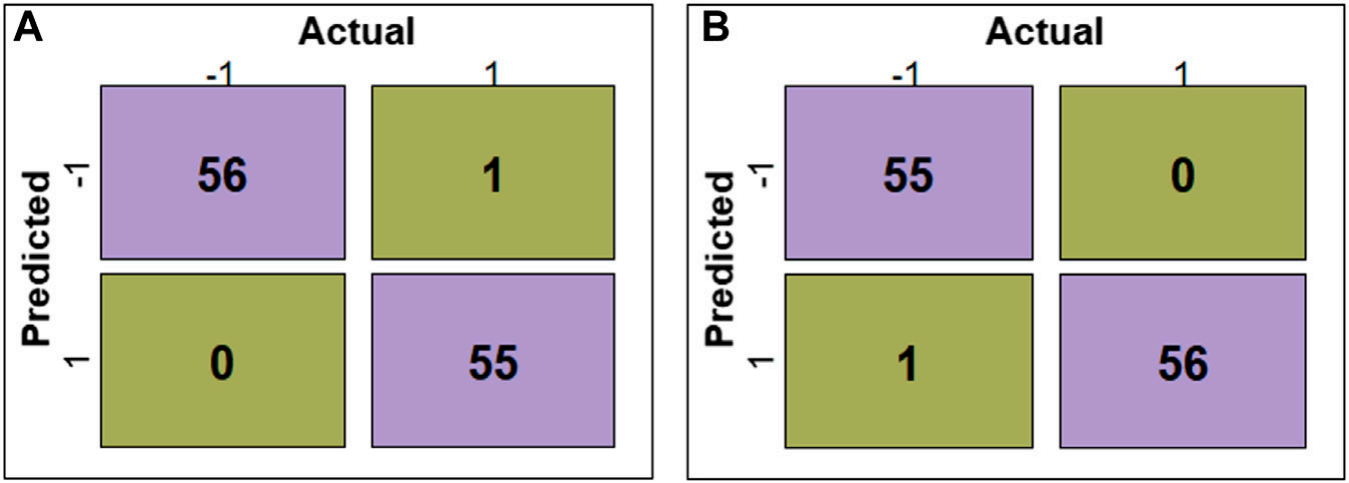
Confusion matrices depicting the model performance. **(A)** shows confusion matrix for the RF model constructed using the ADTree-reduced feature set, and **(B)** shows confusion matrix for the SVM model constructed using the ADTree-reduced feature set.

The evaluation of RF, SVM, DT, LR, KNN, and GNB models’ performance across different feature subsets is outlined in Table 2. The confusion matrices for RF and SVM models constructed using ADTree-reduced feature sets are provided in Figure 5, while those for all other models can be found in Supplementary Figure S2 (Supplementary Material). Figure 5 shows that RF and SVM models, based on the ADTree-reduced feature set, successfully identified 55 and 56 protein sequences as bacteriocins, respectively. These findings indicate that with regard to ADTree-reduced feature sets, the SVM model demonstrates better overall performance compared to the other models in terms of the number of predicted bacteriocin sequences, prediction accuracy, and AUC. We obtained the best models for ADTree-, GA-, and linear SVC-reduced feature sets using SVM, and the probability scores for all test sequences generated by these models are available in Supplementary Table S6 (Supplementary Material).

**TABLE 2:** Evaluation of RF, SVM, DT, LR, KNN, and GNB model performance with varied feature sets.

Feature set	Machine learning models	$Test_{acc}$	$Test_{MCC}$	Confidence interval (95%)	$Test_{precision}$	$Test_{recall}$	${Test}_{F1}$	${Test}_{AUC}$
ADTree	RF	0.9911	0.9823	(0.9513,0.9998)	1.0000	0.9821	0.9910	0.9965
	SVM	0.9911	0.9823	(0.9513,0.9998)	0.9825	1.0000	0.9912	0.9984
	DT	0.9464	0.8934	(0.887, 0.9801)	0.9630	0.9286	0.9455	0.9381
	LR	0.9821	0.9649	(0.937, 0.9978)	1.0000	0.9643	0.9818	0.9987
	KNN	0.8839	0.7894	(0.8097,0.9367)	1.0000	0.7679	0.8687	0.9601
	GNB	0.9286	0.8577	(0.8641,0.9687)	0.9444	0.9107	0.9273	0.9665
GA	RF	0.9643	0.9309	(0.9111,0.9902)	1.0000	0.9286	0.9630	0.9906
	SVM	0.9643	0.9286	(0.9111,0.9902)	0.9643	0.9643	0.9643	0.9968
	DT	0.9554	0.9109	(0.8989,0.9853)	0.9636	0.9464	0.9550	0.9633
	LR	0.9643	0.9286	(0.9111,0.9902)	0.9643	0.9643	0.9643	0.9901
	KNN	0.7946	0.6264	(0.708, 0.8651)	0.9459	0.6250	0.7527	0.8653
	GNB	0.9375	0.8763	(0.8755,0.9745)	0.9623	0.9107	0.9358	0.9350
Linear SVC	RF	0.9732	0.9478	(0.9237,0.9944)	1.0000	0.9464	0.9725	0.9904
	SVM	0.9732	0.9466	(0.937, 0.9978)	0.9818	0.9643	0.9730	0.9990
	DT	0.9375	0.8751	(0.8755,0.9745)	0.9298	0.9464	0.9381	0.9531
	LR	0.9643	0.9292	(0.9111,0.9902)	0.9815	0.9464	0.9636	0.9908
	KNN	0.9464	0.8951	(0.887,0.9801)	0.9808	0.9107	0.9444	0.9955
	GNB	0.9286	0.8577	(0.8641,0.9687)	0.9444	0.9107	0.9273	0.9601

Legend: $Test_{acc}$ , Accuracy on the testing dataset; $Test_{MCC}$ , MCC on the testing dataset; $Test_{precision}$ , Precision on the testing dataset; $Test_{recall}$ , Recall on the testing dataset; ${Test}_{F1}$ , F1 score on the testing dataset; $Test_{AUC}$ , AUC on the testing dataset.

### 3.2 Selected features in the alternating decision tree

Our best machine-learning results were obtained using SVM with the ADTree-reduced feature set. As mentioned earlier, 43 features out of 602 were selected by the constructed ADTree. In ADTree, most of the features selected are based on dipeptide composition. Refer to Supplementary Table S2 (Supplementary Material) for the selected features.

### 3.3 Performance comparison

We developed the BPAGS web application by employing machine learning models using the chosen feature set and compared its performance to a deep learning method RMSCNN (Cui et al., 2021), our previously released machine learning-based tool BaPreS (Akhter and Miller, 2023), and sequence matching tool BLASTP (Johnson et al., 2008; Boratyn et al., 2013). RMSCNN (https://github.com/cuizhensdws/RWMSCNN) was developed for identifying marine microbial bacteriocins using CNN. The protein sequences are encoded into numerical representations to be fed into the CNN for feature learning and prediction. The BaPreS tool automatically generates the required features obtained after correlation and *t*-test analyses and utilizes an SVM with the selected features for prediction. Note that our web application BPAGS gives users an option to use the BaPreS tool for testing sequences. The sequence alignment tool BLASTP (https://blast.ncbi.nlm.nih.gov/Blast.cgi?PAGE=Proteins) is used to match a query protein sequence against a protein sequence database, aiming to identify homologous sequences. It estimates percent identity based on the alignments between the sequences, and the higher the percent identity, the higher the similarity among the sequences.

The prediction metrics of all methods/tools are shown in Table 3. Our web application BPAGS outperforms both RMSCNN and BaPreS using the same training and testing datasets. In our previous study, we showed the superiority of BaPreS over BLASTP in detecting bacteriocins (Akhter and Miller, 2023). Given that BPAGS has demonstrated superior prediction results compared to BaPreS, we can infer that BPAGS has a stronger predictive ability than BLASTP. To correctly identify equivalent true positives and true negatives aligning with the performance of our web application, BLASTP demands identity thresholds of less than 30% and 20%, respectively. Large numbers of false positives and false negatives are expected when BLASTP is used with such low identity thresholds.

**TABLE 3:** Performance assessment of the machine learning and deep learning approaches in predicting bacteriocins.

Method/tool	$Test_{acc}$	$Test_{precision}$	$Test_{recall}$	${Test}_{F1}$	${Test}_{AUC}$
RMSCNN	0.9375	0.9623	0.9107	0.9358	0.9818
BaPreS	0.9554	0.9636	0.9464	0.9550	0.9879
BPAGS (ADTree)	0.9911	0.9825	1.0000	0.9912	0.9984
BPAGS (GA)	0.9643	0.9643	0.9643	0.9643	0.9968
BPAGS (Linear SVC)	0.9732	0.9818	0.9643	0.9730	0.9990

Legend: $Test_{acc}$ , Accuracy on the testing dataset; $Test_{precision}$ , Precision on the testing dataset; $Test_{recall}$ , Recall on the testing dataset; ${Test}_{F1}$ , F1 score on the testing dataset; $Test_{AUC}$ , AUC on the testing dataset.

## 4 Discussion

Accurately predicting bacteriocins is essential for discovering new antimicrobial peptides and designing novel peptides with enhanced bioactivity and stability to combat antibiotic resistance. Machine learning models that incorporate multiple potential features have demonstrated high accuracy in predicting bacteriocins. Here, we presented a bacteriocin prediction pipeline based on machine learning that utilized physicochemical, structural, and sequence profile features derived from protein sequences. The feature selection process involved utilizing the Pearson correlation coefficient and implementing ADTree, GA, and linear SVC to reduce the feature set. Subsequently, we used several machine-learning algorithms, including RF, SVM, DT, LR, KNN, and GNB to construct predictive models using the reduced feature sets. Overall, the SVM model with the ADTree-reduced feature set is identified as the top-performing model in terms of a higher number of true positive bacteriocin sequences, prediction accuracy, and AUC. This suggests that it utilized the most important features derived from protein sequences effectively. We developed a standalone web application BPAGS based on different reduced feature sets so that users can test their protein sequences for bacteriocin prediction without having any programming knowledge. Additionally, users can include their training sequences to further enhance the prediction strength of the models. In addition to the three feature selection approaches discussed in this work, the web application also allows the use of our previously developed tool, BaPreS, to compare prediction results for the testing dataset.

Most of the selected features found in the ADTree are from dipeptide features. Dipeptide composition is an important feature for bacteriocin prediction as it provides insights into the amino acid composition and arrangement within a bacteriocin peptide (Gabere and Noble, 2017). As many bacteriocins are cationic molecules and bear hydrophobic or amphiphilic characteristics, dipeptides (i.e., pairs of adjacent amino acids) can be indicative of hydrophobicity and secondary structure (Darbandi et al., 2022; Akhter and Miller, 2023). In addition to dipeptides, ADTree identified features such as amino acid composition and secondary structure which are also crucial in achieving high prediction accuracy. Hence, we conclude that the ADTree was able to select the most important and limited number of features (i.e., only 43 features) that helps achieve strong prediction results from the SVM machine learning model. Our models showed better prediction results than sequence matching, deep learning, and our previously introduced methods. Therefore, we deduce that our web-based application BPAGS effectively employed pivotal features to accurately discern markedly distinct bacteriocins with a satisfactory level of accuracy. Researchers can use our web application without having any programming knowledge to detect bacteriocins from various sources including bacteria, the human body, the environment, and animal and plant-associated niches. Also, the data and script used in this work are publicly accessible at https://github.com/suraiya14/BPAGS, facilitating their reuse in similar biological applications.

This study has several limitations. The development of the BPAGS was based on a restricted set of distinct bacteriocin and non-bacteriocin sequences. Therefore, the outcomes of our assessments could potentially be influenced by unknown biases. Though we applied the cross-validation method to generalize our predictive models, we will add more experimentally validated nonduplicate bacteriocin and non-bacteriocin sequences in our web application whenever they are available. Currently, the BPAGS web application does not include any visual representations that demonstrate how the features contribute to the prediction results. To address this, we intend to incorporate SHAP (Shapley Additive Explanations) methodology (Lundberg and Lee, 2017; Lundberg et al., 2020) to quantitatively measure the incremental influence of individual features on the predictions made by the machine learning models. In the future, we plan to integrate more features from protein-protein interactions, metabolomics, and gene expression information to improve the robustness of our web application. Upgrading the web application by integrating a feature stacking or ensemble technique may assist in detecting novel bacteriocin more precisely. Also, we will incorporate more feature selection methods (Li et al., 2017) so that users can compare the prediction results for each test sequence and determine bacteriocins more confidently.

## Data Availability

The datasets used in this study are included in the Supplementary Material. Additionally, all scripts written for the methods and performance evaluations of the models can be found at https://github.com/suraiya14/BPAGS. Further inquiries can be directed to the corresponding author.
