# Supplementary material for: BPAGS: a web application for bacteriocin prediction via feature evaluation using alternating decision tree, genetic algorithm, and linear support vector classifier
Source: Front Bioinform. 2024 Jan 10;3:1284705. doi: 10.3389/fbinf.2023.1284705 (PMC10807691; doi:10.3389/fbinf.2023.1284705)
Supplement: Supplementary file 1 [file DataSheet1.docx]

Supplementary Material

BPAGS: A Web Application for Bacteriocin Prediction via Feature Evaluation Using Alternating Decision Tree, Genetic Algorithm, and Linear Support Vector Classifier

Suraiya Akhter^*^, John H. Miller

**^*^Correspondence:** suraiya.akhter@wsu.edu

# Supplementary Data

## Training dataset

**Bacteriocin**

**----------------------------------------**

>BAC010

CRQSCSFGPFTFVCDGNTK

>BAC011

CANSCSYGPLTWSCDGNTK

>BAC014

CTFTLPGGGGVCTLTSECIC

>BAC015

GGAGHVPEYFVGIGTPISFYG

>BAC017

IASKFICTPGCAKTGSFNSYCC

>BAC023

GNGVLKTISHECNMNTWQFLFTCC

>BAC024

GNPKVAHCASQIGRSTAWGAVSGA

>BAC025

NRWWQGVVPTVSYECRMNSWQHVFTCC

>BAC030

GKNGVFKTISHECHLNTWAFLATCCS

>BAC033

KGGSGVIHTISHEVIYNSWNFVFTCCS

>BAC034

KGGSGVIHTISHECNMNSWQFVFTCCS

>BAC042

KGKGFWSWASKATSWLTGPQQPGSPLLKKHR

>BAC043

SASVLKTSIKVSKKYCKGVTLTCGCNITGGK

>BAC045

WKSESLCTPGCVTGALQTCFLQTLTCNCKISK

>BAC047

ITSISLCTPGCKTGALMGCNMKTATCHCSIHVSK

>BAC048

TAGPAIRASVKQCQKTLKATRLFTVSCKGKNGCK

>BAC055

SDCNINSNTAADVILCFNQVGSCALCSPTLVGGPVP

>BAC056

KYYGNGLSCSKKGCTVNWGQAFSCGVNRVATAGHGK

>BAC057

STPVLASVAVSMELLPTASVLYSDVAGCFKYSAKHHC

>BAC058

KYYGNGVHCTKSGCSVNWGEAFSAGVHRLANGGNGFW

>BAC060

TSYGNGVHCNKSKCWIDVSELETYKAGTVSNPKDILW

>BAC062

GLGKAQCAALWLQCASGGTIGCGGGAVACQNYRQFCR

>BAC065

ARSYGNGVYCNNKKCWVNRGEATQSIIGGMISGWASGLAGM

>BAC069

AISYGNGVYCNKEKCWVNKAENKQAITGIVIGGWASSLAGMGH

>BAC070

TKYYGNGVYCNSKKCWVDWGTAQGCIDVVIGQLGGGIPGKGKC

>BAC071

KNYGNGVHCTKKGCSVDWGYAWTNIANNSVMNGLTGGNAGWHN

>BAC072

VGIGGGGGGGGGGSCGGQGGGCGGCSNGCSGGNGGSGGSGSHI

>BAC074

QINWGSVVGHCIGGAIIGGAFSGGAAAGVGCLVGSGKAIINGL

>BAC080

MGAIAKLVAKFGWPIVKKYYKQIMQFIGEGWAINKIIDWIKKHI

>BAC081

KYYGNGVSCNKNGCTVDWSKAIGIIGNNAAANLTTGGAAGWNKG

>BAC085

ANCSCSTASDYCPILTFCTTGTACSYTPTGCGTGWVYCACNGNFY

>BAC087

SLQYVMSAGPYTWYKDTRTGKTICKQTIDTASYTFGVMAEGWGKTFH

>BAC089

MDKFEKISTSNLEKISGGDLTTKLWSSWGYYLGKKARWNLKHPYVQF

>BAC090

ILFSYLLFYVLKENSKREDKYQNIIEELTELLPKIKEDVEDIKEKLNK

>BAC091

VNYGNGVSCSKTKCSVNWGQAFQERYTAGINSFVSGVASGAGSIGRRP

>BAC095

NRWTNAYSAALGCAVPGVKYGKKLGGVWGAVIGGVGGAAVCGLAGYVRKG

>BAC097

MQKPEIISADLGLCAVNEFVALAAIPGGAATFAVCQMPNLDEIVSNAAYV

>BAC098

MKLPVQQVYSVYGGKDLPKGHSHSTMPFLSKLQFLTKIYLLDIHTQPFFI

>BAC100

DQMSDGVNYGKGSSLSKGGAKCGLGIVGGLATIPSGPLGWLAGAAGVINSCMK

>BAC102

KLTFIQSTAAGDLYYNTNTHKYVYQQTQNAFGAAANTIVNGWMGGAAGGFGLHH

>BAC103

MNNLNKFSTLGKSSLSQIEGGSVPTSVYTLGIKILWSAYKHRKTIEKSFNKGFYH

>BAC118

ATYYGNGVYCNKQKCWVDWSRARSEIIDRGVKAYVNGFTKVLGGIGGR

>BAC119

GETDPNTQLLNDLGNNMAWGAALGAPGGLGSAALGAAGGALQTVGQGLIDHGPVNVPIPV

LIGPSWNGSGSGYNSATSSSGSGS

>BAC122

ASIIKTTIKVSKAVCKTLTCICTGSCSNCK

>BAC123

VPGGCTYTRSNRDVIGTCKTGSGQFRIRLDCNNAPDKTSVWAKPKVMVSVHCLVGQPRSI

SFETK

>BAC124

KYYGNGVHCGKKTCYVDWGQATASIGKIIVNGWTQHGPWAHR

>BAC125

DIAPPGPNGDPKSVQIDDKYTGAEMYGEGDFRVGLGTDLTMYPPVYRESLGNGSGGWEFD

FTVCGSTACRFVDSNGDVKEDDKAKEMWWQEINFNDINQDLYSRNDSDWVGSTPADTQPE

FDYTDFALARDGVTLALTALNPAMGSLALGATYFLSDMVNWIASQHEDDSSLKRKWDYDG

LSGPLYADSSTYLLARDEMTSNSYESFTIDNIAVAFPEFPVRTKYYVTFTAPDDPSTQSI

STLEEEGIYRVPATE

>BAC126

MARPIADLIHFNSTTVTASGDVYYGPGGGTGIGPIARPIEHGLDSSTENGWQEFESYADV

GVDPRRYVPLQVKEKRREIELQFRDAEKKLEASVQAELDKADAALGPAKNLAPLDVINRS

LTIVGNALQQKNQKLLLNQKKITSLGAKNFLTRTAEEIGEQAVREGNINGPEAYMRFLDR

EMEGLTAAYNVKLFTEAISSLQIRMNTLTAAKASIEAAAANKAREQAAAEAKRKAEEQAR

QQAAIRAANTYAMPANGSVVATAAGRGLIQVAQGAASLAQAISDAIAVLGRVLASAPSVM

AVGFASLTYSSRTAEQWQDQTPDSVRYALGMDAAKLGLPPSVNLNAVAKASGTVDLPMRL

TNEARGNTTTLSVVSTDGVSVPKAVPVRMAAYNATTGLYEVTVPSTTAEAPPLILTWTPA

SPPGNQNPSSTTPVVPKPVPVYEGATLTPVKATPETYPGVITLPEDLIIGFPADSGIKPI

YVMFRDPRDVPGAATGKGQPVSGNWLGAASQGEGAPIPSQIADKLRGKTFKNWRDFREQF

WIAVANDPELSKQFNPGSLAVMRDGGAPYVRESEQAGGRIKIEIHHKVRVADGGGVYNMG

NLVAVTPKRHIEIHKGGK

>BAC128

GWWNSWGKCVAGTIGGAGTGGLGGAAAGSAVPVIGTGIGGAIGGVSGGLTGAATFC

>BAC131

MDKVTDNSPDVESTESTEGSFPTVGVDTGDTITATLATGTENVGGGGGAFGGASESSAAI

HATAKWSTAQLKKHQAEQAARAAAAEAALAKAKSQRDALTQRLKDIVNDALRANAARSPS

VTDLAHANNMAMQAEAERLRLAKAEQKAREEAEAAEKALREAERQRDEIARQQAETAHLL

AMAEAAEAEKNRQDSLDEEHRAVEVAEKKLAEAKAELAKAESDVQSKQAIVSRVAGELEN

AQKSVDVKVTGFPGW

>BAC132

MGSNGADNAHNNAFGGGKNPGIGNTSGAGSNGSASSNRGNSNGWSWSNKPHKNDGFHSDG

SYHITFHGDNNSKPKPGGNSGNRGNNGDGASAKVGEITITPDNSKPGRYISSNPEYSLLA

KLIDAESIKGTEVYTFHTRKGQYVKVTVPDSNIDKMRVDYVNWKGPKYNNKLVKRFVSQF

LLFRKEEKEKNEKEALLKASELVSGMGDKLGEYLGVKYKNVAKEVANDIKNFHGRNIRSY

NEAMASLNKVLANPK

>BAC134

MKHLNETTNVRILSQFDMDTGYQAVVQKGNVGSKYVYGLQLRKGATTILRGYRGSKINNP

ILELSGQAGGHTQTWEFAGDRKDINGEERAGQWFIGVKPSKIEGSKIIWAKQIARVDLRN

QMGPHYSNTDFPRLSYLNRAGSNPFAGNKMTHAEAAVSPDYTKFLIATVENNCIGHFTIY

NLDTINEKLDEKGNSEDVNLETVKYEDSFIIDNLYGDDNNSIVNSIQGYDLDNDGNIYIS

SQKAPDFDGSYYAHH

>BAC135

METLTVHAPSPSTNLPSYGNGAFSLSAPHVPGAGPLLVQVVYSFFQSPNMCLQALTQLED

YIKKHGASNPLTLQIISTNIGYFCNADRNLVLHPGISVYDAYHFAKPAPSQYDYRSMNMK

QMSGNVTTPIVALAHYLWGNGAERSVNIANIGLKISPMKINQIKDIIKSGVVGTFPVSTK

FTHATGDYNVITGAYLGNITLKTEGTLTISANGSWTYNGVVRSYDDKYDFNASTHRGIIG

ESLTRLGAMFSGKEY

>BAC136

MNKTHKMATLVIAAILAAGMTAPTAYADSPGNTRITASEQSVLTQILGHKPTQTEYNRYV

ETYGSVPTEADINAYIEASESEGSSSQTAAHDDSTSPGTSTEIYTQAAPARFSMFFLSGT

WITRSGVVSLSLKPRKGGIGNEGDERTWKTVYDKFHNAGQWTRYKNNGVDASMKKQYMCH

FKYGMVKTPWNLEPHKKAADVSPVKCN

>BAC141

MSWLNFLKYIAKYGKKAVSAAWKYKGKVLEWLNVGPTLEWVWQKLKKIAGL

>BAC142

ATYYGNGLYCNKEKCWVDWNQAKGEIGKIIVNGWVNHGPWAPRR

>BAC143

MAKEFGIPAAVAGTVLNVVEAGGWVTTIVSILTAVGSGGLSLLAAAGRESIKAYLKKEIK

KKGKRAVIAW

>BAC148

LVAYGIAQGTAEKVVSLINAGLTVGSIISILGGVTVGLSGVFTAVKAAIAKQGIKKAIQL

>BAC150

KTVNYGNGLYCNQKKCWVNWSETATTIVNNSIMNGLTGGNAGWHSGGRA

>BAC153

TTKNYGNGVCNSVNWCQCGNVWASCNLATGCAAWLCKLA

>BAC154

DIGGSRQGCVA

>BAC157

MMNATENQIFVETVSDQELEMLIGGAGRGWIKTLTKDCPNVISSICAGTIITACKNCA

>BAC160

MQTIKELNTMELQEIIGGENDHRMPYELNRPNNLSKGGAKCAAGILGAGLGAVGGGPGGF

ISAGISAVLGCM

>BAC161

AYPGNGVHCGKYSCTVDKQTAIGNIGNNAA

>BAC162

LAGYTGIASGTAKKVVDAIDKGAAAFVIISIISTVISAGALGAVSASADFIILTVKNYIS

RNLKAQAVIW

>BAC164

MSLLALVAGTLGVSQSIATTVVSIVLTGSTLISIILGITAILSGGVDAILEIGWSAFVAT

VKKIVAERGKAAAIAW

>BAC166

WFYQGMNIAIYANIGGVANIIGYTEAAVATLLGAVVAVAPVVP

>BAC170

MAGFLKVVQILAKYGSKAVQWAWANKGKILDWINAGQAIDWVVEKIKQILGIK

>BAC172

MSDPVRITNPGAESLGYDSDGHEIMAVDIYVNPPRVDVFHGTPPAWSSFGNKTIWGGNEW

VDDSPTRSDIEKRDKEITAYKNTLSAQQKENENKRTEAGKRLSAAIAAREKDENTLKTLR

AGNADAADITRQEFRLLQAELREYGFRTEIAGYDALRLHTESRMLFADADSLRISPREAR

SLIEQAEKRQKDAQNADKKAADMLAEYERRKGILDTRLSELEKNGGAALAVLDAQQARLL

GQQTRNDRAISEARNKLSSVTESLNTARNALTRAEQQLTQQKNTPDGKTIVSPEKFPGRS

STNHSIVVSGDPRFAGTIKITTSAVIDNRANLNYLLSHSGLDYKRNILNDRNPVVTEDVE

GDKKIYNAEVAEWDKLRQRLLDARNKITSAESAVNSARNNLSARTNEQKHANDALNALLK

EKENIRNQLSGINQKIAEEKRKQDELKATKDAINFTTEFLKSVSEKYGAKAEQLAREMAG

QAKGKKIRNVEEALKTYEKYRADINKKINAKDRAAIAAALESVKLSDISSNLNRFSRGLG

YAGKFTSLADWITEFGKAVRTENWRPLFVKTETIIAGNAATALVALVFSILTGSALGIIG

YGLLMAVTGALIDESLVEKANKFWGI

>BAC173

MSDPVRITNPGAESLGYDSDGHEIMAVDIYVNPPRVDVFHGTPPAWSSFGNKTIWGGNEW

VDDSPTRSDIEKRDKEITAYKNTLSAQQKENENKRTEAGKRLSAAIAAREKDENTLKTLR

AGNADAADITRQEFRLLQAELREYGFRTEIAGYDALRLHTESRMLFADADSLRISPREAR

SLIEQAEKRQKDAQNADKKAADMLAEYERRKGILDTRLSELEKNGGAALAVLDAQQARLL

GQQTRNDRAISEARNKLSSVTESLKTARNALTRAEQQLTQQKNTPDGKTIVSPEKFPGRS

STNHSIVVSGDPRFAGTIKITTSAVIDNRANLNYLLTHSGLDYKRNILNDRNPVVTEDVE

GDKKIYNAEVAEWDKLRQRLLDARNKITSAESAINSARNNVSARTNEQKHANDALNALLK

EKENIRSQLADINQKIAEEKRKRDEINMVKDAIKLTSDFYRTIYDEFGKQASELAKELAS

VSQGKQIKSVDDALNAFDKFRNNLNKKYNIQDRMAISKALEAINQVHMAENFKLFSKAFG

FTGKVIERYDVAVELQKAVKTDNWRPFFVKLESLAAGRAASAVTAWAFSVMLGTPVGILG

FAIIMAAVSALVNDKFIEQVNKLIGI

>BAC174

NRWYCNSAAGGVGGAAVCGLAGYVGEAKENIAGEVRKGWGMAGGFTHNKACKSFPGSGWA

SG

>BAC176

AGDPLADPNSQIVRQIMSNAAWGPPLVPERFRGMAVGAAGGVTQTVLQGAAAHMPVNVPI

PKVPMGPSWNGSKG

>BAC178

KGLGKLIGIDWLLGQAKDAVKQYKKDYKRWH

>BAC181

AVPAVRKTNETLD

>BAC182

GNGVVLTLTHECNLATWTKKLKCC

>BAC185

KPAWCWYTLAMCGAGYDSGTCDYMYSHCFGVKHSSGGGGSYHC

>BAC186

NETNNFAETQKEITTNSEATLTNEDYTKLTSEVKTIYTNLIQYDQTKNKFYVDEDKTEQY

YNYDDESIKGVYLMKDSLNDELNNNNSSNYSEIINQKISEIDYVLQGNDINNLIPSNTRV

KRSADFSWIQRCLEEAWGYAISLVTLKGIINLFKAGKFEAAAAKLASATAGRIAGMAALF

AFVATCGATTVS

>BAC187

PNWTKIGKCAGSIAWAIGSGLFGGAKLIKIKKYIAELGGLQKAAKLLVGATTWEEKLHAG

GYALINLAAELTGVAGIQANCF

>BAC189

SNDSLWYGVGQFMGKQANCITNHPVKHMIIPGYCLSKILG

>BAC190

IAPIIVAGLGYLVKDAWDHSDQIISGFKKGWNGGRRK

>BAC191

LIDHLGAPRWAVDTILGAIAVGNLASWVLALVPGPGWAVKAGLATAAAIVKHQGKAAAAA

W

>BAC192

LVATGMAAGVAKTIVNAVSAGMDIATALSLFSGAFTAAGGIMALIKKYAQKKLWKQLIAA

>BAC193

PNDGDTMTVSGGGGWVSNDDRKGGNDRDNGKGGSAVDFSKNPEKQAIVNPYLAIAIPMPV

YPLYGKLGFTINTTAIETELANVRAAINTKLATLSAVIGRSLPVVGRVFGVTAAGMWPSS

TAPSSLDSIYNQAHQQALAQLAAQQGVLNKGYNVTAMPAGFVSSLPVSEIKSLPTAPASL

LAQSVINTELSQRQLALTQPTTNAPVANIPVVKAEKTAMPGVYSAKIIAGEPAFQIKVDN

TKPALAQNPPKVKDDIQVSSFLSSPVADTHHAFIDFGSDHEPVYVSLSKIVTAEEEKKQV

EEAKRREQEWLLRHPITAAERKLTEIRQVISFAQQLKESSVATISEKTKTVAVYQEQVNT

AAKNRDNFYNQNRGLLSAGITGGPGYPIYLALWQTMNNFHQAYFRANNALEQESHVLNLA

RSDLAKAEQLLAENNRLQVETERTLAEEKEIKRNRVNVSTFGTVQTQLSKLLSDFYAVTS

LSQSVPSGALASFSYNPQGMIGSGKIVGKDVDVLFSIPVKDIPGYKSPINLDDLAKKNGS

LDLPIRLAFSDENGERVLRAFKADSLRIPSSVRGVAGSYDKNTGIFSAEIDGVSSRLVLE

NPAFPPTGNVGNTGNTAPDYKALLNTGVDVKPVDKITVTVTPVADPVDIDDYIIWLPTAS

GSGVEPIYVVFNSNPYGGTEKGKYSKRYYNPDKAGGPILELDWKNVKIDHAGVDNVKLHT

GRFKASVENKVMIERLENILNGQITATDTDKRFYTHELRELNRYRNLGIKDGEVPSSIQE

ESAVWNDTHTATLEDYKINEKEQPLYTDAALQAAYEQELKDALGGKHG

>BAC196

TITLSTCAILSKPLGNNGYLCTVTKECMPSCN

>BAC197

TTPATTSSWTCITAGVTVSASLCPTTKCTSRC

>BAC198

ATYTRPLDTGNITTGFNGYPGHVGVDYAVPVGTPVRAVANGTVKFAGNGANHPWMLWMAG

NCVLIQHADGMHTGYAHLSKISVSTDSTVKQGQIIGYTGATGQVTGPHLHFEMLPANPNW

QNGFSGRIDPTGYIANAPVFNGTTPTEPTTPTTNLKIYKVDDLQKINGIWQVRNNILVPT

DFTWVDNGIAADDVIEVTSNGTRTSDQVLQKGGYFVINPNNVKSVGTPMKGSGGLSWAQV

NFTTGGNVWLNTTSKDNLLYGK

>BAC199

KPAWCWYTLAMCGAGYDSGTCDYMYSHCFGIKHHSSGSSSYHC

>BAC200

KNYGNGVYCNKHKCSVDWATFSANIANNSVAMAGLTGGNAGNK

>BAC202

KYYGNGVSCNSHGCSVNWGQAWTCGVNHLANGGHGVC

>BAC203

MLAKIKAMIKKFPNPYTLAAKLTTYEINWYKQQYGRYPWERPVA

>BAC204

ATYYGNGLYCNKQKHYTWVDWNKASREIGKIIVNGWVQH

>BAC205

FVYGNGVTSILVQAQFLVNGQRRFFYTPDK

>BAC206

VNYGNGVSCSKTKCSVNWGIITHQAFRVTSGVASG

>BAC208

TNYGNGVGVPDAIMAGIIKLIFIFNIRQGYNFGKKAT

>BAC209

MFLVNQLGISKSLANTILGAIAVGNLASWLLALVPGPGWATKAALATAETIVKHEGKAAA

IAW

>BAC210

MAAFMKLIQFLATKGQKYVSLAWKHKGTILKWINAGQSFEWIYKQIKKLWA

>BAC211

ACQCPDAISGWTHTDYQCHGLENKMYRHVYAICMNGTQVYCRTEWGSSC

>BAC212

AIKLVQSPNGNFAASFVLDGTKWIFKSKYYDSSKGYWVGIYEVWDRK

>BAC214

VTTSIPCTVMVSAAVCPTLVCSNKCGGRG

>BAC216

GNAACVIGCIGSCVISEGIGSLVGTAFTLG

>BAC218

ATPATPTVAQFVIQGSTICLVC

>BAC219

IGGALGNALNGLGTWANMMNGGGFVNQWQVYANKGKINQYRPY

>BAC222

VTSWSLCTPGCTSPGGGSNCSFCC

>BAC223

MKTILRFVAGYDIASHKKKTGGYPWERGKA

>BAC224

IVWLANKFGVHLTNHLTNSILNAVSNGSSLGSAFAVIAGVTLPGWAVAAVGALGATAA

>BAC225

VFHAYSARGNYYGNCPANWPSCRNNYKSAGGK

>BAC229

LTANLGISSYAAKKVIDIINTGSAVATIIALVTAVVGGGLITAGIVATAKSLIKKYGAKY

AAAW

>WP_061432710.1

MKNPTLLPKLTAPVERPAVTSSDLKQASSVDAAWLNGDNNWSTPFAGVNAAWLNGDNNWS

TPFAGVNAAWLNGDNNWSTPFAADGAE

>CAX48972.1

MASILELQDLEVERASSAADSNASVWECCSTGSWVPFTCC

>YP_003491235.1

MGPVVVFDCMTADFLNDDPNNAELSALEMEELESWGAWDGEATS

>WP_013079675.1

MTKKNATQAPRLVRVGDAHRLTQGAFVGQPEAVNPLGREIQG

>ACR33052.1

MSALAIEKSWKDVDLRDGATSHPAGLGFGELTFEDLREDRTIYAASSGWVCTLTIECGTV

ICAC

>sp|Q09T02.1|MICA_CLAMM

MNDILETETPVMVSPRWDMLLDAGEDTSPSVQTQIDAEFRRVVSPYMSSSGWLCTLTIEC

GTIICACR

>NP_391616.1

MKKAVIVENKGCATCSIGAACLVDGPIPDFEIAGATGLFGLWG

>sp|O87236.1|LANA1_LACLL

MNKNEIETQPVTWLEEVSDQNFDEDVFGACSTNTFSLSDYWGNNGAWCTLTHECMAWCK

>sp|O87237.1|LANA2_LACLL

MKEKNMKKNDTIELQLGKYLEDDMIELAEGDESHGGTTPATPAISILSAYISTNTCPTTK

CTRAC

>sp|O88038.1|LANSB_STRCO

MNLFDLQSMETPKEEAMGDVETGSRASLLLCGDSSLSITTCN

>AAK33966.1

MNNTIKDFDLDLKTNKKDTATPYVGSRYLCTPGSCWKLVCFTTTVK

>YP_444120.1

MRTLTLNELDSVSGGASGRDIAMAIGTLSGQFVAGGIGAAAGGVAGGAIYDYASTHKPNP

AMSPSGLGGTIKQKPEGIPSEAWNYAAGRLCNWSPNNLSDVCL

>AAL73241.1

MSNTQLLEVLGTETFDVQENLFTFDTTDTIVAESNDDPDTRFKSWSFCTPGCAKTGSFNS

YCC

>sp|Q2QBT0.1|LANNU_STRUB

MNNEDFNLDLIKISKENNSGASPRITSKSLCTPGCKTGILMTCPLKTATCGCHFG

>ANP43731.1

MLDVIKNRKKIEEKLELPEILLEEVEEHSAMGGINTWNTTATSTSIIISETFGNKGKVCT

YTVECVNNCRG

>CAA84399.1

MVTKYGRNLGLSKVELFAIWAVLVVALLLATANIYWIADQFGIHLATGTARKLLDAVASG

ASLGTAFAAILGVTLPAWALAAAGALGATAA

>CAA63706.1

MTNAFQALDEVTDAELDAILGGGSGVIPTISHECHMNSFQFVFTCCS

>sp|Q52053|Q52053_9ZZZZ

MLNKENQENYYSNKLELVGPSFEELSLEEMEAIQGSGDVQAETTPACFTIGLGVGALFSA

KFC

>BAD72777.1

MKEIQKAGLQEELSILMDDANNLEQLTAGIGTTVVNSTFSIVLGNKGYICTVTVECMRNC

SK

>ANP43734.1

MLKEEKLEKITGLIPESELEEHLSGESSGAGTPAITTAISAIIAATAQSPCPTSACSKSC

NK

>AAT87775.1

MERRMSFMKNSKDILTNVIEEVSEKELMEVAGGKKGSGWFATITDDCPNSVFVCC

>BAD05046.1

MSTKDFNLDLVSVSKTDSGASTRITSISLCTPGCKTGVLMGCNLKTATCNCSVHVSK

>AAN86036.1

MFLVAGALGVQTAAATTIVNVILNAGTLVTVLGIIASIASGGAGTLMTIGWATFKATVQK

LAKQSMARAIAY

>AAL15569.1

MSKFDDFDLDVVKVSKQDSKITPQWKSESVCTPGCVTGVLQTCFLQTITCNCHISK

>AAL15567.1

MTNMSKFDDFDLDVVKVSKQDSKITPQVLSKSLCTPGCITGPLQTCYLCFPTFAKC

>AAK32702.1

MNKDLNALTNPIDEKELEQILGGGDGVFRTISHECAMNTWMFIFTCCS

>ARW80050.1

MSMTMTLQQAVVDDEFRSVLLADPAAFGLSVESLPGAVERQDHEAIEAFTEAVVASEIYA

CASTCSFGPFTIACDGTTK

>BAB04173.1

MTNLLKEWKMPLERTHNNSNPAGDIFQELEDQDILAGVNGACAWYNISCRLGNKGAYCTL

TVECMPSCN

>NP_478384.1

MKNELGKFLEENELELGKFSESDMLEITDDEVYAAGTPLALLGGAATGVIGYISNQTCPT

TACTRAC

>NP_478383.1

MKSSFLEKDIEEQVTWFEEVSEQEFDDDIFGACSTNTFSLSDYWGNKGNWCTATHECMSW

CK

>NP_834755.1

MSEIKKALNTLEIEDFDAIEMVDVDAMPENEALEIMGASCTTCVCTCSCCTT

>AHJ59549.1

MSKGYKFTKEELVEAWKDPQVREKLKDLPKHPSGKALNELSEEELAEIQGASDVQPETTP

LCVGVIIGLTTSIKICK

>WP_015792833.1

MTEEMTLLDLQGMEQTETDSWGGSGHGGGGDSGLSVTGCNGHSGISLLCDL

>CAG43551.1

MEKVLDLDVQVKGNNNTNDSAGDERITSHLFCSFGCEKTGSFNSFCC

>AAC69560.1

MSKKQIMSNCISIALLIALIPNIYFIADKMGIQLAPAWYQDIVNWVSAGGTLTTGFAIIV

GVTVPAWIAEAAAAFGIASA

>WP_067999479.1

MSAQGKDPNEIRRRFEELPMEVFQLDGSGLPIESLTDGHGMTEVGASCTSCVCICSCCT

>EFI65094.1

MTNEEIIVAWKNPKVRGKNMPSHPSGVGFQELSINEMAQVTGGAVEQRATPATPATPWLI

KASYVVSGAGVSFVASYITVN

>EFI65095.1

MTNEEIIVAWKNPKVRGKNMPSHPSGVGFQELSINEMAQVTGGAVEQRATPTLATPLTPH

TPYATYVVSGGVVSAISGIFSNNKTCLG

>BAN83916.1

MTEKTQITDVQAFEDLVAKVQEMDGPAQASSTVAALAGLDAAELQNFLEEKSGISPDEEA

QGSVMAAAASIALHC

>WP_013079674.1

MTKTHRLIRLGDAQRLTQGTLTPGLPEDFLPGHYMPG

>SED43766.1

MTEQSEQTPTEYIPPMLVEVGEFTEDTLGNWHGTSPDWFFNYYW

>EGD17355.1

MDTSNNDARTTALDQDLIVLGVASLDTQGGPLAGEEMGGITTLGISQD

>EDY58505.1

MLISTTNGQGTPMTSTDELYEAPELIEIGDYAELTRCVWGGDCTDFLGCGTAWICV

>EFE76491.1

MQKSVGHNGRQPRRREGVMKQQKQQKKAYVKPSMFQQGDFSKKTAGYFVGSYKEYWSRRI

I

>WP_043998581.1

MDKKNILPHQGKPVLRTTNGKLPSHLAELSEEALGGAGMDASFFPCSYDGADASFFPVCS

YDGADASFFPCSYDDGDA

>CAP64339.1

MRITPMDKKNLLPNQGAPVIRGISGKLPSHLAELSEEALGGNGAEASATVSICAFDGAEA

SFTGCMCAFDGAEASITGCICAFDGAEASITGCICAFDGDEA

>sp|Q07642.1|LANSB_STRGR

MALLDLQAMDTPAEDSFGELRTGSQVSLLVCEYSSLSVVLCTP

>NP_604414.1

MISSHQKTLTDKELALISGGKTHYPTNAWKSLWKGFWESLRYTDGF

>NP_345049.1

MNTKMLSQLEVMDTEMLAKVEGGYSSTDCQNALITGVTTGIITGGTGAGLATLGVAGLAG

AFVGAHIGAIGGGLTCLGGMVGDKLGLSW

>AAB91455.1

MNTITICKFDVLDAELLSTVEGGYSGKDCLKDMGGYALAGAGSGALWGAPAGGVGALPGA

FVGAHVGAIAGGFACMGGMIGNKFN

>ZP_04066356.1

MRTMEEQIFNSMIQQGAFAALFVWMLFTTQKKNEQREEQYQKVIEKNQQVIEEQAKAFSS

LSKDLSDVKQKILGNGDEK

>ZP_04066940.1

MRTMEEQIFNSMIQQGAFAALFVWMLFTTQKKNEQREEQYQKVIEKNQDVITKQAEAFGD

LSKDVSEIKQKILGSGDVQ

>NP_345050.1

MDTKMMSQFSVMDTEMLACVEGGGCNWGDFAKAGVGGGAARGLQLGIKTGTWQGAATGAA

GGAILGGVAYAATCWW

>NP_345056.1

MDTKIMEQFHEMDITMLSSIEGGKNNWQTNVLEGGGAAFGGWGLGTAICAASGVGAPFMG

ACGYIGAKFGVDLWAGVTGATGGF

>NP_345057.1

MNTYCNINETMLSEVYGGNSGGAAVVAALGCAAGGVKYGRLLGPWGAAIGGIGGAVVCGY

LAYTATS

>NP_345058.1

MDTKMMSQFAVMDNEMLACVEGGDIDWGRKISCAAGVAYGAIDGCATTV

>YP_140101.1

MATQTIENFNTLDLETLASVEGGGCSWGGFAKQGVATGVGNGLRLGIKTRTWQGAVAGAA

GGAIVGGVGYGATCWW

>AAG29818.1

MNTKTFEQFDVMTDEALSTVEGGGKGYCKPVYYAANGYSCRYSNGEWGYVVTKGAFQATT

DVIANGWVSSLGGGYFGKP

>AAC95138.1

MHKVKKLNNQELQQIVGGYSSKDCLKDIGKGIGAGTVAGAAGGGLAAGLGAIPGAFVGAH

FGVIGGSAACIGGLLGN

>AAC95139.1

MKKELLNKNEMSRIIGGKINWGNVGGSCVGGAVIGGALGGLGGAGGGCITGAIGSIWDQW

>AAG28763.1

MKKIEKLTEKEMANIIGGKYYGNGVTCGKHSCSVDWGKATTCIINNGAMAWATGGHQGTH

KC

>NP_297555.1

MRELTLTEIDNVSGADLGSRLSAAIVGGVAAFFAGSIWGGTRGGDGGGILGVGSIGQGVG

MVYGGIAGAIGGAIAGFVLDKDVIYSYTNGFMSSIFNGTFAK

>CAA90906.1

MIKREKNRTISSLGYEEISNHKLQEIQGGKGILGKLGVVQAGVDFVSGVWAGIKQSAKDH

PNA

>AAZ29031.1

MKKQILKGLVIVVCLSGATFFSTPQQASAAAPKITQKQKNCVNGQLGGMLAGALGGPGGV

VLGGIGGAIAGGCFN

>AAZ29032.1

MKIKWYWESLIETLIFIIVLLVFFYRSSGFSLKNLVLGSLFYLIAIGLFNYKKINK

>ZP_03980216.1

MTNFGTKVDAATRSYDNGIYCNNSKCWVNWGEAKENIAGIVISGWASGLAGMGH

>AAF44686.1

MKHLKILSIKQTQLIYGGTTHSGKYYGNGVYCTKNKCTVDWAKATTCIAGMSIGGFLGGA

IPGKC

>NP_863263.1

MKNIKNASNIKVIEDNELKAITGGGPGKWLPWLQPAYDFVTGLAKGIGKEGNKNKWKNV

>AAD28234.1

MQNVKELSTKEMKQIIGGENDHRMPNELNRPNNLSKGGAKCGAAIAGGLFGIPKGPLAWA

AGLANVYSKCN

>AAQ95741.1

MKKLTSKEMAQVVGGKYYGNGVSCNKKGCSVDWGKAIGIIGNNSAANLATGGAAGWKS

>BAA07120.1

MISMISSHQKTLTDKELALISGGKTYYGTNGVHCTKKSLWGKVRLKNVIPGTLCRKQSLP

IKQDLKILLGWATGAFGKTFH

>BAA82353.1

MKNFNTLSFETLANIVGGRNNWAANIGGVGGATVAGWALGNAVCGPACGFVGAHYVPIAW

AGVTAATGGFGKIRK

>NP_964623.1

MKLNDKELSKIVGGNRWGDTVLSAASGAGTGIKACKSFGPWGMAICGVGGAAIGGYFGYT

HN

>NP_542216.1

MDNLNKFKKLSDNKLQATIGGGMSGYIQGIPDFLKGYLHGISAANKHKKGRLGY

>NP_542217.1

MESNKLEKFANISNKDLNKITGGGFWGGLGYIAGRVGAAYGHAQASANNHHSPING

>YP_288875.1

MNALKRTCATLLISAGLTAGAVGVAAAAVEYVGGGIWDHGLTSSIVYSDYYHGSVCHGST

AVGTKTVRASAPAGYWSLADAPRAIANNQAYWRTTC

>NP_862432.1

MKTKSLVLALSAVTLFSAGGIVAQAEGTWQHGYGVSSAYSNYHHGSKTHSATVVNNNTGR

QGKDTQRAGVWAKATVGRNLTEKASFYYNFW

>ACR43769.1

MKELSEKELRECVGGGTWDDIGQGIGRVAYWVGKAMGNMSDVNQASRINRKKKH

>NP_268769.1

MIKFAEEIQKEELFHIIGGYSATDCKNHLIGGITSGAIAGGVGAGMATLGVGGVAGAFAG

AHVGAIAGGLTCVGGMLFNGK

>ACR43770.1

MKNNNNFFKGMEIIEDQELVSITGGKKWGWLAWVDPAYEFIKGFGKGAIKEGNKDKWKNI

>AAT72009.2

MNKTKSEHIKQQALDLFTRLQFLLQKHDTIEPYQYVLDILETGISKTKHNQQTPERQARV

VYNKIASQALVDKLHFTAEENKVLAAINELAHSQKGWGEFN

>NP_784211.1

MKIQIKGMKQLSNKEMQKIVGGKSSAYSLQMGATAIKQVKKLFKKWGW

>NP_784217.1

MLQFEKLQYSRLPQKKLAKISGGFNRGGYNFGKSVRHVVDAIGSVAGIRGILKSIR

>NP_784216.1

MKKFLVLRDRELNAISGGVFHAYSARGVRNNYKSAVGPADWVISAVRGFIHG

>NP_784205.1

MTVNKMIKDLDVVDAFAPISNNKLNGVVGGGAWKNFWSSLRKGFYDGEAGRAIRR

>ZP_04015571.1

MKIKLTVLNEFEELTADAEKNISGGRRSRKNGIGYAIGYAFGAVERAVLGGSRDYNK

>NP_784207.1

MKSLDKIAGLGIEMAEKDLTTVEGGKNYSKTWWYKSLTLLGKVAEGTSSAWHGLG

>AAG02566.1

MTKTSRRKNAIANYLEPVDEKSINESFGAGDPEARSGIPCTIGAAVAASIAVCPTTKCSK

RCGKRKK

>AAX99121.1

MKKKFVSSCIASTILFGTLLGVTYKAEAATVHVAGGVWSHGIGKHYVWSHYSHNKRNHGS

TAVGKYSSFSGVARPGVQSKASAPKAWGGNKTFYSLH

>NP_345048.1

MNTKMMEQFSVMDNEELEIVSGGRGNLGSAIGGCIGAVLLAAATGPITGGAATLICVGSG

IMSSL

>NP_720757.1

MNTKMMEQFETMDAETLSHVTGGGLYDGANGYAYRDSQGHWAYKVTKTPAQALTDVVVNS

WASGAASFAAYA

>YP_279852.1

MILFFMIFCTSSRLQRDKFKNYEKKLFDMEIKKLETFHQMTIEKLAKVEGGKNNWQANVS

GVIAAGSAGAAIGFPVCGVACGYIGAKTAITLWAGVTGATGGF

>AAP44569.1

MEAIKKLDLQAMKGIVGGKYYGNGLSCNKSGCSVDWSKAISIIGNNAVANLTTGGAAGWK

S

>AAP44566.1

MKNVQSLSKEELVLVVGGYTAKQCLQAIGSWGIAGTGAGAAGGPAGAFVGAHVGVIAGSA

VCIGGFLGQ

>AAP44567.2

MKTANIKLLTNQEMIEIFGGKTNWGSVVGSCVAGGLVGALGGTPISIGAGCLVGAGQDWI

SQK

>AAY68489.1

METAVAYYKDGVPYDDKGQVIITLLNGNPDGSGSGSGGGGGTGGSKSESSAAIHATAKWS

TAQLKKTQAEQAARAKAAAEAQAKAKANRDALTQHLKDIVNEALRHNSTHPEVIDLAHAN

NAAMQAEAERLRLAKAEEKARKEAEAAEKAFQEAEQRRKEIEKEQAETERQLKLAEDEEK

RLAALSEEARAVEVAQKNLAAAQSELAKVDEEINTLNTRLSSSIHARDAETNTLSGKRNE

LDQASAKYKELDERVKLLSPRANDPLQSRPFFEATRLRARAGDEMEEKQKQVTASETRLN

QISSEINGIQEAISQANNKRSTAVSRIHDAEDNLKTAQTNLLNSQIKDAVDATVSFYQTL

SEKYGEKYSKMAQELADKSKGKKISNVNEALAAFEKYKDVLNKKFSKADRDAIFNALEAV

KYEDWAKHLDQFAKYLKITGHVSFGYDVVSDILKIKDTGDWKPLFLTLEKKAVDAGVSYV

VVLLFSVLAGTTLGIWGIAIVTGILCAFIDKNKLNTINEVLGI

>YP_025360.1

MSGGDGKGHNSGAHDSGGSINGTSGKGGPDSGGGYWDNHPHITITGGREVGQGGAGINWG

GGSGHGNGGGSVAIQEYNTSKYPNTGGFPPLGDASWLLNPPKWSVIEVKSENSAWRSYIT

HVQGHVYKLTFDGTGKLIDTAYVNYEPSDDTRWSPLKSFKYNKGTAEKQVRDAINNEKEA

VKDAVKFTADFYKEVFKVYGEKAEKLAKLLADQAKGKKVRNVEDALKSYEKYKTNINKKI

NAKDREAIAKALESMDVGKAAKNIAKFSKGLGWVGPAIDITDWFTELYKAVETDNWRSFY

VKTETIAVGLAATHVAALAFSAVLGGPVGILGYGLIMAGVGALVNETIVDEANKVIGL

>sp|Q47502.1|CEAK_ECOLX

MAKELSGYGPTAGESMGGTGANLNQQGGNNNSNSGVHWGGGSGHGNNGGQGNSNSSGSTS

TVMKTGESYLTPWGDVVINNDGLPVMNGIVMTEENSTLVDNPFGGVSRVLNSLISDMPSL

FAESSGNNNNNTASVNTAPTNAQVSDMDKSSKVVSNVINEKQKQKNKIATQISEKQKKIE

EMKKVFKHHSYHGITDLERDVDELQKKSNQLDADISKLNSYKNTLQSKIGDVNKQKEAEE

KARENAEVAEHETLNEEKQAVAEAEKRLAEAKAELAKAESDVQSKQATVSRVAGELENAQ

KSVDVKVTGFPGWRDVQKKLQRQLEAKQAEYSAVENELKNAVSFRDGKAAEVKEAEQKLK

EAQDALEKSQIKDAVDTMVGFYQYITEQYGEKYAKIAQDLAEKSKGKKIQGVDEALAAFE

KYKNVLDKKFSKVDRDAIFNALESVNYDELSKNLTKISKSLKITSRVSFLYDVGSDFKNA

IETGNWRPLFVTLEKSAVDVGVAKIVALMFSFIVGVPLGFWGIAIVTGIVSSYIGDDELS

KLNELLGI

>AAD35867.1

MVNMEFLKRSFAPLTEKQWQEIDNRAREIFKTQLYGRKFVDVEGPYGWEYAAHPLGEVEV

LSDENEVVKWGLRKSLPLIELRATFTLDLWELDNLERGKPNVDLSSLEETVRKVAEFEDE

VIFRGCEKSGVKGLLSFEERKIECGSTPKDLLEAIVRALSIFSKDGIEGPYTLVINTDRW

INFLKEEAGHYPLEKRVEECLRGGKIITTPRIEDALVVSERGGDFKLILGQDLSIGYEDR

EKDAVRLFITETFTSRLSTRRP

>CAA44310.1

MPGFNYGGKGDGTNWSSERGTGPEPGGGDKGHSGDRDRGGAGVGNSPEQQQIAAIQNDPA

LRMKLEAVIKAARRINPDAKLHIESVSPSGTLSLSATGLTADQAKHIGLGGLVMGVNAKG

VTVAIGDIETGHARKPSPPGKGGNNGLNAGQIGASSLGSFVTDSHRDRPVSGWHGNGKTG

EFSTTRTTGSYYGFHHLKVEKQDGLATYSLYYKANKNRPAFIAVVRGDNLNAMEVKYANG

KPVKSPGSVKTIVKEFVEYQNAELKAIKDGVSLAAGINKDIAEKIGAKYAKLAKDLEAGI

QGKYIRNVQDAEKTYEQLTKGLNKKLKAQDKAAIVAWLKMIDAEQYARNARVLGKVFTGV

DWAIKGADLVNAAIEGFSTGNWKAFRNQLEALGLSIGAGYTLSAIAAFFAPTLVSSTVGI

FAFAYLFGWATSYIDAERAGELEKWVADL

>CAA72509.1

MPGFNYGGHGDGTGWSSERGDGPAPGGGMQGNGGGHSGNNDSGSNSVSQQISAIQNDQKL

KQKVVNMLIAARKMNPDAKMILGSIAPSGVMQVTIEGVTSTQARQLGLGGLVMGYNASGV

IGAVGEIDTGHRLNASGASTPGSETSVDSFVNGQKPAEEWHAVAKDSWTGAGPVNTGLVN

NAIKSVRIIKKGYVTGVLTPEEVMNKAEYKAMRQAFDSLPLAKQGEAVRQIVAAWSLAYQ

DFPVNLKKEMGRVTERIVDAINLALILNQTESRLSESQKNVDVANQIISDTVKAINDVNK

KIAEKRNQQVSLTDLMNKKQKEVEDLKKIFKNHSYHRIRDAQREYDDARNKYALLASDIN

ALQAQVSGLTARKQQAEQNKAAAEKAKADAAAKAAAEKAAAEAKAKAEAEKARKEAEEKA

NDEKAVLTKASEIIISVGDKAGEYLGDKYKVLSREIADNIKNFQGKTIRSYDEAMASVNK

LMANPDLKINAADRDAIVNAWKAFDAEDMGNKFAALGKTFKAADYVMKANNVREKSIEGY

QTGNWGPLMLEIESWVLSGIASAVALSFFSAIFGTFAMLGVFSTSLAGILAVILAGLVGA

LIDDNFVDKLNNEIIRPAY

>NP_061654.1

MVCFKSQRGMNMPGFNYGGYGDGTGWSSESGGPAPGGGMHGNSGGQRGDNANSSNSVSQQ

ISAIQNDQKLKQKVVNMLIAARKMNPEAKMILGSIAPSGVMQVTIEGVTSTQAKQLGLGG

LVMGYNASGVIGAVGEIDTGHRLNASGASTPGSETSVESFVNGQKPAGEWHAVAKDSWTG

AGPVNVGLVNNAIKSVRIIKKGYVTGVLLPEEVMNKAEYKAMRQAFDSLPLAKQGEAVRQ

IVAAWSLAYQDFPVNLKKDMGRVTERVVDAVNLALILNQISSGMSASQKDVDAANRIINE

TVKAINDVNLKIAEKKKQQVPLLSLMKQKQKEVEELKKVFKNHSYHRIRDAQRAYDDARN

KNDLLVSDINALQAQVSGLTARKQQAEKNKAAAEKAKADAKAKAEAEKAAAEAKAKAEAE

KARKEAEAKANDEKAVLTKASEIIISVGDKVGEYLGDKYKALSREIAGNIKNFQGKTIRS

YDEAIASVNKLMANPDLKINAADRDVIVNAWKAFDAEDMGNKFAALGKTFKAADYVMKAN

NVREKSIEGYQTGNWGPLMREVESWVVSGIASAVALAIFSATLGAYLLAVGASAAVVGII

GIIIASFIGALIDDKFIDRLNNEIIRPAY

>sp|P04480.1|CEA_CITFR

MPGFNYGGKGDGTGWSSERGSGPEPGGGSHGNSGGHDRGDSSNVGNESVTVMKPGDSYNT

PWGKVIINAAGQPTMNGTVMTADNSSMVPYGRGFTRVLNSLVNNPVSPAGQNGGKSPVQT

AVENYLMVQSGNLPPGYWLSNGKVMTEVREERTSGGGGKNGNERTWTVKVPREVPQLTAS

YNEGMRIRQEAADRARAEANARALAEEEARAIASGKSKAEFDAGKRVEAAQAAINTAQLN

VNNLSGAVSAANQVITQKQAEMTPLKNELAAANQRVQETLKFINDPIRSRIHFNMRSGLI

RAQHNVDTKQNEINAAVANRDALNSQLSQANNILQNARNEKSAADAALSAATAQRLQAEA

ALRAAAEAAEKARQRQAEEAERQRQAMEVAEKAKDERELLEKTSELIAGMGDKIGEHLGD

KYKAIAKDIADNIKNFQGKTIRSFDDAMASLNKITANPAMKINKADRDALVNAWKHVDAQ

DMANKLGNLSKAFKVADVVMKVEKVREKSIEGYETGNWGPLMLEVESWVLSGIASSVALG

IFSATLGAYALSLGVPAIAVGIAGILLAAVVGALIDDKFADALNNEIIRPAH

>sp|P05819.3|CEAB_ECOLX

MSDNEGSVPTEGIDYGDTMVVWPSTGRIPGGDVKPGGSSGLAPSMPPGWGDYSPQGIALV

QSVLFPGIIRRIILDKELEEGDWSGWSVSVHSPWGNEKVSAARTVLENGLRGGLPEPSRP

AAVSFARLEPASGNEQKIIRLMVTQQLEQVTDIPASQLPAAGNNVPVKYRLTDLMQNGTQ

YMAIIGGIPMTVPVVDAVPVPDRSRPGTNIKDVYSAPVSPNLPDLVLSVGQMNTPVRSNP

EIQEDGVISETGNYVEAGYTMSSNNHDVIVRFPEGSGVSPLYISAVEILDSNSLSQRQEA

ENNAKDDFRVKKEQENDEKTVLTKTSEVIISVGDKVGEYLGDKYKALSREIAENINNFQG

KTIRSYDDAMSSINKLMANPSLKINATDKEAIVNAWKAFNAEDMGNKFAALGKTFKAADY

AIKANNIREKSIEGYQTGNWGPLMLEVESWVISGMASAVALSLFSLTLGSALIAFGLSAT

VVGFVGVVIAGAIGAFIDDKFVDELNHKIIK

>sp|P17998.1|CEAD_ECOLX

MSDYEGSGPTEGIDYGHSMVVWPSTGLISGGDVKPGGSSGIAPSMPPGWGDYSPQGIALV

QSVLFPGIIRRIILDKELEEGDWSGWSVSVHSPWGNEKVSAARTVLENGLRGGLPEPSRP

AAVSFARLEPASGNEQKIIRLMVTQQLEQVTDIPASQLPAAGNNVPVKYRLMDLMQNGTQ

YMAIIGGIPMTVPVVDAVPVPDRSRPGTNIKDVYSAPVSPNLPDLVLSVGQMNTPVLSNP

EIQEEGVIAETGNYVEAGYTMSSNNHDVIVRFPEGSDVSPLYISTVEILDSNGLSQRQEA

ENKAKDDFRVKKEEAVARAEAEKAKAELFSKAGVNQPPVYTQEMMERANSVMNEQGALVL

NNTASSVQLAMTGTGVWTAAGDIAGNISKFFSNALEKVTIPEVSPLLMRISLGALWFHSE

EAGAGSDIVPGRNLEAMFSLSAQMLAGQGVVIEPGATSVNLPVRGQLINSNGQLALDLLK

TGNESIPAAVPVLNAVRDTATGLDKITLPAVVGAPSRTILVNPVPQPSVPTDTGNHQPVP

VTPVHTGTEVKSVEMPVTTITPVSDVGGLRDFIYWRPDAAGTGVEAVYVMLNDPLDSGRF

SRKQLDKKYKHAGDFGISDTKKNRETLTKFRDAIEEHLSDKDTVEKGTYRREKGSKVYFN

PNTMNVVIIKSNGEFLSGWKINPDADNGRIYLETGEL

>sp|P09883.4|CEA9_ECOLX

MSGGDGRGHNTGAHSTSGNINGGPTGIGVSGGASDGSGWSSENNPWGGGSGSGIHWGGGS

GRGNGGGNGNSGGGSGTGGNLSAVAAPVAFGFPALSTPGAGGLAVSISASELSAAIAGII

AKLKKVNLKFTPFGVVLSSLIPSEIAKDDPNMMSKIVTSLPADDITESPVSSLPLDKATV

NVNVRVVDDVKDERQNISVVSGVPMSVPVVDAKPTERPGVFTASIPGAPVLNISVNDSTP

AVQTLSPGVTNNTDKDVRPAGFTQGGNTRDAVIRFPKDSGHNAVYVSVSDVLSPDQVKQR

QDEENRRQQEWDATHPVEAAERNYERARAELNQANEDVARNQERQAKAVQVYNSRKSELD

AANKTLADAIAEIKQFNRFAHDPMAGGHRMWQMAGLKAQRAQTDVNNKQAAFDAAAKEKS

DADAALSAAQERRKQKENKEKDAKDKLDKESKRNKPGKATGKGKPVGDKWLDDAGKDSGA

PIPDRIADKLRDKEFKSFDDFRKAVWEEVSKDPELSKNLNPSNKSSVSKGYSPFTPKNQQ

VGGRKVYELHHDKPISQGGEVYDMDNIRVTTPKRHIDIHRGK

>AAG29099.1

MKNILLSILGVLSIVVSLAFSSYSVNAASNEWSWPLGKPYAGRYEEGQQFGNTAFNRGGT

YFHDGFDFGSAIYGNGSVYAVHDGKILYAGWDPVGGGSLGAFIVLQAGNTNVIYQEFSRN

VGDIKVSTGQTVKKGQLIGKFTSSHLHLGMTKKEWRSAHSSWNKDDGTWFNPIPILQGGS

TPTPPNPGPKNFTTNVRYGLRVLGGSWLPEVTNFNNTNDGFAGYPNRQHDMLYIKVDKGQ

MKYRVHTAQSGWLPWVSKGDKSDTVNGAAGMPGQAIDGVQLNYITPKGEKLSQAYYRSQT

TKRSGWLKVSADNGSIPGLDSYAGIFGEPLDRLQIGISQSNPF

>AAT85004.1

MADNQPVPLTPAPPGMVSLGVNENGEEEMTVIGGDGSGTGFSGNEAPIIPGSGSLQADLG

KKSLTRLQAESSAAIHATAKWTTENLAKTQAAQAERAKAAMLSQQAAKAKQAKLTLHLKD

VVDRALQNNKTRPTVIDLAHQNNQQMAAMAEFIGRQKAIEEARKKAEREAKRAEEAYQAA

LRAQEEEQRKQAEIERKLQEARKQEAAAKAKAEADRIAAEKAEAEARAKAEAERRKAEEA

RKALFAKAGIKDTPVYTLEKTKAATTLFLTPGVRLLNRAPAMIQLSALAAEINGVLTTAA

SAVMTATAEFSGWIASALWRGVAGVATASTVGPMVAAASTLFFSPRAGGGSDSKVPGRDI

EMLAAQARLFTAGKLSIEPGMKSVNLPVRGFISSETDGRQSLMLVKTGSDGVPSTVPVLD

AVRDSTTGLDKITVPAMSGAPSRTILVNPVPIGPAAPWHTGNSGPVPVTPVHTGTEVKQA

DSIVTTTLPIADIPPLQDFIYWQPDASGTGVEPIYVMTSQPRKGVKDYGHDYHPAPKTEE

IKGLGELIESRKKTPKQGGGGRRDRWVGDKGRKIYEWDSQHGELEGYRASDGSHLGAFDP

NTGKQLKGPDPKRNIKKYL

>AAL73547.1

MKTNNVTGTMKKVISTLAATGCMFSMAAAIPANSTIGSAVLGNAVVADAAVISVNTVVDA

KNGNADLVQGKFYKSPSQNYVLVFQNDGNLVIYHYNKTTDKAYSPIWSSQTENRGGTKCV

LQGDGNFVIYRSDGKPIWNTQTNGKKGAYLTISDEGEIKITSRNYNYATTWSSKNNHGYS

INQGPIITDPVDGQLSPHFHSREFACDCGNTHTIDQNLINKLEQLYTKLNCSKIIVNSGY

RDPNCSVAVGGGYDDAHTRGLAADVVCYDKNGNVIPCLTVAWAAEQIGFTGIGLMYGGAI

HLDVRTTSNYKNGHWFGDERKEYKNDYISTFKNYVPHKA

>AAT90329.1

MSDITYNPEDYNNGIPPEPGLVWKPGGSFPNGSYVPGSWGWPTRGYDVPPLPGDTEMLTV

TPKGTPADTWPKRPDIKEWYVPGEKPFDPSTGNGWVPDVDGYAESLPAGIPAVVQAAISK

VKGAPLKGGMSAVDIWKLKPATEYPGRFNSTDPAFSWFPVRALTDTDISAMPVAPETVPV

HTRILDNVHDGVQFVSAVFAGSMQYNLPVVKAQATAGSDYYTIGRLPGIMSAFTFSFYTK

GTPQDSRFFRDTVKAGGDLREAGFTVGANTSDFIIWFPQGSGLEPLYFSMTMNMPAGPLQ

RRQEAENKARAEADRLRAEAEAKIRAEAEARAKAEAERKALFAKAGIQDTPVYTPEMVKA

ANAALSAGGSMALSRAPGMIQHSAAGVGTLPFNSSLAGWEAGALWRGVDVLARIAPVASA

VATVATVLTLVRAALDIPAAGEGSDRVPGRNIDMLAAQASLYTAMKTNIQPGMKTVDLPV

RGYISYDGNGRQSVNLVRTGTGGVSATVPVLSAVRDKTTGLDKITVPAVAGAPSRTILIN

PVPVGPATPSHTGSSTPVPVTPVHTGTDVKQADSIVTTTLPAADIPALQDFIYWQPDATG

TGVEPIYVMLSDPLDSGKYTRRQLQKKYKHAIDFGITDTKINGETLTKFRDAIEAHLSDK

DTFEKGTYRRDKGSKVYFNPKTMNAVIIQANGDFLSGWKINPAADNGRIYLETGDL

>NP_889019.1

MNNLYRDLAPISAAAWAQIEEEVARTFKRSVAGRRVVDVKDPGGFGLAAVGTGHLRGIAA

PQKGVDAKLREVKALVELTVPFELQRDEIDAVERGANDADWQPAKDAATELAYAEDRAIF

DGYKAAGIVGIREGSSNSRLELPTDAADYPAAVGRALEQLRLAGVDGPYSVLLGADAYTA

LSEGSDDGYPTIDHIKRIVSGDIIWAPALNGGCVLSTRGGDFELHLGQDLSIGYQSHTDK

VVRLYLRETLTFLMLTSEASVPVAPKG

>CAE09438.1

MDILRRENAQFPASIWSAIEKEAGLVFGKHLTGRKVVDFKGGLGIGFSSLPTGRVISSKE

KLGEASVGVRMNTPVIELKIPFSFPESEVEAILREANAFDISSIEKAAKKVCVAENELVF

YGLKKEGIEGLIPSIPHKPIKAKGDEILPAVAEGIKELVNSEIEGPYALLIQPQYFGKLF

GVAGNSGYPLTLKLAELLQGNNIIVAPALKSGALLVSLRGGDYELYSGMDIGVGYSEKKS

TNHELFFFETLTFRINTPEASIAIEW

>YP_426062.1

MNDLMRDLAPISAKAWAEIETEARGTLTVTLAARKVVDFKGPLGWDASSVSLGRTEALAE

EPKAAGSAAVVTVRKRAVQPLIELCVPFTLKRAELEAIARGASDADLDPVIEAARAIAIA

EDRAVFHGFAAGGITGIGEASAEHALDLPADLADFPGVLVRALAVLRDRGVDGPYALVLG

RTVYQQLMETTTPGGYPVLQHVRRLFEGPLIWAPGVDGAMLISQRGGDFELTVGRDFSIG

YHDHDAQSVHLYLQESMTFRCLGPEAAVPLRGLSQAATKA

>YP_366690.1

MNNLHRELAPISSSAWEQIEEEVARTFKRSVAGRRVVDVDGPEGPELSAVGTGHLVEVAA

PREQVNARLREVRTIVELTVPFELSRDAIDSVERGARDADWQPAKDAAQRLAFAEDGAIF

DGYAAASIVGIREGTSNNKLTLPADVSAYPDAISDALEALRLAGVDGPYSVVLGSDAYTA

LSEARDQGYPVLGHIKRIVSGEIIWAPAISGGCVLSTRGGDYELHLGEDVSIGYTSHTDK

VVRLYLRETFTFLMLTSEASVAVAPQANTTA

>sp|Q45296.1|LIN18_BRELN

MNNLYRELAPIPGPAWAEIEEEARRTFKRNIAGRRIVDVAGPTGFETSAVTTGHIRDVQS

ETSGLQVKQRIVQEYIELRTPFTVTRQAIDDVARGSGDSDWQPVKDAATTIAMAEDRAIL

HGLDAAGIGGIVPGSSNAAVAIPDAVEDFADAVAQALSVLRTVGVDGPYSLLLSSAEYTK

VSESTDHGYPIREHLSRQLGAGEIIWAPALEGALLVSTRGGDYELHLGQDLSIGYYSHDS

ETVELYLQETFGFLALTDESSVPLSL

>CAA90860.1

MSDTMVVNGSGGVPAFLFSGSTLSSYRPNFEANSITIALPHYVDLPGRSNFKLMYIMGFP

IDTEMEKDSEYSNKIRQESKISKTEGTVSYEQKITVETGQEKDGVKVYRVMVLEGTIAES

IEHLDKKENEDILNNNRNRIVLADNTVINFDNISQLKEFLRRSVNIVDHDIFSSNGFEGF

NPTSHFPSNPSSDYFNSTGVTFGSGVDLGQRSKQDLLNDGVPQYIADRLDGYNMLRGKEA

YDKVRTAPLTLSDNEAHLLSNIYIDKFSHKIEGLFNDANIGLRFSDLPLRTRTALVSIGY

QKGFKLSRTAPTVWNKVIAKDWNGLVNAFNNIVDGMSDRRKREGALVQKDIDSGLLK

>YP_050090.1

MFTDEIIWHDVITKYSVNNLSQDMLNDPSETMFVLGDVYKEQALEYYGYLRSELLKSKEL

ISNAEKSLIIALESRVKAEQDKKSADQKLKDEQEKDKGKAPELKLDDKIREQLGNRGWTE

QDVRDTVSKGAKGSAEDKCSPKKTPPDFLGRNDPASVYGEFGKYIVVNDRTGEVVQFSDK

SDPEWVDDSRINWGDKNE

>AAM95702.1

MAGRTRIPFNGVGTSVLPAYQTLSAGQYLLSPNQRFKLLLQGDGNLVIQDNGATVWVANE

QQPFSSTIPLRNKKAPLAFYVQYGAFLDDYSRRRVWLTDNSTFTSNDQWNRTHLVLQDDG

NIVLVDSLALWNGTPAIPLVPGAIDSLLLAPGSELVQGVVYGAGASKLVFQGDGNLVAYG

PNGAATWNAGTQGKGAVRAVFQGDGNLVVYGAGNAVLWHSHTGGHASAVLRLQANGSIAI

LDEKPVWARFGFQPTYRHIRKINPDQKPIDIWTWHF

>prf||1912296A

MSDVFDLGSMTTVATATGQYSFYTPPPPTPIPYLTYIARPGINKFDLPEGAKIKDLIKRY

QYIGSQIPAAIMIRGVQEEIKKSTNTALANVGAIVDGELAYLASQKKEKLNPAEATPLQM

ASAEKAAAVELLASKQKELADARTIANAFFGYDPLTVNYVNVMNEIYGRREDKDFSFDNW

SKSYSAAQKIRLIEAKISVLNSRSSALDGKVAELTRLQRLEDAQHAAEAARQTEAERLAQ

EQRQAEARRQAEEARRQAEAQRQAELQRLAEAEAKRVAEAEKKRQDEINARLQAIVVSES

EAKRIEEIYKRLEEQDKISNPTVTTPPAVDAGSRVDDALAHTGTRVTSGGETGATGGSGR

DVDTGTGQGGITARPVDVGSVSIPDRRDPKIPDQPRRDLGSLVPTFPDFPTFPSFPGVGV

PAAAKPLIPAGGGAASVSRTLKTAVDLLSVARKTPGAMLGQVAAVVATMAVSSFWPKLNN

GERQASFAIPVAELSPPLAVDWQAIAAAKGTVDLPYRLKTLNVDGSIQIIAVPTEPGSAA

VPVRALTLDSASGTYKYTTTGPGGGTILVTPDTPPGQIDPSSSTPAVPRGPLIMPGTLLI

PKEPQIESYPELDQREFNDGIYVYPEDSGIPPLYIVYRDPRDEPGVATGNGQPVTGNWLA

GASQGDGVPIPSQIADQLRGKEFKSWRDFREQFWMAVSKDPSALENLSPSNRYFVSQGLA

PYAVPEEHLGSKEKFEIHHVVPLESGGALYNIDNLVIVTPKRHSEIHKELKLKRKEK

>AAA23073.1

RFAHDPMAGGHRMWQMAGLKAQRAQTDVNNKQAAFDAAAKEKSDADAALSAAQERRKQKE

NKEKDAKDKLDKESKRNKPGKATGKGKPVGDKWLDDAGKDSGAPIPDRIADKLRDKEFKN

FDDFRRKFWEEVSKDPELSKQFNPGNKKRLSQGLAPRARNKDTVGGRRSFELHHDKPISQ

DGGVYDMDNLRITTPKRHIDIHRGQ

**Non-bacteriocin**

**--------------------------------------------------------------**

>WP_001030800.1

MNKDSTQTWGLKRDITPCFGARLVQEGHRLHFLADRAGFTGSFSEVQTLQLDEAFPHFVA

HLELMLLSCELNPRYAHCVTLYRNGLTGEADTLGSHGYVYIAILNRHGFNRHLRVI

>WP_050443533.1

MNFEQMKAVYEMVKAIYNKEERLVIGKEKLHLTHGINKNSFADFYRAFQKMLDGELHTRG

ISTDLRDFYLSQIYEDYGTKKLETALNAYMDFIIYYEKKHNNIKKKNERKIYQKHYELIK

HQSPERKGRVKVVEFYEGEFEQVFITKHERNTEARNKCIQAKGVKCVVCDFDFEKTYGEL

GKGFIHVHHINPISTKDGNYAINIENELVPVCPNCHAMLHRRKDKILSIEELKRIFHNK

>WP_159117600.1

MAKDTPSMAKNKIKRCLWAIYDQHPKKSEVDSLWTYFESKCAYCGVEIERSSRTGHVDHL

IPSAEGGSNSIHNHVLACARCNGDEKREEDWLTFLSKKSGKSSIFEQRRSNIEEWLSLMP

PNGTNTALKSEVEKVVDKALKDFDSAVAQVRSLINVNDRG

>WP_130071231.1

MSRNPYYIKMINSQRWKNLRCDKLRANPVCEVCEANGLSTLATEVHHKSPVESVSHELGM

KHLMFDRTNLQSLCHACHSEIHRRVFSHSKEAIQANNRRATERFADKFLK

>WP_169167565.1

MKVLKLSAQGVPQSWITLEQAVIHSAAGDVRWVAGSEVAVFHGGHNAVTGLQSVIAVNSI

IGTRGVSRINPFELKPGLANNKLFARDRNVCAYCGGHFDEHDLTREHIVPLAQKGADQWM

NVVTACRPCNHRKGPRTPEQARMPLLYAPYVPSLWEDFILRNRRILADQMEFLAAHLPRS

SRLLN

>WP_160213701.1

MALFLLEWWRMAKPFSDAFYHSKAWGRAREDALKRDSYLCQRCLAGGEITPATMVHHIEE

LTPANIDNPDITCGLDNLVSLCDLCHKKTHGWARAGATRQGLAFDADGNLICLAE

>WP_160212293.1

MPSNNVRYRNWKARTEQRRRILRECDGRVCPFCGRPMDASLDWWTDPADGRRKRHPYSIE

VDEIVPVSKGGSPIDPANLQGAHRICNQRAGAKNRRPKPRGDVTGGGLPASREW

>WP_159494819.1

MLVLRLNKAGMPQEWIDVEHAAKLYSQEKVLFELGSDAITLKGGWNHEGLQSQLTLSSII

ACDGKVTDMSGKVALTNRFLFRRDSYLCLYCGQKFSPKQLTRDHIIPRSRGGKDTWTNVA

TACQRCNHAKAAKTPEEANMPLLAVPFRPNIYERFYLMNRRILSDQMAFLKGHFSHKRNW

TCLD

>WP_142428358.1

MIDVTSKQARAKFYGSSEWRRLRQQCLERDHYECQWCKQEGKLTTQYDSVLEVDHIKELE

HYPQHALDIDNLRTLCKDCHNKRHGRFNYRESKRKRKWDDEWW

>WP_142426822.1

MIEVTTKTDRAKFYSSSQWKKLRLKALERDHYECQWCKEQGKVTTINDAILEVDHIKELE

YHPEFATDIDNLRTLCKECHNKRHSRMNYRGAERKKKFDDEWWGD

>WP_142422844.1

MTDEFYRWLLQLIREDRLVKFYQSPKWRRLREKAMKRDHYECQECRRLGKYHRVENVHHI

KEVKDRPDLALDLDNLICLCVEHHNEVHGRYLTALDKQEKKIESFANFDASERW

>WP_142422626.1

MGKKWNTEMFSEFVNSTYPDFEVRGEYVSSKNNILIYHKKCDREFSVIARNFKTRGTCSL

CNGKFKSNTSEFKDKVNTLTNDEYEVIGEYVTCKDKIELAHKKCGTIYFATPDDFINGGT

RCPRCFGNNRKTSKRFKNEVFNLFKNEYIVLGEYKNNKTPLLMKHDSKKCNHEFMVSPDA

FLRGSHCNKCGTEKRSGENHYKYNFSLTEEDRMARDMQNGEIRKWRDKIYLRDDYTCQVC

RIKGYKLNAHHLNSWDFYERERFDTDNGITLCEDCHRKFHKKYGYGHNTKKQFTLYLEEN

KPTTSIL

>WP_121698255.1

MSERRISEKELILPTLYLAVCNGGRITTSELIKQLTAMMRPSGIDAEILSGRNDTYFSQK

VRNLRSHNTLVAPGYAIYDDKGYAITQLGRDFVEARMDSLRYLLSSDFDYEDVRGHLDDV

TDGKVIPYDELVSEGETITMTATSHERSRKLRDAAVAHYTQDGVLKCCCCGFDFGSFYGD

KYGSSCIEIHHIKPIFMYEGRSEEQTIEEALDNLMPVCPNCHRAIHRNHVMRDELPDFIA

AIKASRKS

>WP_081722951.1

MERGSPHTWEVVDVATSRTGTAQYKHWRKRVLIAARDAGIAQCPHCGVRLDYTRGLQPNS

AEPDHILPVRWGGKNTLENGRVLCRRCNQSRGDGTRPKVKPRRAASVDVDW

>WP_001372261.1

MIEKICEVIDGEYVCDIDISVEEWKILLRDKKVFDDKSIAALKKWFIEPDHSCTCFDIGK

KYDLHSMSANGVINGLGGRVQKQLGRFEVKGVGKIASGTKFITVMKSREIKGNPKRNLWT

IREELVQAIKELDFFSTNESSSIDFYSDNDLITALEESNHFDVTQTFEYSEKAKPKKAAI

EVKNGLSYPRSKSVSKNALNKADYKCEINCDHPTFRRRNSPLNYTEPHHIVPMSKQDYFE

NSLDVEENIISLCCNCHKQIHLGKGFEDMLRKIYAERKDVLKKAGIEILLEDLILFYKME

GN

>WP_102372778.1

MSGNSRNAAQPEFREGSRCEVTLDRYERSEAARKACIAAHGATCAICGFDFSHTYGPTFA

GIIQVHHIVPLHVTGKEHEVDPMHDLIPVCPNCHVALHSKPDGTYLPDELRALMR

>WP_160214295.1

MFGFSVTLYLFIAGMGAGLYIASCLVEREMERARPPRDMQLLHQKALIISLLLVCAGSAF

LILDLTVPQKMYLVFKRPFGSVISFGAWLIALLTLMLAVRNGFWRIFATSRSPLIRFLKA

ATFLLACGVTLYTGFFLIGLKAISFWESLLVVALFAISSLSSGIACFSVLAAFSLRRSTT

PPVVCKADQVDTFLLAAEIIVLGIFVVSQLFGDAASAASSSRLISGELAWAFWLMLVGIG

LLFPFGLSVFGRANHKLSPLVVKGISACIGCFFLRYCIMEAGVRSFSLA

>WP_160213053.1

MTYEPIWGPIIAWYLFLAGLGGGAFVTSVFIRFRHPECTRLIRTGRIIAPAVVIIGLCLL

MFDAHAGFMHPLRFALLLTNFGSVMTWGVVFLAAFVVLALVALLLDLLKKPVWQWLDIAG

MVMGLCVAIYTGCLLGVCQGFPLWNNALLPVLFLVSAVSTGMAAVLLAGVFVAPEEFNAV

VSLKKFHFWLPVVEIALVMALLFITASNPSPAGWNSVVTLLCGDWAVAFWVLFIAVGLVI

PIALECWMLWIATPVVEESRTGQMISGFSDLGVLVGGFVLRLMIVSAALPITIVQPWIF

>WP_160213014.1

MQLLTKNKLLTGIFAVLGVAGIAAWAYQLAGGLGVTGMSNANSWGLYIAMFMFFVGLSAG

GLIVASSASVFHTTEYKKVAMPAIILSTVCICCAGAFVLIDLGGIQRIWHLFASPNVASP

LVWDICVITLYLVINVLYLRFMHKGAERAVSVLSRFALPTAVLVHSVTAWIFGLQIAKEG

WFSAIMAPIFVASAMDSGLALLLIVLIALNAAKLFETPKKLIASLAGLLATCIAIDGFLI

FCEVITMAYPGAEGAATLAVMVSGPTAPFFWAEVVGGLLVPFLMLVFAKNRQNTALVTVA

SVLVVAGVLCKRLWLLLTAFVIPNIVGAPGIMSDAWMMGGSYAPTAIEFLIVLGVPSLGA

LAFMAIGSKLLVPATAKEHAPARSGAAADLDLEAQVA

>WP_160213011.1

MSDLIAAYLFCAGAGSGAAFLAAVFECFVRAGAFRRARFADRRQAVSMRAVALSVYGAAL

VLLAFGMLCLVFDLGRPDLALKLFLRPNLTLSTFGAFALAVLALALMVLVALRLGRENQG

AIRRRIDGLSRAVVIVASAAVMAYAGLLLGQADGMPLFETPWLAVLFVASALASGLAVVM

LAVAVAGNGHVEAVRYLKRRLTVRLDVALIVLEAVAAGLYLAAIALGPAGTVALAPLLTG

AQGGLFVGGFGLGGLTVPLVLDLLQWRRPLPGWAYGLAAVATLLGALALRFALVQAAGPL

LSWAPVA

>WP_160212856.1

MLDTFVTVYLFLGGCAAAVVLVTCAWSLAVRAACGRRQPAPPVFGRLRVRCLLAGFVLLV

LAVLCLLLDLGRPQLFWLLFARPTSSLISIGSFLLMATLLVSGFLLGASVPGAPRSSRRV

LCSAEVVCCALSAGVMLYTGLYMACLEAVPLWNNPALPVLFALSSLSSGLSVVLIAASFA

DDRFLLAADCRRLRLAHAVSLAGEMVAVGAYLALAWGDGFARPGLEALLSPNDLGSWFVV

GFLGLGVALPLGAEVFAAMARRPMEAIPLDALCIIGGLVLRFCVVIAA

>WP_160212813.1

MFDALVIAYLFLGGTGAALGGLLGLLTLGQVLGLGESGRGQHLNGLSSGQHRRFFGFGNV

LAAAVCLLGAVCLLFDMERPDKVLVLLTSPNTSLVAMGAYSLGAVLLLSALAGVLHLHRR

TLPPAAGALLCAAQLVAACVTMTYTALLLMGFRAVAFFQTWALVGLFFCSSASCGLALAT

LTGMVLRVFPLERGHERTAAAEAALSLLEGLFLVLFMIHAHYAAPQAFAQLATGPLAWAF

WTVVVGCGVASPVAAWALPRFVRGYRLHGGLSPALVLLAGFALRFCVIAVV

>WP_160212742.1

MDFNEGGREAASAAGRSAERAKEKAQAWGGAALNAAIGVSGVLAVLGIVLWGIQLSGGMV

QTAMRNLDSWGLYITMFMFFVGLSAGGLIISSVPKAFGIKGFGGISKVAVYSSIACTVAA

IGFVVVDLGQPMRLWELFVYSNLGSPLMWDIIVLGTYLILSCVYLWAQVQSEKGKVSAAA

LRVISVIALVCAVLVHSVTAWIFGLQVGREMWHTALLAPWFVSSALVCGTGLVMLVCMGL

SKAGYLEFSRENLVKLAKLLGAFVCVDLYFFGCDLLTEAFPAAGGMEVVTMLVSGPLAPF

FWVEIIGCILCAAVCFVPSLRKPGLLAVGAVLAIAGIFCKRVQLLVGGFQLTNLDMPGPV

TSLSVTNWESGFSGAYSGLVYWPTPLEFGVALGVVALAVFIFCLGVKFLPLRPKED

>WP_068921157.1

MSVQADSVVAHRWALRSGVYRATAANGDLMLAAWPHTAMLGHASPQLLALLDALAEGPVP

VDEPGMSATLDRLRAGGWLSRTVSCAGRDLYTVTPLAAPTEAPAPAGELRLSRFAVLRNT

PEGLVLEMPGSWCDIRVHDPAVAALLADPSGDAGLPADAAAAVRADLVAAGMLVAEEEER

EPFERRQWSTHELWFHERSRLGNRGWFGGAHFGGTFWARGVHEPPPARPSPYPGEAVPLA

RPDLATLRRTDPTLTTVLEDRESVRDHDDDAPITAEQLGEFLYRCARVRLLRTIEGFEYS

SKPYPGGGSAYELEVYPIVRLAADLTAGMYHYDAHDHLLRPVQPLGHPSVRRLLKVATES

SVTKAPPQVLLVISARVGRIMWKYEAMGYALMLKHVGVLQQTMYAVATAMGLAPCALGSG

DDLAFTGATDRDRLTECAVGEFMIGSRRKELATWQL

>WP_121705446.1

MKRKTALLVAACAALMALGGCQKVNEAPTTEAAPQAETGAATKKDPAGEKAEEGKGVNKV

AYITAQRLGDDGPVDMVYRGIKAGCDEAGIEVHVVEAKKGEYEESMQAMVSEGYNLIFAV

FPELIDSVKAVSQQNPDVSFIHAICATKGDNLEGICCYEQQSSFVMGVLAAMTTKNNKVA

FVGGVDNPDTHRYLDGYKEGIEYVNPEIEVQTSWIGSFEDPAKAKELALVHYQNGADVLW

GSGGKSALGLYEAAKEMGEGYYVMGCTDDNNGRLPGQVLASHYEAWDTAAKDLVIDWNDG

IFEPGLKVLTLENGYAYCKLADESQCEIPQEVRDKVEEVTEQIKSGEIVVKSMPTYEEVI

ATLE

>WP_000146146.1

MASGDLVRYVITVMLHEDTLTEINELNNYLTRDGFLLTMTDDDGNIHELGTNTFGLISTQ

SEEEIRELVSGLTHSATGKDPEITITTWEEWNSNRK

>WP_007792748.1

MSEVTRYVVTVKFHEKSLTDINELNNHLTRGGFQLTLADDDGKIHELGTNTFGLVSALSE

KEVAELAEGLGEAALDQKPQVTVTTFENWLRDNDTV

>WP_188061558.1

MRPVKKLAVTSAVAMLSLGMTACGSNSTNNNSSAPAGNSGASGSSGGSAAGALKVGLAYD

VGGRGDHSFNDSAAKGLDEAKAEFGIKPTEVAATNGENDAARVSRLQQLAQSGNQAIVAV

GFSYAAAIGKVAKQFPNVKFAIIDDASPDSKGDNIDQITFTEEQGSYLAGAAAALKSKSG

HIGFVGGVEVPLIKKFQAGYVAGAKKVNPNIKIDSTYLTQAPDFSGFADPAKGKTAAQGM

FQNGADIVYHAAGKSGDGVFDAAKAAGSGKWAIGVDSDQAQTAPAGVRPIILTSMLKGVD

VGVKSFLKKVHDGNFKGGNSVYALKDGGVSLATTGGHIDDIKAKLDELKKGIEDGSIKVP

SA

>WP_083706534.1

MTRAPVIGWTMLAGAALAASAGSLPIQLGFAILAIGILGMAHGASDLAIVAPGRRPLFLF

LYVSVSLICLAWWTGYPEIALPLFLAASAIHFGVEDAPHGSLPERAARGISLVATPAILH

REGYGDILAFAAGHGISTTVLFLLIAAGAVATALVLIMAIRRRDGRLLIGTGALLVLPPL

IGFSIGFLVLHALPQTDQRREEIGCVSHRAYFRAVAPILLAALLIAAAVGAFFVYREGTG

VRALFAGIAALAMPHLLVTPWFEGRAGRPVAYSCPAISGRAQHPQT

>WP_076714729.1

MDWMAFLIGFTIMSLASLAIYAKGSKTSPSLHHTLLHAAVPFIAATAYLAMAFGIGTLIN

IDGSVTYLARYADWSVTTPILLASLVLLAFHERGKMGEVGGYLTAIIVLDVLMIVTGLIS

SLALVPVLKWVWYLWSCAAFVGVLYLLWVPLRAMAAERGEALGTAYRKNVVFLTVIWFLY

PIVFLVGPEGLKIISDPTSVWAILIMDVLAKVVYAFYAAANLKTALHDHRA

>WP_056438944.1

MARLSIFPLAGAILFPGMPLPLHIFEPRYRALVSDAMARDRRIGMVQPSGEGDTPSLYQM

GCVGRIAEVEAMEDGRYNLVLEGVSLFRIVRELEVTTPFRQVEAELLPVIDEDLLSLGRR

ASLEQESKRFADLQGYAVDWDAVGRLDDESLVNGIAQIAPFDVAAKQALLEAPDLEQRAE

LIIQLMQFFGRHDGEDRVTLQ

>WP_056438392.1

MRIDLTPYRRSTIGFDRLFDLLEANSRAASAENYPPFNLERLADDRYRITLAVAGFARDE

IEITAQQNMLLVTGKKDDKAGSPNFLHVGIANRSFERRFELADFVFVEDARLNDGLLVID

LVREVPEAMKPKTIAIKTGQPLAAVEHHAGEADEAKAA

>WP_162547883.1

MKNILLALAASAAAIVGVAAPAAAQDKTKVCFVHVGSKTDGGWTQAHDIGRQQLQEHFGD

KIETPYLENVPEGPDAERAIERMARSGCALVFTTSFGFMDATLKVAEKFPDVKFEHATGY

KTAANVATYNSRFYEGRFINGQIAGKMSKTGVAGYIASFPIPEVVAGINAFLHGARTVNP

EFKLKVIWVNTWFDPGKEADAAKALFDQGVDVLTQHTDTTAPMQVAEERGLKAFGQASDM

IAAGPTAQLSAIVDTWAPYYIKRTQAVIDGTWSSAQTFDGLKDGILSMAPYTNMPDDVKA

MAMDTEAKIKSGELKPFSGPINKQDGTPWLKEGESADDGTILGMNFYIEGVDDKLPQ

>WP_015068113.1

MTEAVKTPYPRTFSHIGISVPDLEAAVKFYTEVLGWYLIMKPTEIVEDDSAIGEMCTDVF

GAGWGKFRIAHLSTGDRVGVEIFEFSNQENPENNFEYWKTGIFHFCVQDPDVEGLAEKIV

AAGGKKRMKAPRYYYPGEKPYRMIYMEDPFGNILEIYSHSYELHYASGAYE

>WP_169634528.1

MTNLQKRSLLQTVALTAVAVAALVGCGKKEEVAPAAAPGAEAPAKSEPLKIAFMYVSPVG

DGGWTYQHELGRRAIQEKFGDRIETSFVESVPESADSERVMRDMAGQGSKLVFATSFGYQ

EFVQKAAADLKDVKFEHATGYKTAGNVATYDTKTFEGAYLAGIVAGGMTKTKTIGVVASV

PIPEVVRNINSFVLGAQSVDPAIKAKVVWVNEWFSPPKESEAATSLINGGVDVMYQNTNS

PAVLKTAQERGVRAFGKDGDMSAFAPQAHLGSAVIDWTPYYTKVTQDTLDGKWEGGSFWW

GVKEGAMDLVKIADDVPQEIKDRVAKAKAGMKDESFHVWTGPIQDNAGKEVLPAGKVGDN

AFLTGIDFYVHGVEGKVPGAK

>WP_159120456.1

MEHYLSLFVRSIFVENMALSLFLGMCTFLAVSKKVKTAMGLGVAVIVVLGISVPVNQIIY

VNILAPGALAWAGFPEADLSFLNFLTFIGVIAALVQILEMSLDKFFPALYNALGIFLPLI

TVNCAIFGGVAFAVQREYNLTESVVYGVGSGMGWAIAIVLLAAVREKLKYADMPDGVRGL

GSVFMIAGLMALGFQSFTGIQL

>WP_159120259.1

MDLATVIGMLGAIGFIVMAMILGGSLSMFIDVQSILIVFGGTLFVILSQFTLGQFFGAGK

IAGKAFMFKIESPEELIEKIVEMADAARKGGFLALEEAEISNEFMQKGVDMLVDGHDIEV

VRETLSKDISMTSERHDFGASFFKGMGDIAPAMGMIGTLIGLVAMLSNMDDPKAIGPAMA

VALLTTLYGAFFANVICLPIAFKLSVRAGEEKLNQSLVLDGIVGIADGQNPRVIEGVLKN

YLAASKRGSAEEE

>WP_159120252.1

MSLERFPLFPLSAHLLPEGRMALRIFEPRYVRMVKQACAENSGFVMCMLNSNGDKETNKH

IHKIGTYAQVVDFDMLDDGLLGIKVAGSHLVEVSNIEAEKDGLRTGDCKTIPQWQCDLAP

QQIAPMDERLKEIFGSYEELAALYESPKFDNPNWVLNRWLELLPVDGSQKQHFLAQRECT

SLLNYLSGLIG

>WP_105932012.1

MRTIDLSPLYRSFIGSDHLASLIDAASRAEKQSTYPPYNIELLGDDKYRVTMAIAGFSKD

DVSIQVEENTLTITGTKKAETEDKESKERKFLHKGISERNFERKFQLGDHVKVLAADMEN

GLLHIDMERVIPEAKKPRQIEIGSRLLENQ

>WP_105930623.1

MTQEETQIKSIPAKAYSVLEEWMNSITHGLGLIAAIIGLVFMVYRADNPLALTTAVIYGS

TLILMFLSSTLYHAISHDKAKGWLKLFDHSAIYLLIAGTYTPLLLVSIGGVLGITMTAVI

WCLAIGGVAFKLVAQHRFPKVSVMTYLLMGWIALGLIYPLYLALPGAGLWLLVAGGLCFS

LGVCFYVAKKVKYTHAIWHLFVIGGCSCHYFSIYYFVF

>WP_018697113.1

MESLNIFIRSIFIDNMVFAFFFGMCSYIAVSKSVKTALGLGAAVTFVMVMTVPLNYLLYE

FVLKAGALSWAGLPDVNLDFLTFIVFIATIAAFVQLVEMAVEKFSPTLYSQLGIFLPLIA

VNCAIMGGSLFMQQKVDALELTSLWQSIVYGLGSGLGWWLAIVMMAAIREKTTYSQIPAA

LKGPGIAFIITGLMGIAFMIFSGIQF

>WP_005856736.1

MFILMPVIFVLGILAIALEDKIKINKAAIALFMAISMWMILMFDAYNIFVERSSTIFQEF

LTQNPEMASLPPHEQFINFISNRAIVYHLGNVSETLFFVMCSMLIVDIVDKHGGFRAVTG

YIRTPNKRKLLWYISFATFFFSALLDNLAAAIVIMAVLRKLVPDRTDRLKYACMVIIAAN

AGGSWSPIGDVTTILLWVGKNISAMHQISHVFIPALVNMLVPLTIAHFWLFKKGSTLRVL

SEEEQGDEYIPEIPNRSRRMIFVIGVLSLALVPVFQMVTNLPPFLGVLLGLVILWFYTDL

MYSKLHMHESQKLRISQLLPNIDLATIFFFLGILMAVGALETSGQLGIMSAFLDKHVHEP

YLISFVIGALSSCVDNVALVAATMGMYPIVEQVADLSPYAQFFVSDGGFWTFLAYCAVTG

GSILIIGSATGVTVMGLEKIDFMYYTKRFSILALIGYCCGAGVYMLLFA

>WP_169169348.1

MNALVGRGGLFDEFFKDVNPGFYVRPLHGDPLPTPGQMKVDVKENDSGYTVCAEVPGVPK

EDIQVSVEGNVVSLRAEVRQQDQQTEGEKVLRSERYFGAVARSFQLPADIDAAQCKAKYD

NGVLTLTLPKKQGGNAQRLSIE

>WP_159490586.1

MTEVKQQTKTISAKAYSVLEEWLNSITHGIGCIAAIVGLIFMLYRAEDKLALTTAAIYGS

TLILVFLSSTLYHAISHQKAKGWLKLFDHSAIYLLIAGTYTPLLLVSIGGVLGITMTAII

WSLAMGGVAFKLIAQHRFPKVSVMTYLLMGWIALGLIYPLYLALPGAGLWLLVAGGLCFS

IGVCFYVAKKVKYTHAIWHLFVIGGCSCHYFSIYYYVV

>WP_142437480.1

MIKKISRYEMLNEILNCVTHGLGFILSIIALIALTTKAANLKSSIHVIAYLIFGIAQVLL

FFSSTIYHSLMFTKFKRVFQIIDHSSIYLLIAGSYTPYCLLAIGGTFGWGLYSFIWTCAI

AGIVYKNITMSKENKIPKYSMITYVLMGVFAILIIEPLYKSIGLTGVLLLVSGGLFYFLG

TYFYRSKNMNFSHPIWHIFVILGATYIYFSIFLTT

>WP_131622846.1

MNTLLIIILVVLMGLVVVSLVRGIVAFLQSHKADIDAGGQRQQDMQLLQNKMMFNRIKYQ

ALAIVVVAIIISIAR

>WP_121708026.1

MVGNKENKKRMEGWDITSLEAEGLHKCIRPGVQDCRAVSVYRLNLKQGSRFTLESGELEM

NPVLIRGRAKLSGAGLDGELEKLDSFYIPGDTGVGLEALEDCVFYIGAAPCEGYGKPFVR

KFDLSLPLGDIHQIHGHGVGQREVFFTLNHQVEASRLICGLTWGANGAWTSWPPHQHEKD

LEEVYCYFDMDAPRFGFHISYLKSGEVEDIVAHTVRSGSMVLAPAGYHPTVASPGTRNTY

FWILAAHSHASRRYDLAVLDPVYADT

>WP_121707743.1

MMFKVKDPGSALTHFIAMLLALAAATPLLVKAARSPEQTHILALTIFIISMVLLYAASTV

YHTLDISPKVNQILRKADHMMIFILIAGTYTPVCMLVLGDYTGWMLLALVWGIAFFGILI

NALWITCPKWFSSLIYIAMGWVCILAFGKIIAALPASAFGWLLAGGIIYTIGGVIYALKL

PLFNSRFKNFGSHEIFHLFVMGGSLCHYIMMYAFVA

>WP_106897292.1

MGTYMREPINGLTHLFGAILSFVGLLAMVIKASTTADSTLTIISVIIFGISMTLLYAASA

TYHLVVAKAHVIAFLRRLDHSMIFVLIAGTYTPLCLISLNGMTGWVLFTIISAIAVAGVS

FKLIWFHAPRWLSTALYIAMGWIVVFFSSSLAPVLGTNGMALLIIGGLIYTVGAFIYWLK

PKFMNFKHFGFHEIFHIFILLGSLFHFLCVYLYVL

>WP_016292657.1

MQITIREPGSAITHFIGMMMAIIATAPLLVKAAMEPGAASLASLAVFMLSMILLYGASAT

YHSVNFSERAIKIFRKIDHMMIFVLIAGSYTPVCMITLGGKLGYTLLAVVWGIAILGMSI

KALWITCPKWFSSIIYIAMGWVCVAVFGPLWRTLPASAFLWLLTGGIIYTIGGIIYALKL

PLFNSQHTHFGSHEIFHLFVMGGSICHFIFMYLYVA

>WP_032850333.1

MENKRYNNVEEWANTLSHGAGILLGVIAGYFLLAKAAAGAEPKWAVACVTVYLFGMLSSY

VSSTWYHGSRPGKLKELLRKFDHGAIYLHIAGTYTPFTLLVMRHAGGWGWGIFSFVWLSA

IVGFILSFKKLKEHSNLETACYIAMGACILVAMKPLMDHLAEMGAGPAFWWLIGGGVSYI

IGAVFYSLRKPYMHATFHLFCLGGSIGHIIAIWLIL

>WP_121698083.1

MKHKAIFIRDDNGTEVSAQSPVIVSASRGTDIPAFYADWFFRRLEKGYVRWRNPFSGQDS

YVSFENTRFIVFWSKNPAPLLPYLPMLKERGIGCYIHFTLNDYEAEGLEQNVPPLSQRIE

TFRRAVEALGRGAVVWRFDPLILTDKINIDTLLEKIAHIANALTGYTEKLVFSFADIESY

KKVSRNLRQSCINYREWDEESMCEFASRLSTKNHDNWNLRLATCAERIDLSEYGIGHNRC

IDPELISRLTPHDAILQNFLYNAKTDNGQRKACGCILSKDIGAYNTCPHGCLYCYANTSS

ASAFANYKEFATNPLTDLII

>WP_149888968.1

MSLKLVAAVAAVASAFALSACGDKKEAAPAKPATPAAPAAQTEAAEPLKVGFVYVAPIAD

VGYTKQHDIGRIYAIDKVGKDKVTTTFVENVPETADAERVIRQMVADGNKLIFGTSFGYM

NYMQKLAKEYPDVKFEHATGYKTAPNMTNYNIRFYEGRYLAGMLAGGATKSNIIGYVAPF

PIPEVLQGINAFTLGAKSVNPNIQVKVIWTNAWYDPPKDTDSAKTLLGQGADILTQHTNT

SAVASAAEAAGKMVIPYNSDMKSVAPNAQIAALVLNWGPYYAKKIQQTIDGKWDPTPVWM

HYKDGAMSLEGVRTDKIPADIVKKMEEVKAKIESGEFHPFTGPIKTNDGKEAAKAGEVLK

DNQLQTMNYYVDGVIGKVPN

>WP_010714134.1

MTKNKLFHLTDKSNINSIRKHGLVGVKKAKDLLRRETGTDHTLFNQQTYSILNTYGWKGY

DLRCATFMFEEDSCLGYELLQLMNDDPYILEIEIDRLNKDKLFVFNTEIASHLLNYSKQD

QHRLAKFYWNTAIPYNTYVQNKDKVNDAFQLGNIMYQAEYVYFGEISPKYIKNYERGKKY

DSGRY

>WP_007273123.1

MLMRTDPFRDLDRIAQQVFGTPARPAAMSMDAWRNGDTFEVEFDLPGIKPDSIDLDVERN

VVTVKAERPALDKDLEMLASERPRGVFSRQLVLGENLDTENIEASYEAGVLRLRIPVAEK

AKPRKISITAPSQDREAIDP

>WP_006681612.1

MREVVEYLEDRGVEHLVHFTPITNLGGIKKRGILPRNEIDGFPDIVFEALDEVRLDERTD

MSCFSISFPNFLMMYRYRTKLWSREEDVALLFIPISVLSDLEYDQVVFCPSNAASRECRR

TDPQDLLGLAAVEKLFVEEMTTRSGVVFSRQSEDLPDFLTTNPQAEIQIAATIPWEKVSF

VVVNDYETKQSLLQSGIHRNVYAKWEVNNDSSLNVFKYPSYWRAWVDAAGDLHG

>WP_121699091.1

MENLNIFIRSIFVDNMIFAYFLGMCSFLAVSKNVKTALGLGAAVTFMLVISLPINYLLET

YVLRAGALQWLGPEYADVDLSFLSLIMFIAVIASLTQLVEMAVEKFSPSLYSSLGIFLPL

IAVNCAILGGSLFMQQRDFPDVWTACCAGAGWGLGWLLAIVAIAAIRERLQEYSNIPKPL

RGVGITFILTGLMGIAFMSFLGIKL

>WP_121699089.1

MNKQSNTYTIIYIVALVIIVGTALAFTALSLKPLQTANADADKMKQILASVHIAPAKSDI

ITDFDKYITDRFVIDAEGKRVEGDAFAINVSAQSKLPQAERKLPVYECTLTPGDVKYILP

VYGAGLWGPIWGYVAVNSDGSSIYGAYFAHQGETPGLGAEIEKPAFSDQFTGLNLFKEGA

FKPVNVIKAGQAPMNGEDYVDAISGGTITSKGVASMLDNCLSGYKTFLESLTNKGQ

>WP_167508517.1

MSGSSRLAKFRTAQADKQNWQCFYCGFPMWEGDLALPSEHRRLPIGLLDRFLCTAEHLEP

KMNGGKNRPDNLVAACRFCNQTRHKMRDVLSPAAYQQHVRRRIRARKWHPIECHRLFG

>WP_160582050.1

MPYKPKRPCSYPGCPKLTDGRYCGEHQKIVTAHYNKHERDPASKKRYGRAWKRIRDRYIA

AHPLCEECRKAGKVTPAEEVHHIRPLSKGGTHAEGNLMALCKPCHSEITAREGGRWERRR

>WP_135855810.1

MIEFIDDTEPWRPTTSGERLLFLLLANHKEARFLSEFLAGSGLRFARRHSDWSNFLVGAS

LLHLQQRLIKLGNDVREKSPSDRMDQLKELRARTCDIVQLTPIEYEGDFGELVQEVLVSA

EQMHKEPPQNLKRSVLRSSPSCYSCGRNFGSVYENDEDAKEGLRATADHVWPRALGGDST

EDNLLPACTSCNSTKGHLATWHMAWLQPIVFSDVDGEHAPPPVPREVQMALHMRAATSYA

RANGTTLRDAFLAIGPRDRPEKIDSEQGYDFFNMRVHNETRTMVKWIPG

>WP_120438463.1

MPGWNLKNGELQKCQISEDEYWSLFNFVFSDACMKRNTYKFGLIKSIMDNLFNCTQDDYG

NYRLSYSAIFEKFTINYWNLVLKYHLKQMRSDGRTEVSKIESILLAASEENDLIKTLDFN

SLSNSDRSKVVRQVSIACRKNVIGALYNDMEGKLYGFDLKEKGIMLGERAYDFMLKYKTE

LEKLNYYAWAKFMEKINEDDVLVKVLDKLELSTPKRDDLSVYREVLYEEFEACNCFYCGK

KLSLSNRGIHVDHFIPWSYVKDDKLWNFVLSCPKCNERKNNKIPSKKYLEIMLKRNEYMK

GVVDEFVEIEFKNYDSSQFMRLWKYAQLSGMKQFQQEFF

>WP_120438225.1

MESYILHVRSVKESMSREVINTYQLEEILPKIIDYEEENYYNNIGVDSNLEKDLLEDLQM

LDNKYKEITKSVIIRYQKIVEHIKQTRGRKCQICQYSFIMDNGNEYCEAHHIQYLSKNGS

QSSDNVILLCPNHHRMFHYAHDAVFVDDLVDGKRKVLIENVEHLIDFS

>WP_140970856.1

MKEYKTKQQKRKFYDSGEWKSIREQVKKRDNYECQECKRNGRVQTDTNEYSESAKRKKIQ

LVVHHIKELEYHPELALEKDNLETACVDCHNKEHGRFFEKKPNKWENDEKW

>WP_169253183.1

MKTLVLNAGYEPLSIVPFTRAVVLVLTGKATVLAAEDIPVRSEHMSLDQPSVILLTRYVR

PPSNRRVSLSRRGVLRRDGHRCAYCSKPAYTVDHVLPRSRGGANTWENLVACCRECNNRK

GNRTLGEIGWKLSFLPQEPRLGQLWMRGIDKPVEKWRPFLEYSSAA

>WP_083793542.1

MMRLFNKRQRRILAWVAGGQCTICQRPLNSNFHADHVLAHSKGGATTTDNGQALCAPCNL

KKGAK

>WP_040799872.1

MAVAQTRRARAARRRKRRVDAADNDLTAEQWKELKREWGGCAYCTATDTVLQKDCVQPIS

RGGSYTVGNVVPACGSCNASKSNSEVTSWMRRKRLDERAFLTQYVHVRQALGLV

>WP_007230052.1

MVELEIEVGDEIKNEDLVRMFGCGPQGGMRRSHATNTLVLTSKHVDNVYDDRWVADVFHY

TGMGLEGDQSLTYSQNKTLAQSASNGVEVHLFEVFKPKFYTYMGAVELAAEPYVENQKDQ

NGLERKVYVFPLRPISGGQPTLPFKKIESAIHNKQKMAHKLSDQELLNRAASKSSAGASR

SVETKYYERDPWISEYAKRRAGGKCQLCESDAPFISKAGEPYLETHHIEWLANGGEDSIS

NTVALCPNCHRKMHNIADNNDVVKLKSRNASYE

>WP_007225374.1

MSTFLLTWSPDKWGYENLQEYLDARKSEEFVQRWSSGRTKKIPIGSRVFLTKQGKGNKGI

FGSGHVTKEPAEEPHFNEEQLKLGKKALFVMVNFDQLYDPQSEIPITHSELQAFDSKVWD

SQSSGITIPEETASKLEQLWLERTGAVEISYADEVPKDNSLKEGAAKKIWVNAYERNPDA

RERCIRKWGLNCVVCNFHFEQCYGHLGKRYIHVHHLKPLAEIQKEYEVNPEEDLRPVCPN

CHSMLHRNKNSVLSIEELQTLVNMYSR

>WP_169253656.1

MSTSEYDSYRPPEPEGGRKKRRRGGRGGPSGPDGPRGRRGGRGGGWKNRGADGNREMPMV

EDVEFTSYYGRPIVKAPPWGDEISAYLFLGGLAGGSSLLGYGAQLTDRPGLRIASRMTAI

AATGIGGVALVADLGRPERFLNMMRVVKVSSPMSLGVWILSGFGVGSGVTFAIELDRITG

EKLLPLGPLRKVLHGLETPAAVESAFFATPLAAYTAVLLGATAVPTWNAAGRNGLPYVFV

SSASMAAGGAAMALAPVGQTGPARLLALAGTAGEAYAMSAMKKRMHPAEVDPMDDGEPGH

KLHRAEKLLIAGTIGTAVAEVGARVFAKKLGGGWKTRAVLRGLSVVSGAALAAASAYTRF

GVLEAGIESTKDPRHVVEPQRARLEERRARGITDDSITTGR

>WP_141265030.1

MLVKALTGSKRYWGWITLLLVLIGTGFTCYLWQLDKGLTITGMSRDVSWGLYISQFTFLV

GVAASAVMVVLPYYLHNVKAFGRITILGEFLAVAALIMCLLFVLVDVGKPMRILNMIFYP

TPNSMFFWDMIALNGYLLLNIIAGWHALEAEYKAVPPPAWTKVLVYISIPWAVSIHTVTA

FLYAGLPGRHYWLTAIMAARFLASAFASGPALLILLCYIIKRVSKFDPGREAIQKLAAIV

TYATIVSTFFIGLEFFTAFYSQVPAHGIYTLKYLFAGLDGHSRLVSWMWAFAILVVFALV

LLINPGTRTRDSYLQLACAAVFVSMWIEKGIGLVIGGFVPNPFERVTEYVPTLPEILIAL

GVWATGFLVLTFLYKIAISVKEETV

>WP_160582917.1

MKLKIKDPGSALTHFIGMVLAILAATPLLVRAAHTPGPLHIAALAVFICSMILLYTASTV

YHTFDISESVNRLLRKIDHMMIFILIAGTYTPVCLIVLGNPAGYRLLALVWGIAVLGILI

NALWINCPKWFSSCVYIAMGWVCVTAFREIVAALSPAAFGWLLTGGIIYTIGGVIYALKL

PIFNSRHKNFGSHEIFHLFVMGGSFCHYMMMYGYIAA

>WP_160582365.1

MLMPSIFGEDLFDEMMGFPFDNRFFARRNPVYGKAATAVMKTDVKDMDGMYEISMDLPGF

SKENINAELKDGYLTVNATTSVNQDDSSEGRYIRRERYCGSMSRSFYVGDAVKKEDIKAR

FENGILALTIPKVPEQPKVETPNYIAIEG

>WP_160581716.1

MIISASRRTDLPACYPDWLFQRLKEEYVLVRNPMNAHQISRIDLSPKVVDGIVLWTKNPL

PLFRHLNELEKYSYYVQFTLTPYGPEAEPGLPSKNRVMIPAFCRLSREIGRERVVWRYDP

IFLSNVYTMEYHTKYFRVLASRLGEYTEKCTVSFLDLYQSTARNGRPLGIHTETGEQQLE

LMERFAEIAEEWGITIDTCAEQGDFGRFHVGRASCIDKDRLERIRGCQLKVKKDPNQRPE

CGCAASIDIGTYDTCRIGCLYCYANHHRDTVFKNSQRHNPASPLLFGEIGENDRIMERRM

ESCMDGQMSFHDWHG

>WP_135902375.1

MRSPEYAPLFRSTVGFDRLFDMLENSVRTDWPPYDIEKKGENEYRITMAVAGFSQEDVEL

TQHGPELTVTGQKSTAENGVQFLHRGLASRNFKQVFRLADHVKVANATLENGLLSIELVR

EIPEELKPRRISITSTATADPQPQISQDVKPGRKVA

>WP_135898580.1

MRHVDFSPLYRSTVGFDRLFTMLDSLAQPDGAQTYPPYNIERTGEDSYRISMAVAGFSDD

EISIEAHRNVLTVKGERKDEGTGEGSELLYRGIASRAFERRFQLADHVDVVGAALKNGLL

FVDLKRNIPEELKPRKIAINSAPAKAKQIEAKTAA

>WP_135873972.1

MPAQTDAAADWSGWRLAHAMAARFVRWAVDTEGSSRSSALIRIGLAMLFWSRWAGELLLY

MDQSPAGLFLAANFFVATTLLFVGYQSRVAAVWTGAVGLAMYHYFGFQLGREPWTHHHTY

LLAVSALLIALTPCGASYSLDRYLAVMRAERMGLPPPAERGNLLGLRLIVVQLSVLYFFA

AFDKSGYAFSSGARIEAIFLWYYAGSDYPAIPGLAWLATIVSLAVVALEYSLAFGLPFRA

TRRYLLLPGLAFHAIIYVTLPVYTFSATMVLLYLAYFDADAVDRVIARLQGIGPTAGEET

S

>WP_120424976.1

MTLKIKDPGSALTHFIGMLLALFAATPLLIKAARSPEQTHVLALTIFIISMILLYAASTT

YHTLDISPKVNQILRKVDHMMIFILIAGTYTPVCMLVLGDRTGWALLGLVWGIALAGITI

NALWITCPKWFSSLIYIAMGWVCVLAFGKITAALPKSAFGWLLAGGIIYTIGGIIYALKL

PLFNSRYKHFGSHEIFHLFVMGGSLCHYIMMYAFVA

>WP_135901799.1

MDDVPGNAARYRTGLAVRRSVLGDPHVDRAEIAATDFDQPFQELITEAAWGTVWARPGFS

KRERSIVTLALLAALGHDEEVAMHVRATANTGASRSDICEAFLHVAIYAGVPAANRAFKI

AKEVFSEMDGGKAVHAR

>WP_135901798.1

MPFVTLGGITLHHRYVEANGKTPAVVFINSLGTDFRIWDQLLSELDGEMPLLVYDKRGHG

LSDIGDIRSIDDHVDDLIGLIDHFGLDRLVLCGLSVGGMIAQGLYARRPEIVAAMILCDT

AHKIGTAESWNARIATVQANGIRAVADAVLKVWFTPPFHSERQPELDGYWNMLTRQALPG

YIGTCMAVRDADFTETARRIAVPTLCVVGDQDGSTPPDLVRSLADLIPGARFEIISDAGH

IPCVEHPAALVALIRDFVASLPSGETHG

>WP_135895629.1

MQRSLADSRVKFRHLQCFLAVAQFGSVQRAAGSLSITQPAVSKTVAELEDILGVKLFERG

RHGAVPTREGQLFMPHASACVSALRQGVDLLARAEGAAAATLEVGVLPTVAGALIPPVLK

RFASLWPRVIVRLATGANPELLERLKAGTIEFAIGRLADPERMVGLSFEQLFSEPLVAVV

RAGHPLDVTSGLPPAALQDFPVVLPPFGTLIRQSADSLLSAWGVLPLSAFVEVLSVSTGR

ALTLENDAVWFVPLSAVEYELTHGMFVRLPLPFAGTDEPVGLIRRSDTQPSPVGRAFIDA

VREVAQARMAASGGKAAGKVARKRGRGRTPAS

>WP_135891186.1

MPYAAVNGTELHYRIDGGRHGNAPWVILSNSLGSDLSMWTPQVAALSKHFRVLRYDTRGH

GHSEAPKGPYTIEHLAGDVLGLMDTLKIARAHFCGVSMGGLTGVALAARHASRFERVVLA

NTAARIGSPEVWVPRAARARTEGMLALADAVLPRWFTADYIEREPVVLAMVRDVFVHTDK

EGYALNGEAIDATDLRPETHGIKLPVLVISGTHDVAATPAQGRELAQAIPGARYVELDAS

HISNIEKADAFTKTVIDFLTESK

>WP_135872108.1

MAESRIRFRHLQAFLEVARQGSVARAADFLHVSAPAVTKTLRELEEALGVPVVERDGRGI

RVTRLGEIFLGHAGSAISALKRGVDSVRQDGALNRDPIRIGALPTVSARVMPLAMTLFLE

ENTGAALKIVTGENAVLLEQLRVGALDLVVGRLAAPEHMTGFFFEHLYSEQVLFVVRAGH

PLAEPGTDIFARLDEFPVLMPTRESVIRPFVDRLFITNGMTAPATEIETVSDSFGRSFMR

QSNAVWIISAGVVANEIASGAFVALPVDTEETKGPVGLTMRTDTAPSPAFTILLKTIREA

ARHHA

>WP_135855689.1

MHFLRCGETVIHYRVKGLDSGKPVIAFINSLGTDFRIWDAVTEVLGDDYAYVLHDKRGHG

LSDIGRPPYSIDDHAGDLIALLDHLGVKSAVIWGLSVGGLIAQGLYARRPDLVRALVLSN

TAHKIGTADMWNARIDKISADGLGSLVDPVMERWFTPAFRTPDNAAYAGARNMLAQQPEA

GYSGTCAAIRDADFTAAAGRIAVPTLCVVGDQDGSTPPELVKSLADLIPASRFVTIAGCG

HIPCLEQPLAYAQAACIFLKTLPEN

>WP_120446043.1

MAKGSAEKRRNMKKLLEEMEKNQELKAKIKELDENPKSTTKDYIQVAAENGLELTEADFQ

PAGSVGELADDELEAVAGGKDACTCVVGGGGQAYDEKTCVCVLGGGGEFTDGEARCICVA

IGTGQHG

>WP_120446041.1

MKRLLEEMEKNQELKAKIEELDKNPESTPKDYIRVAAEYGIEIKEEDFKPAQGELTDDEL

DAVAGGEPCACVFGGGGTANQSDDTCACVFGGGGEYSDGSCRCACVGGGAGDGHHYVPVS

DLFR

>WP_169251451.1

MDLTASVLAAPAAPADDARVLVVLPSLGTSAAALWQQAATELTALSPETTVIGIDLPGHG

RSAPIDVPAGTSVAPRITMSDLAEAVLATLDRVLPDVAAPGAPVDLAGDSIGGATALQLA

LDHGDRFGRIAVFCTGAKIGEATAWEERAQTVATSGTPTQVIGSAQRWFGEGFMDREPDA

SAALLHSLQDADRFSYAALCYALADFDVRARLPEITRPLLAVAGSQDQPTPATKLAEIAG

DVPGALLEVIDGAAHLVPAEAPVVTAGLLADFLQGKGLGAGAGGTGGAGATAASDSPADL

PTASGPREASRDEVREAGMTVRRQVLSDAHVDRANAKVDDFTSDFQDLITRYAWGEIWTR

PGLERRMRSAITLTAMIAGGHEAELAMHVKAALRNGLTRDEIKEVLLQSAIYCSVPSANT

AFSVASRALAEYEAEENAD

>WP_007228512.1

MKRTLLLLLAISIAACATGTDPAVTASAIDDFQPDARPTAKTMVYECDDAEFITRVGPGE

MALWFEDRYLILSQVRAASGTKYQEGDVVFWSKGDQVIFSAAGVRYANCQINHVRAPWED

ARRRGVDFRAVGNEPGWHLEVRGDQHLLFVGDYGATKIMFSNVHTTEDREQLHYLSQDDD

NSINVTVIESACIDTMKGDQFPYNVQVQLNDRNYQGCGRTLDHPWE

>WP_086005900.1

MSARLARFVKDPKHRLELLQDIHAASAQADLPPELVLSLIEVESHFDRFAISRVGAQGMM

QVMPFWKNEIGRPDDNLTLNKTNFAYGCRILQFYLQREKGDLHKGLARYNGSVGRRVYSD

KVYRAWNDHWRTEPLDWGD

>WP_040812715.1

MKQRVVLIEDELALAASYQDFLQRAGYDVTIYRTAESAVKGVEDCHPDLILLDIGLGHDP

EAGFELCRTLRARDALVPIVFLTARDEEVDVISGLRLGADDYLTKDISRSHLLARISGLL

RRVVALRNPENQEQVLKRGDLELNSERLTTTWGKSQIALTYTEFWMLYVMAKNPGHIKSR

EQLMEAARVVLDDSTVTSHIRRIRRKFETADKQFKHLETAYGLGYRWRGDA

>WP_007226915.1

MKNIQRSIGKSITALIMVLLGSSMIYQAGALMLEDTVDGKDNLYSSEWGHWFTMPGDGAL

AAYAPQSTAASAIVDSSNNAYDFSSWDYLDIVVTGSVTDAGSYETDAGGCTDPTASCWFG

DGQFRYQDVYSVIGIWSSSADEISWLDTIYDNWVDAVFTVGSDASIEVPEIEGAYLFLAE

NDGFFADNSGFYTATITTSVPEPASALLMLTALLGLFAIRRQRRAL

>WP_078486412.1

MRINIKLAAGFPLSRLESTFHGIETTRDENNTHTVRLTKSEVTADRDFELTWSPVPGNEP

HAALFSEQWAGDNYSLMMVIPPHQEGQGGALPREMVFVVDTSGSMHGASMGQAKAALKMA

LSRLAPDDRFNIIQFNSSTQALFGRAVGASPRNLARAEDYVDSLTASGGTEMLPALRRAL

TGEKELDRLRQVVFMTDGSVGNEAQLFEVIEQKLGASRLFTVGIGSAPNSFFMTRAARLG

RGSFTYIGKVSEVRSKMKALFNKLESPVLADVEIDWGEDVQVDMWPRRIPDLYMGEPLVL

AVKGEVDGKTVVIRGRSGDKPWQQRVTLHGGGSRGGIRLLWARKKIADLMDQKARGRTED

EVRHEVLVVALGHKLVSKYTSLVAVDKTPSRPLDQALIGKNVPVQLPKGWSAEKVFGSMP

QTATPALLNLLLGVLAMIGSWMVSVFGKRNRKNVADEVRHLNGEMYR

>WP_007229091.1

MQQHIAIVEDEAAIAANYRDHLQRQGFRVSLFADRDSAADAFAIQLPDLAIIDVGLGKEM

EGGFELCRDLRARAPGIPIVFLTARDSELDIISGFRLGADDYLTKGISQAQLTARINALF

RRVKALQKPEQEKHLVVQGALELNKERMTANWRGLPLELSVTEFWMVHTLALHPGHVKNR

QQLMDCANVVLDDNTITSHIKRIRRKFQALDADFGSIDTAYGVGYRWKG

>WP_082785960.1

ANIEVRLAKGVDKSTIASPYHQIKLDEPHHGIINVSLTNSVVANRDFVLQWRAKQGMSPM

ALVFNQQGKTHGDGASEDNVSENRQSDDHYSLVMVLPPKTDEHALSTLPRELILVIDTSG

SMAGDSIVQAKSALLYALNGLKAEDSFNIIEFNSELTQLSPTSLPANQTHLARARQFIHR

LQADGGTEMALALNAALPRGINRLSESSQSLRQVIFMTDGSVGNEQALFDLIRYQIGESR

LFTVGIGSAPNSHFMQRAAELGRGTFTYIGNVDEVEQKISLLLSKIQYPVLTDINVRFDD

GGVPDYWPSPIPDLYRGEPVVVSLKRSEREPQELVISGRQGHKNWQQSLSLKDSHGGAIT

EPDAGLDLLWARKQIAALELSKNGANDDKVKQQVTALSMNYHLVSPYTSLVAVDLTPIDS

SAMTRDAVVRQHLPLGWKPFGVLPQTATSSRFDMLLGAVTLILALLLAGSMLRQRRKERA

VILAIPYKQTL

>WP_007229132.1

MPDLMARALLPLLILLALQQLGSAGLIKAKAGLAPLMLAKAWEQSLASQGRPVKPWPWAD

TWPVAKLQVPSMGISQFVLAGDTGNALAFGPGHNLASAALGAAGPAMIGGHRDTHFQFLQ

HLRKGQRIVLQLPDGLLRHYRVKQMTVDTASGDMLWPNVGEQLLLVTCYPFDAFVTGGSE

RFVVTAEPESLPLQTLDALGGEPQRILL

>WP_007230524.1

MEDNIFQLQYALDTFYFLICGALVMWMAAGFAMLEAGLVRAKNTTEILLKNVALYAVSCT

MYMICGYMIMYGGDLFLSSITGDGVAGAEEAATYAPSADFFFQVVFVATAMSIVSGAVAE

RMKLWAFLAFAVVMTGFIYPMEGSWTWGGNAVFGMYTLGDLGFSDFAGSGIVHMAGAAAA

LAGVILLGARKGKYGPQGQINAIPGANLPLATLGTFILWMGWFGFNGGSVLATASVESAN

SVAVVFMNTNAAAAGGLIAALLVAKIMFGKADLTMALNGALAGLVAITAEPSTPTALQST

LFGGIGGALVVFSIVTLDKLKIDDPVGAISVHGVVGLLGLLLVPLTNDGSSFSGQLIGAV

TIFGWVFVTSLIVWGVLKAVMGIRVSEEEEYEGVDLAECGMEAYPEFTTK

>WP_007224114.1

MENEIFQLQYALDTFYFLICGALVMWMAAGFSMLEAGLVRSKNTTEILTKNVALYSISCI

MYMVVGYSIMYGGGDLTFFLDGIVGDGVTGAEEPATYAPSADFFFQVVFVATAMSIVSGA

VAERMKLWAFLAFAVVMTGVIYPMEGAWTWGGEAVFGMYTLGDLGFSDFAGSGIVHLAGA

SAALAGVIMLGARKGKYGPQGQTNAIPGANLPLATLGTFILWMGWFGFNGGSVLATASVE

SANSVAVVFMNTNAAAAGGLVAALIVARVLFGKADLTMALNGALAGLVAITAEPSTPTAL

QATLFGAFGGVLVVFSILSLDKLKIDDPVGAISVHGVVGLLGLLLVPITNGENSSFSGQL

IGAATIFVWVFGTSLIVWGVIKALVGIRVTEEEEYEGVDLSECGMEAYPEFITSK

>WP_007225392.1

MTSWDIAAIAAVLLVGVPHGGFDGAVARRLGWSKGIGGWLGFHLGYLALAAGVVWLWVQW

PVVSLAIFLAISALHFGHSDIADVPAPTTGSPSNRWLPLIAHGGLVSIAIPSLQPLAVQP

IFALLVGDEGAVMLLQAIRTLFLPWLLSFAGYAIYAVINPVWRKSLNSLIILLIVVFLMP

PLISFALYFCLWHSRGHTLRTWHRISAGSERRRSAIEAIIYSVMAWTAALVFFLYAEASL

SASLLQLTFIGLAALTVPHMLLVDLADKLNPQRPLP

>WP_140969888.1

MLGKLGELNEGYNVLTEMNGQCSDMLMDIGIYKMSNGKEELLFDNKNETAVLLLEGTIRL

EWEGMEQVIQRQSVFEENPWCLHVSKNVKVTITALSDSEVLVQKTDNDQEFASKLYTPNE

CQSVVAGDGVWEGTAQRVIRTIFDYNNAAYSNMVVGEVISYPGRWSSYPPHHHDQPEVYY

YRFNKPQGFGCAMVGEDAYRVVHNSFITIPGELDHPQATAPGYAMYFCWMIRHLENNPWN

DRIMEEDHKWLLEPNAKIWPEKE

>WP_140969579.1

MTEKMTRMTQFVKEEIANAITHGIGAILSIPALIILIIHASKHGTASAVVAFTVYGVSMF

LLYLFSTLLHSIHHPKVEKLFTILDHSAIYLLIAGTYTPFLLITLRGTLGWTLLAIIWTL

AIGGIVFKIFFVRRFIKASTLCYIIMGWLIIVAIKPLYENLTGHGFSLLLAGGILYSVGA

IFFLWEKLPFNHAIWHLFVLGGSTMMFFCVLFYVLPTA

>WP_040822867.1

MDKEMKILIVDDFSTMRRIIKNLLRDLGFTNMAEADDGSTALPMLRNGDFDFVVTDWNMP

GMSGFDLLKAVRADEKLKTLPVLMVTAEAKRDQIIAAAQAGVNGYIVKPFTAAVLKEKID

RIFERVGN

>WP_007224228.1

MARVLIVDDSPTETHRMTKILDKHGYEVITADSGEDGVAKAKETLPDVVLMDIVMPGLNG

FQATRQLSKNASTSHIPVIIVTTKDQETDRLWGQRQGAKGYLTKPIEDSALLNTISDVLG

>WP_007224227.1

MEGNFENLKVMVIDDSKTIRRTAETLLKKVGCEVITATDGFDALAKIADTKPNIIFVDIM

MPRLDGYQTCALIKNNSEFKQIPVIMLSSKDGLFDKAKGRIVGSDEYLTKPFSKNELIGA

IEAHVG

>WP_040541578.1

MALEYILPGTVSQESPAVMHTLEFKEEPGRVLLVLPTWVGDVVMATPFVRALFMRFPDAE

ITLLMNHHLYPLLEGSPWVQHCEFWAPRKKTAEAKQQQRELLNRLKARRFDLAVMLPNSL

RSAWLCFRAGAKRRVGFSRDGRGLLLTDKVEVPNRVAGGYQPLPLCDYFAVLGDALGMEH

PGDRLALFLTDQANDAVQSRLLKDGVLPEQPLVVLCPGANFGASKCWDPKRFAAVADRLV

NRHNAAIAISPGPGEEPLAEAIRDNMDAPSFLLTQPCLTLGELKSLIVRADLLLGNDTGP

RHFGRALDTPRVTVFGPTEQRWTETSHGDETIVNVDVPCGPCHKKVCPLDEQVCMTQVTV

EMVSEACEEQLSASC

>WP_169253931.1

MIAASALVLSACGSGEGGESGSDYLACMVSDSGGWDDQSFNQSGREGMENAKKNLGIEEK

LAESQGDADFGPNVDNMVQQGCNLTFGVGFLLEDTIQEAAEANPDLNFALIDSTFSDADG

KPVTIDNAKAVVFNTAEAAYLAGYVAAATSESGKVGTFGGIQIPSVTIFMDGFADGVDKF

NEDNKKDVKLLGWNKEKQDGSFSGDFENQGQGQELTKQLISQGADVIMPVAGPVGLGAAA

AAKEAGDVNLVWVDSDGYESTEYGDIILTSVVKQISQAVEDTIKEGTEDNFSNEPYVGTL

ENEGVGLAPYHDFEDKVPEDVKKDVEELKKQIIDGSLVVESESTPK

>WP_169253893.1

MNMLAMRDHSSDELRKKLLKRDLMPEAIDVLIEKLKNSRLLNDEEFAHRFARAQRENRKL

SRSVLKRELSKKGISPELASEAVADIDGEEELAREVAEKKAASTRRLDYAVRERRILGML

ARRGFPSAICIKVTRDVLTDD

>WP_169253863.1

MIPLALAALLIFFLFNSRRKQKARAEQIKSGLVPGATVMTTFGVFGTVLSIDEENNQVTI

ESGPGTVLRVHRQAIGQIENNQAAAPVDAPGADAPAADVDAAADDEKPAITDAELDAMNE

RKRAEKDTADDETAEDISADESAAKTEDADAVAEAEDVDIDAAETDADSAAADDDSTDST

DSDSDKKN

>WP_169253834.1

MGLDDHIFNRLLKERIIWLGSEVRDDNANAICAQMMLLAAEDPDADISLYINSPGGSVTA

GMAIYDTMQYIKPDVSTVAMGMAASMGQFLLSSGTPGKRYATPHARILMHQPLGGIGGTA

TDIKIQAELILHMKKQMAELTAQQTGKSLEQILKDNDRDHWFTAEEALEYGFIDKMVTRA

SDVENN

>WP_169252912.1

MKHLITGRRVTWAAIGMVGALALSGCGGAGGSSAGGGGDEVNVLMVNNPQMQDLQKLTAD

HFTKDTGIKVNYTVLPENEVRAKIGQEFAAQAGNYDVASLSNYEIPTYANNKWLTPMDEG

VADAEGFDQDDILAPMAESMTVDDQIYGEPFYGEGSFLMYRTDIFDKAGVEMPEKPTWDE

VAKLAAKVDGAEKGTKGICLRGLPGWGEMFAPLTTVVNTFGGTWFDEDWNAQVDSKEFKE

ATNFYVDLIKDHGESGAPQAGYTECLTNLQQGKVAMWYDSTAATGTLEADDSPVKGKIGY

VAAPVKETDSSSWLYTWAWGIQAAGKNQDAAKQFVAWASSKDYEETVAEELGWQHVPAGK

RTSTYENPEYQKAAEPFYQQTEDAINSADPESPGVQPRPTLGVQFVTIPEFADLATGISE

DVSSAIAGRTTADKALEKGQKEAEKVGDKYKK

>WP_169252886.1

MSLLGEPLSILALGLFNGFALCVFVLSLPPLRRPTLSSRIAPYLRDQESLVDIYAPPTPR

ADGFWGLAKSWLVSSTLWVTSRITTDATLRLRIDRLGGNATIERFRISQVLSILVGMIVA

GGLAGALSAQRGFSPIVTVVLIISGGVAGHVFNDWRLSQAIARHESRVLAEFPTVAELLA

LSITAGEGIVEALERVCRTCSGDLIDELRAALAATRTGTPLVEALDTMATRIAIPEIVQF

VDGLAVSMARGTPLAEVLRSQAADVREQSRRRLLELSGRKEIGMLVPVVVFVLPVTVIFA

VFPSLTVLDLSP

>WP_169252885.1

MSYSQFALLSGACLGAGLFLIWTSLWQRPQSKRTQSRWVRELDDMLTGAGFPRLRPAHLL

LISVAAFCIVTVVSTVLTGSWAIALCFGLFASWLPHRALQHRARSRQVMRRELWPETLDH

LNSGVRAGLSLPEALSSLAHRGPEPLRPLFEVFAEEYRASGSFALALERFRQVSADPVAD

RIVVALSVTRQVGGSDLGTMLRALAQFVRDDARTRNELSARQQWTVNGARLAVAAPWVVL

AFLSTRPETAVAYNSRTGLILLAAGFVVSLLAYQAMKRIGRLPAEPRVIEGSSLSAAHRR

FADNAGMSSGFTADSDDSADGRAA

>WP_169252597.1

MVTSAPRPPKLSLGDLVPGYLGDFGPEEFLEGMEFTDLDLAEADATQATFLDCRMTNVNF

GDAEAQIDLSGVRISGTEITDCRADTWTIPRGNLLHTDVSGTRIGAGVAYDSVWEKVRFT

NCRISYLNLRESKLTDVEFRDCKIDEIDLDRAKASRVAFPGSSVGVFQCEGATLGNVDIR

GLEPHKISGVHSLRGAIIDDTQLMLFAELFASELGISVE

>WP_169252584.1

MSTFTKVLDRWLTVTTAAAIIVMMLHTVTHALARSIFHAPIYGTNEIVEYWYLPIVALLG

IPAAQLQKEHITVTMAIERAKPATAALFTVFACILGALVSAAFAWFGLMKALENTAIGST

ADVSAVITWPVYYLVPIVFVLLVVLYILDAVAILRRRRTETGEEQ

>WP_169252403.1

MNWKSLVNRAARIGVREGLRYLRQSQSKKNSKGASQPTDARRPDTARSGGGGAASAGTAD

RSASSGQGGSGSYPGDYTGSITVSYSPDLDGDADPGEVVWGWVPFEEDHTQGKDRPSLIV

GRDGRWVLALMLTSKDHIPGGVGEVRQDRHATWMNIGTGDWDSQGRPSELRLDRIIRLDP

DSIRREGAIMPRDVFDRVAEHISG

>WP_169252333.1

MDILSSTLAAGGNAAAASESPIPVWFMITSGIIVVAILVFDLLLVVKRPHTPSMREASIW

VAFYVALALVFAGALFAIGDAQHGSEFLTGWLLEYSLSIDNLFVFIIIMGSFSVPRKYQQ

EVLMVGIIIAIVFRGIFILAGAAIISAFVEVFFIFGIFLLIVAYRQAFSSEDEGDGENGL

IRFLRKRINVVDEYHGNKLRVTLDDNKKYWTPMFIVFIAIGSTDVMFALDSIPAIFGVTQ

NAFLVFTANVFALMGLRQLYFLLGGLVDKLVYLHYGIAAILGFIGVKLIIHALHSSDWEF

LSWGHSIPEVPTWLSLTFIVFAMVVATLASLMKMKKDGISFRDMSSEESEGA

>WP_169252137.1

MNFMPGSTASNMPQSRYVMPQFEERTPYGFKRQDPYTKLFDDRVVFLGAQVDDTSADDVM

AQLLVLESQDPDRDITLYINSPGGSFTAMTAIYDTMQYVKPEIQTVCLGQAASAAAVLLA

AGTPGKRLALPNARVLIHQPAMQGQGQGQASDLEIQAAEVLRMREWLEATLAKHSNKEAT

QISNDIERDLFLTAAQAKDYGLVDQVLSSRKDAN

>WP_169251947.1

MNASLTDSSPAVPDGSGPVGSAGADDLPPADDHRIARRRSALRAELTKLAGQVKPKLRGW

FHAGAFPLSMIGGLALVIISPTIESRIAAAIFAVTGMLLFGTSAVYHRGRWRTRARLVLR

RLDHANIFLITAGTYTPLAVLMLTTDQAILLLSVLWGAAALGVAFRTIFTTAPRWLFVPI

YVGFGVAGVGYIPQIWATNFAVGLLVVLGGVCYIAGAVIYGIKRPNPSPKWLGFHEIFHI

LTILGYGCHLAALIIAAVAAY

>WP_169251583.1

MIVLTAALIAAGIVLAAPSDPGARLRELLHRAEDGESVPKGVRAVFRRGGHTADDQAERL

WAIAAVENCAHLLKVGMTPQAVMTTLSRHNDALAPISRAISLGEEPGRAIATRSSALSEA

AAHVLTGMAAVWTVSERSGAPAAEMILRYAAAQRDALDAERERRIAMAGPRSTVRVLSWL

PLIGVGLGLLIGVRPLELISGLPGQLSIGAGLLLYGAGRWWMRTMMLRAQR

>WP_169251255.1

MNGKQARTGRGRIAWTAAALALLAIAAVLITAGLLTDHSPPQPDADSAAEQSESRTTPSA

DAQPTDPSTPRTEQSSDGDQQAGMEASAPTRLRIPAIDVDTSLMDLGLTADDELEVPPLG

KDAPAGWYKRSPTPGEVGPSLIVGHVDSASEGPAVFYRLGALEPGDTVSVTREDGSEAEF

TIDDVTDYGKDSFPDYRVYGNTEDPEIRLITCGGEFDDDTGHYEDNIVVTGHLSDGG

>WP_169251032.1

MNIIDKYLSKVENVLAGGCLIAATALAVFAVLLRNITGDVLFWSEEAVIYLIIFSTFFGA

VVALRHNDHVAVDIMPTLLKGKAKKFFVVLGGLATLVYAGFIAYLSWALITEPFSRTTIT

PALKLPLWVVELSLAVGMTLFFIRAAEMTVRALRTPAAELDKDVLAEEAAAVGIAVEDIA

IVEDGRDRANRADGNDGGDRLGDGETDEKGDDR

>WP_169251018.1

MTRTLRRPRTLAAAAGIAALTLLATACGGGGGGGGEEGEQSADFITIATGGSSGVYYQVG

ATMSEILADELGADTSVQSTGASVENLTLIQDGGAELAFTQGDAVDQALAGEGAFDGKQI

DSLPVVANLYDQYVQLVTIEGSGIDSIEDIKGKSVSVGDQNSGVELNARTVVDAYGLSYE

DFSADYLPYAEAIDQMRNGQLDAAFVTSGLPNSAVTDLATSDDVKVVPFTGDGREKLLSE

HDYFGEGEIPAGTYGDSKAAETLTIPNLLVVSPSLSDDAVYDITKTLFDSIDKIQSSHNA

AKDITVDNAQDVPVSELAPGAKQYFDEQG

>WP_169250942.1

MRVIRFFEDWVVIAAFMVIVIVTFVNVLSRYIFKASLAFSEEITINLLVVLTMMGAVVGI

RLGAHLGFTYLVENTKAGTRRALLFTGTGLIVVFLAVLLIWGGEMTIAQGVRGRATPSLG

IPQWLFTLSIPLAGLLGIIRSIQALRTALGEDTSAEAVVDRLAGEATPTVDSSEFSADVK

GGRK

>WP_169250805.1

MSTSHSADQQVGHSAGRSADDSAAAGDQPLPSRRELRRREAEAAAAQGEPTAVYSDAPPV

YEQPQPTHAQPTHAQTTQAMPAVPQGHGGQSTQGRLGEDQPQNRPVTRRRRRRQPAEKEP

LGPIRGTVRTFGELCITAGLVLILFVVWQLWWTDIQANRDNEVLADELTQDWANQDPNEL

PDDPDEPVVAEPVGKNEAFGIFYIPRFGDDYYRTVAEGVDLEPVLNRMGVGRYPNSAMPG

EVGNFSVAGHRVTYGKPFNQIANLRPGDEIIVQTKDGFYTYTFRNFDIILPDAVEVLSPV

PDAPDFKGKDRILTMTACNPMFSARERYVAYAELTDWTPAGNGAPDSIKDSKAYDKVSKN

GGA

>WP_169253925.1

MRARTTQIAALAAVGVLTLSACSVTAPEALEEERLGCLVSAPAGFDDHSAGALTLEETEL

ARGAGVFSGTSSQRVSGGSATSAALDRMHGHDCALTTVIGPGGADELADFAAAHPDDLFL

GVSPGTDDLPKNVLSMDFDLVPPAYIAGFTAATASETGKVGIVVSHGFPQADRILAAFDA

GVDLYNKEKDEDLPKAESYHPSSDRAASANGSPRAIDDTRDAGKDYFERSFDADVDVLVP

FGSAAAMGVVTSADEKRTDLTAATEDPESGDDGPSLPKVIWYGTAGGFSKAIVATVEPNV

RRGLRTMFPDWPQSRNPDEVAPAPKTEPEEIGGFEVSERRYEGTIDNGGVRIVAEDGFLS

RVSDAGRGITDLRERIKSGEIDPEKG

>WP_169252724.1

MRQVEPSVELLAKPDIDWEAMRTYLDEVGGTSWADRVEDAESPDAEDLLEFSGRMCYRSW

EPGLNPNVSRVRTDSAQYLGNVVKSQHGSVLEHANFTFVLHNVSRVLTHELIRHRAGSAF

SQESLRYVRLTDIPFWFPEWAREDPELMERSLQVLDTLEEHQKWMAEHFELDEEGTKFSH

KKHMTSFMRRFAPEGLATGIVYTANLRSLRHVIEMRTAKGAEEEIRLIFNKIGEVMREEA

PAVFADYEVVDGEWIPGTRKA

>WP_169252371.1

MSPITDEFSSDDGLTRAEVRAKKRRRMLRRRRTTTIVIICILVFGIGGFFGVRAAGGVFD

DLFGPKGDYEGQGTNEVSIEIAPGSSARTVANQLVEAGVIMNSEPFLDEIERREATIQAG

TWTMREKMSSEAAVEALINPIAPPKITVAEGKQVEEIKAIMIESGMNADEVDKAIDDKTP

KDYGLDIEAPSLEGYLYPATYDLNKQKTAEDIVQEMVDKTETELDELGIESKDANRILTL

ASLVEKESPGDPEVRSKVARTFLNRISEKSKTGGLLQSDATVAYIHGARSDLTTTKKERQ

SDSPYNTYKKKGLPPGPINSPSRGAVEAALEPADGDWQFFVATNPDTGETKFADNYEDHK

KNVEIYRKWLREHRKDNG

>WP_169252360.1

MRIVLGVAGGIAAYKAAHIIRRLRELDHSVKVVPTANALKFIGAPTLEALSGQTVTTDVF

DEIDTVNHVRIGQDAELVIIAPATADLIAKIAAGRADDLLTASVLTTTAEVVVAPAMHTE

MWLNPATVANIATLRSRGIHVLDPAVGRLTGPDSGPGRLPEPEEIVDFALSVKDGTADDS

ADALNRPSGALSGRRIVISAGGTREPLDPVRFLGNRSSGKQGIALAKAAHAAGASVELVA

ANIDTGLLSGLPADITVTPVESTLELQEAMHTAQARADAIIMAAAVADYRPAETADSKMK

KSGDDGLTLRLTQNPDILRGLVAERSGQTGLRRQIIVGFAAETGDSDTTALDYARAKFER

KGVDLLVFNDVSDDRAFGHDDTMVQIISSDRGDIVVGEEFHGSKDHVSQKVIAAVSDQIT

HVESTT

>WP_169252217.1

MFSSAEELVNFISDNDVKFVDVRFCDLPGVVQHFNLPAASYGTEEITEGLLFDGSSITGF

QGIHESDMKLLADVTSAYIDPFREAKTLVVTHSIVDPFTDEPYSRDPRQVAAKAEAYLQS

TGIADTAFYGAEAEFYLFDSIQYENTPGNSFYRIDSEEAAWNTGADEAGGNLGYKTPHKS

GYFPVSPQDHFADIRDEMSLTLEQIGFEMERAHHEVGTAGQQEINYKFNTLQHAADQLLD

FKYVIKNTAFANGKSATFMPKPMFDDNGSGMHCHQSLWKNGEPLFYDENGYGGLSDLARW

YIGGLIEHAGAVLAFTNPTINSYRRLVPGYEAPVNLVYSARNRSAAIRIPVTGSSPKAKR

LEFRVPDPSSNPYLAFSAQLMAGLDGIRNRIEPPEPIDKDLYELPPEEAKDIKLVPGTLD

EALIELEKDHDFLTAGDVFTPDLIETWIRIKRENELDVARLRPTPTEFELYYAL

>WP_169251582.1

MSEWLDASLVADVRKTLLDKPGPVTTAAVAEAVQRTGRVLGSSALLELVTRLSAQLSGAG

PLQSVLEVPGTTDVFVNGPREIFADTGGGPRLLDLSLGSEEEVRSLAVRLAALGGRRLDD

SSPYVDVRLPDGVRMNAIVPPISGDTTTISFRVPKRSGFLYSQLCDSGFVPDEISPLIAE

AVTSRANILISGGTGTGKTVLLGALLSLVETIQRIVIVEDSRELIVTHPHTVQLAARQAN

VEGGGEVTLTDLVRNALRMRPDRLVVGECRGAEVRDMLTALNTGHEGGCATIHANTAEAV

PSRVAALGALAQMSPDAVYSQFSTAIDLVIHLRRCGSERGITELAIPTRAGAEAVVMEPV

WGRPSPSTTGTVNRRALARFEAVLEQKSAQ

>WP_169251567.1

MSVNIGVVGATGQVGGVMLDLLADDPGFEIGSLRLFASARSAGKTIDFKGQPITIEDAAE

ADPSGLEIALFSAGGATSKAQAERFAAAGVTVVDNSSAWRSDPEVPLVVSEVNPDDLDEP

PKGIIANPNCTTMAAMPVLKALHDKAGLTRLIVATYQAVSGSGLSGVEELAGQLEAGLPD

ARKLATDGTAVNLPEPNNYVEPIAFNVLPMAGSVVDDGQNETDEEKKLRNESRKILGLPD

LLVAGTCVRVPVFTGHSLSIHAEFDSDISPEQAAEILSQAPGVSLDEVPTPLKAAGQNAS

FVGRIRADQSAPVGKGLVLFVSNDNLRKGAALNTVQIAALLAAKLEAKAA

>WP_157361095.1

MGDPVSGGGGWRHADRVVAVESVVSGTVECMARKTLTAAPRLEKLRLEAIAVGDADHLQA

HESYSGGRYVASDLRERELSGISFSECEFVELEASETDLRAATFVDTRFERLNAPIFTAP

RSSFRDVSFEGSRLGSAEFYEANWSSVHFVHCRIGYLNLRGARLEDVLFTDCLIDELDLG

AATANRVSFIDTQINNLDLTRSTLTNFDLRGVELRQLGGVEYLKGATLNSYQLSELAPLF

AHHLGIVLDE

>WP_148224521.1

MGNTTFSDGAIKGLAAALVVCLHFACASLASAEEDASSPGPREVVAAATDNIMALAREAP

AYFDTDPDRYTVAVGEELDRVVDFRGFARGVMGRFASKELYQGLDEAGRNQLREHLMKFT

EVLRSGMVNTYSRGLLAFGGSQVELGEVDMAPGSTRVASVTQRVFGDDGKIYTVKYQMGQ

YRDGRWKLRNLIIENINLGEIYRGQFEAAALEAGGDLNTVIANWDDNRVKSLSTEE

>WP_007228891.1

MYYGEKLNSISHLVGAVIALIALGALLALGIQTGDPLVIVGFTAFGLALVLLYTMSTLYH

SFQEPRLKKAFQLLDHISIYLLIAGSYTPFMLVSLGGSEGKMILSLVWTLAIIGILSEVF

LSGRVVKTIQLVIYLSMGWACSLEFASLKAALPEVGFFWLTAGGIAYTAGIVFYLLDKMN

RLDHSHGIWHFFVLVGSVCHLIAVIGYVR

>WP_040823262.1

MVDVALFPVPNSVNFPGVPCSLHVFEPRYRQMVRQCIDQNLLMGVCHTEKVLHRKEREQT

LEEALNSNQTTYKPRGIFSAGPVELLEELEDGRMLIQVNNEVRLQLGEEKQTLPFGIWAC

EELVDEALDETGELALNQSQSKILQRLLAVTHGNEDAQDMLNSIHWRSMPAQTFSFAVAG

LLGMPPETSQALLEMTSAQMRLDTVLEMINTMGTALS

>WP_009774169.1

MSIQHKLPRSSGSALTILMVLALAIAGCSVNSSPRSEASNASAIPPGAFSATVNYVHDGD

TLYLDTGREELKVRLIGIDTPELAGQQRPDAEECYGAEARALLRDFLPEGTQVWALEDRE

PEDRFGRSLLYVYLDDGTFVNLAMIELGAAEAIKVGLNDQYWPELRDAEDAAHSAQLGMW

GAC

>WP_009774019.1

MKRARSQLPEVASVTQRLAVLLNAGLTPSSAWFHVARGRSADGVAALVALAGEEPGGAAE

RIVRAAEGLAPLDRQAWNSLAAAWFVAGQVGAPLAAALRTHARALRMLVHVQREVATALA

APVATARMVLALPAIGLVFGALLGFDTIGVLVSTPIGWGCLVVGGALIAAAVRWNRRLVR

SATPTQAAPGLECELMAIAVSGGGSLVNARVVVAGALERFGLMGDGDHLEGVLRLSHEAG

VPAAELLRAEADELRLAADADARAGAAALSVRLMLPLGLCVLPAFMVLGVLPLMVAVISS

TVAGL

>WP_009771703.1

MSLILGIILAVGVTLVAAPFLWPAQGERRVRGRSAWSLRLRERLVQAGLPTTSPSIVLIV

SVVFSVAVAAVTFVLTSVIVVVLCSAALALVLPTLAISWRARARRTATQIVWPDVVDQLV

SAVRSGLALPDSLMTLSQTGPLVTRSAFAAFAARYRATGNFSIAVDELKVALADPVADRI

LETLRMSREVGGSELTNVLRSLSVYLRQEAAIRSEIEARQSWVMNAARLGLAAPWVVLFL

LTTRPEAAAAYNSASGVALIVAGLILSLVAYRIMAGIGRLPQQPRWFA

>WP_007234989.1

MTEIPLFPLSSALVPYGYMPLQIFEQRYLDMVAACMRTGTGFGVVWLREGSEISGGSHNT

PDVGKYGTHARITDFDQLPNGLLGITIRGEERFDIAEVWRDSSGLIRAKVSMEAPLAPAS

MTDEWRSLEIVLRGLESHPHIQRMNLTIDYNNAWEVAFTLIQLLPFDEAIKYELLGLSTL

DELIVELDILLNQISGEDG

>WP_007234840.1

MFVCICNGVTDSSIRREMEAGATSFADVQNRLGVARQCGSCEHLARAIVNEFSKPDPRYF

YNACSDESMAAVA

>WP_007234584.1

MTEDKEYSRMRWASRRGLLELDLLLEPFMEACFRALEPQLRDDYQQLMGHEDQDILNWIM

GREALADETLTAITEQIRVHNRSKLR

>WP_007230552.1

MNQTLLIAQVAAFIATSCTVLFIGFSIRDALKRREQLWIKIRASHVSKAEAGAEFVRTVL

LETVRPMSSVQKLLRQTQKNLLRAGVGRNAEDYVADGLWQGLLGGISIMLAVSLLSSPVS

GLLLGTAGGVLWAMWIKPSMLDSNATQRSRLIYRRIPYALDLSVLVLETGGTLREGLEEI

SLQNDPLAEELRITLLEMDSGSTQAAALRGMGQRVGLESLETILTAINRGEETGAPMVAT

LITQAEMFRERRLQEIEKMAVEAPTKMTFPNMMIMVSVLLLIIGPLLIQLVSSGLF

>WP_007230536.1

MGLIIAYALIAVSVTALVYAGLQSFVPVAVTRWRSDQEEIDDKLQNIFYTSSEARTFLIL

KYGGTLAAFFIGLWFMNSLVFGIFLGIVIYLLPEVLLDNILRRRRERLEAQTADVMTALS

ASIKSGMTIEQAFSEMVDNMYPPISEEFALIRERIDAGQPVIAAVKSADERLQVPRLSLI

FQTICISLERGGRLASLMDRLAESTREIERVEERVRTETAGLRLSARIMFLMPFFICGLL

YLIEPDQVMLLFDNLVGNIVLVIAIAMDISAYFIMKKLIELDI

>WP_007227341.1

MAPSAIQSLDALEQRFREDAVEAPVQDLERMEWVGTCLSIAGVPLLIGEGELEEIIETPN

VMAIPGTKPWVQGVGSHMGGLIPIISGDVFFRKRPYSGRVRDYCMVLRRPGFYFGITLSG

LERDMKFLVETRDMTVTTDPDFAEYTLGGFPDQDRVLAVLDIDKLIADSDLSNAAANDPD

SPEERTND

>WP_007226034.1

METIPLFPMHAVLFPHGRMFLQVFESRYLDLIGQCMKEDSGFGLVWLKQGQEVYRSNELV

DPQLAQIGTYAKIVDWDSLPSGLLGVTIEGSDRFRLLTSYQRKDHVHMGEVEWIETAGAT

ELPENYAELWGLLQTLLDHPHVDRLKLNPVVNDVNAVSCLLAQLLPIEERVKFNLLAAAE

PLDRMARIMTLLDQYSE

>WP_007224867.1

MQKIRVYWRDIPSQVIIKRGRLRAKAPLTQRFQVAIDRAAMTAGRGSSEAYVADWRRETT

SISGDGDLGQLAESEALMLENQYDDDRLKQLVANRGLEAE

>WP_007235031.1

MKHSGILVVDDESVIAEELCEFLSSFDYTCQKALSVNEALALIETNLHITLILTDMRMPG

RDGAELIQALQEMPGRQFEYLMISGHLDADEDLKHINNEGVTLMRKPIDIDALLLFLEER

EFTAVPNEN

>WP_007234880.1

MQKEMDTSSLKVLVVDDEAFVLKLTVRILSKLGYDNVVTADNGVVALGEIDNVTTPFDVI

ICDLNMPEMDGIAFMRHAADRNVSAGMILLSGEDERMLETARDLAAAHKLHILGVIPKPL

KPDALSNLLNTFQPTAVVEKQGWHQEGISETELLDGMNSDQLHLVYQPKVNISTGEVTGV

ETLARWMHPEKGLLGPGAFIPLAEETGHIDQLTCAIYRKAMHQAGDWLAQGITMKISVNI

SVNSFTAPGFTDFLIETAQNEGMDLSNVVLEVTETQVMDNALGILETLMRLRMKRFALSI

DDFGTGNASMEQLKRIPFSELKIDRAFVFGAAENAGARAILESSVTLAKSLKMDIVAEGA

EGREDWDLLASLGVDTVQGFYCAKPMNNADLMSFLEDWTGPH

>WP_007235808.1

MVKHSNTEDPWALKVLAAVAELGPQRYYDPVITSEVVKNDLVSLIRASQKLKEKVAIASP

EARHDRRNIIGAIRGYSEMLLEDSEVLPAAVRAHLLQILAAAKNEPKPASESATPTKSVT

LLPSEEPGVILAVDDLPENRELVSRLLQKTGHTVISAESGEEALELLDTMGVDVVLLDLV

MPGIGGAEVLKRLKEDERLRATPVVMISGQQDMDQIVMCIEAGADDYLLKPFNPVLLQAR

ISAGIERKRWHDREELYREQLERREQFIRATFGRYLSDDIVDEILERPEGLELGGDLREV

TIMMSDIRGFTTLVEHLPPQQVVTLLNRYLGRMTEIILEFGGTIDEFLGDAVLAVFGAPR

RNDDDPDRAVRCALVMQEAMADINIANSADGLPEVEMAIALNTGSVVAGNIGSERRSKYG

FVGHAMNVTSRIEDVAKPGEILISQTTYEKLESDYKFGNSRSLSVKGIEAELRVHAVLGG

IQ

>WP_007225300.1

MDTAEQQLLLIDNDEVERKSVAAYLKGAGFIVLEASNVSQGLDILADHQPEVVLCDLNAT

GTDSGPLQAIKSDFADTPVIVMATDGVMSDVVWALRYGAADYLIKPIADMEVLEHAISRC

QEQRQLRQQNLDYRQKLEQANQGLQESFKVLELDQLAGREVQLKMLPPTVKQFGKYQFSH

RIIPSFYLSGDFIDYFTVGDDFVVYFIADVSGHGASSAFVTVLLKNMFARKRSDYLHQND

ASILSPAAMLDIANRNLLTTDIGKHATLCVGVIDLRTDTLSYSVAGHLPLPMLTVDGEPQ

YLQTEGMPVGLFEAAEYTEATISLPPSTVLTLFSDGILEAISVKGVLGQERFLLEQLSRG

PNSIDGVLEALKLNEIGEVPDDIAVLLITKDVHLGDSIIHADTGSDSE

>WP_050756508.1

MDLILASTSPYRRQLLERLQIPFRCESPNVDETAHPGESPAALAQRLAAAKALDIASTNP

GAFVIGSDQVASLSGSCIGKPGSHAAASKQLHDSAGQRVDFYTGLSLINLSIDYHETLIE

RFSVVFRELESLEIETYLQKEKPYDCAGSFKCEGLGIALFEKMIGDDPTTLVGLPLIATC

RLLKAAGAPVLEH

>WP_009772888.1

MRLYLASTSPARLATLRAAGVDPVLLASGVDEDAVAAAAPGPLDGPALVELLARAKAEAV

VGSRINNEPIDGFILGGDSAFELDGELFGKPHEPEIARRRWHAQRGRTGVLHSGHWLIDH

RGGQLRGATGAVSSASVTFASNITDAEIDAYVATGEPLKVAGSFTIDSLGAAFIERIEGD

PHAVVGLSVSLVRQLMRELDAEWTDLWNIERPTL

>WP_040811778.1

MTMKKILVVDDEPDLRDMLRFALETEGFEVLEAADTQKAYWLITDQDPDLVLLDWMLPGG

SGIELLSRLKKEEATQSLPVIMVTAKAREEDIIQGLDMGAHDYITKPFSLKELLARIRTI

FRHTEDDDVNHQLRVGDLVLELDNRRVTLGSQVLMLGPTEFKLLQFFMLHPERAHARKQI

LQHIWGNNACVDARTVDVSIRRLRKTLQSAHPVYSELIQTVRGTGYRFSPRDLVAA

>WP_007227708.1

MSEPTVLVVEDEKAIRDMLRMALEVAKYRFIEAENIRDAHVLIVDERPDIVLLDWMLPGG

SGLELLRRLKREDNTREIPVIMLTAKAAEDNVIQGLEVGADDYVTKPFAPRELIARIQAL

LRRSAKDSAQGRIGLNGLVLNSDSRRVFAGEIALNLGPTEFNLLQFFMSHPERAYSRSQL

LDQVWGANVYLEERTIDVHIRRLRKALQTDHADYGELIQTVRGIGYRFASREHS

>WP_009773595.1

MKFQVNRDVFSEAVSFAVKLLPQRTTLPILSGVLIEATDEGLTLSSFDYEVSARTQIKAE

VDEPGRVLVSGKLLAEIASRLPNAPVRFSTEDNKITVACGTGHFTLSSMPVEEYPTLPQI

SDQVGTLKADLFSAAIAQVAVAASRDDVTPVITGVQLEVSQNNISLVATDRYRVAVRDIE

WDAGASGVESATALVPAKTLVEVGKTFGNSGEISVAITSTDERELIAFHADNKTVTSLLI

KGNFPPVRRLFPETVDNFAVMNTAELIEAVRRVSLVLEREAALRFTFTTEGVTLEAIGSE

QAQASETIDAFLTGDDTVVSLKPQFLIDGLSSVHSEFVRISFTKTENPNKPGPVLITSQS

SKDQPGSDNYKYLLQPNLLLR

>WP_007225086.1

MVETLLIPLMLIVLAVFFLLSPVMMNRSLQRSSRSGVNIEFFKSRLSELESDRARGILDD

DEFEQLKIELERRLLDEADSGHTAPSAHVKTSFKTAIMLALLIPIVAVVVYQQTGAKADW

DIAQTLKNMRLKTADGEAAETDVKQLIRQVEERLEQRPDNGSYLMLLANQQMGLRNYPAA

AAAYQRLRTIYPDDASVLAQYAQAMYLSSDRTLTTKVTDMAELALRQDPQQPTVLSMLGM

AHFEQGDYQRAIDYWQRLLPSLGPVSPNRKIIMAGIEQAKSRLGSSDTTSIDRPDVIKNA

SIQLSVSIDEGIIASSDSVVFVFARSASGPRMPLAVAKLTVADLPVILTLDDSMAMAPGL

NLSSQKEIEVVARIAKNGIANPGPGDIEGRVGPIKLEEVDGVVAIAINKTL

>WP_007225370.1

MSYVINFEAVPEELKTLPQWVCWKAVVRPNGKITKVPMNPLTGIKASSINSKTWASFDKA

ATGMNRHGYDGIGFVFVRGDGLVGVDLDNCMRSHGQLETWAQDIVDRLDSYTEVSPSGNG

VHIICYSGASGLSYNKDGREMYSEGRYFTVTGNEYYVRGHSNEN

>WP_007229947.1

MTLLNTKIHLTDNQMHQNQDILGNISTAVVSLDSELRVVSLNSSGQDLLEASEARSLGQP

MHKLVANPEALMEVLRQVRADRSPLARRGMPLMLLSGREIHADLMLTPVSNSEHGINILL

ELQPVDRLLKISREESLHHAQETTREMIRGLAHEIKNPLGGVRGAAQLLARELSSAELEE

YTNIIIREADRLRDLVDRLLGPNQQMDSQCMSIHEVLEHICNLVRAETDNRVELVRDYDP

SLPDIIGDRSQLVQAVLNIVRNALQAAPSEEDCVITLRTRPQRQFTIGNQLHRLLCRLDI

EDNGSGIPVDMLHSVFMPMVTGRAEGTGLGLTIAQSIITRHGGMLECSSEPGHTRFSIYL

PMDLNHA

>WP_040821591.1

MSTANTVWIVDDDRSIRWVLEKALNQAGITTQTFDSGETILNSLRQNTPDAIISDIRMPG

MDGLELLGKINETHPDLPVIITTAHSDLDSAVASYQGGAFEYLPKPFDIDDAVAMTERAL

LHANEKTADSTEPEENSSSTEIIGEAPAMQEVFRAIGRLSHSNITVLINGESGTGKELVA

HALHKHSPRSSQRFIALNMAAIPRELMESELFGHEKGAFTGATSLRPGRFEQADGGTLFL

DEIGDMPSETQTRLLRVLADGEFYRVGGHVSIKVDVRIIAATHQNLESLVKEGRFREDLF

HRLNVIRIHIPSLRNRREDLPKLMQHFLLKASEELNTETKLLTPESEHYLSKLDWPGNVR

QLENTCRWITVMAAGREIHLEDLPPELLDQTIADGGTDDGNWQDNLRRWADQELSLGKSN

ILDTAIPIFEKLMIDTALKHTHGRKRDAAVLLGWGRNTLTRKMNELGMNTTIIDSTES

>WP_007229945.1

MHSQAKVWVVDDDSSIRWVLERALKQAGINNESFSDADQLLKRIVSETPDVIISDIRMPG

TDGLELLSQINASHPELPVIITTAHSDLDSAVASYQKGAFEYLPKPFDLDEVVAITERAL

AQVRERSIEAPVLEELPETEIIGEAPAMQEVFRAIGRLAHSNITVLINGESGTGKELVAH

ALHRHSPRAASSFIALNMAAIPRDLMESELFGHEKGAFTGANAKRAGRFEQADGGTLFLD

EIGDMPAETQTRLLRVLADSEFYRVGGHTPVKVDVRIIAATHQDLEELVRRGDFREDLFH

RLNVIRIHIPKLSERREDIPRLMQHFFQSAAEELGGEAKILLPATERFLSNLDWPGNVRQ

LENTCRWITVMASGREVHITDLPPELSRDVVPDPQSTDSDWRAMLQNWANNELGQGKQQI

LEQATPAFERVMIEVALKHTQGRKRDAAELLGWGRNTLTRKMKDLEM

>WP_148224536.1

MPKLLIPIVVTLLGTVVLTIGLSFGALEQRKTQLAVSAQDMVSINATLSPLLHFRNQSAI

REALLLKLRLETKPQKIWLRVLHVDGEILAKAPRRDSDRGTTQFIRTFRNRLEVLTAVDL

FAHNSEAHYRGALAAIPFMDAQFKMSTPVFSLIDPLRTDVPRSAYQQTLLQKADQPLPFV

AGYIEQGIFLGDILETIIPTLWQALIMSMIISATMLLAFYYFAVRSRVSISQRTAQTERS

DQKNLPQKMESARSVPTNDETTSAPEDRLTEPSLDETRSNIGDPATLDPVTSLPDRHQLL

EHMAQGMRVAAAEHRCMGLVLIEVCSIRDILRTRGREVSDNVLREMTSRILNSIRRSDFA

SRGYDALGEAILDADQFCIVLCDLDNIQGVGSAAERLLGQLRLPVTVADEALCLNVVASA

ATAPQHSKTPEGLIIAAKSALIQARESRAPNTILFSS

>WP_148224448.1

MFAPTNLDLRGRTPPGYIAVEGPIGVGKTTLARRLAEAFNYQVLLEDAHENPFLDRFYQN

RKEAALATQLFFLFQRSQKIADLRQTDIFEPVRVSDFLIDKDPLFARINLDPDEYSLYEK

VFQQLTIDAPLPDLVIYLQASPDRLLERILSRGVSSERGIDREYLEQINEVYSEFFLYYD

AAPLLIVNANEIDLSQGDEDFSQLVNYLLDIRSGRHYFNPTFFG

>WP_148224378.1

MALLTVLVVILRYGFGVGAIAAQESVIYLHGALFMLGASCTLQAGGHVRVDVVYQRFSPR

ARAWVDALGHVIFTLPLCAMVGFASQDYVFESWVARETSPEPGGIPAVFILKTLLPVMAI

LLALQALSEIIKAVKTLISEVSHCD

>WP_040823729.1

MNDLAPSNIPYAYGGPLISGSLRDQPADFFVEENLGFEPEGEGEHVFLWIEKTDINTQQL

AGDIARLAKLPTRQVSYAGMKDRRAVTRQWFSVHLPGQDNIDWQALNSGQVRLLKQVRHL

RKLRRGAHRGNRFVIIINDVSGDTSQLASAVATIARRGVPNFFGEQRFGYGGSNLMRARQ

LFSGQFKPKKHQRGLYLSAARAYLFNQVLAQRVEANNWDQLTSGELLMLNGSHSVFAQGD

TIDLDARLLDGDIHLTGPLYGKSGSLAPTAEVAAMEADILQATPDFTAGLLQAGLKAERR

ALRLLPVDLQAQLSERQLTLSFALPTGCFATALVRELVNYTETHNHV

>WP_040811348.1

MYPLTRSLLVLLLSAFVSACATSPPSDTSNVCAIFREKSGWYNDAKKARARWDTPISVMM

AIMHQESRFVATAKPPRKKIWGIIPGPRPSDAYGYSQAKDATWEWYERSSGSYGADRDDF

GDAIDFIGWYNDMSFRQNGIAKDDTFRLYLAYHEGHGGFKRKTYRDKQWLVDVARKVDGR

ANTYNTQLKGCVKSLEDDKWWDFF

>WP_040542916.1

MIRIMLSVAVCVAQQAAADDINGVWKHADEPGWIEIQLERGSGTVERNDKFPERVGREIL

KDLATGGEAQTWSGLIYVEKMGEYKNADIILASPDRMKITVKVGFMSRTIGWQRVDEVPA

AP

>WP_040542885.1

MRILLVEDDVQLGESLEAALRLEHYAVDWLRSGEPVRATIGATPYDLMILDLGLPEVPGI

QVLRQTRADKHDIPVLLLTARNTLDDKVDGLDSGADDYLTKPFEIDELFARVRTLLRRRG

EGRSQQLEARGITIDPVDRLVIFEGEMLDLTAREYAILEILIRNAGRFVSRPRLEEGIYS

WGEEVGSNTVEVYISRLRKRFGSDCIETMRGVGYRISQ

>WP_040542483.1

MSFVHKLVQTIDAFTDRSGRVLAWLALAMALLITAIVIMRYGFNTGSIFSQELVTYMHAT

LFMLGTAYALKHGAHVRVDIFYRQFSARGKSWINALGGVVFLIPLCLFIVGVSWNFVNES

WAMRETSSELGGIAAVYLLKALIPLMGINLLLQALAETLRSTLELVEGNT

>WP_040541029.1

MNRVTVLLSAIALLIAAISVYLSFRLLDPTPPKTLILATGTAGSAYEEMGQSYRKILKES

GVEVQLLASGGALENLELLKSGQADIGFLTMGYPAGQDAVNLRSLGAMFFEPLWVFTQDN

DLLEGNLDSLRRTRISIGPSKSRSNSASRKLFELNGLEISDLNLFELDPTTAAQQLKQGT

LDTLFITGNSISPVIKQLLSSRETVLVDFKRADAYVALFPELTKLVLPAGVGDLALNMPS

SDTRLLAFTAMLGVNKDLHPITQSLVLEAAERIHAKPDLFHQAGVFPQARDQLIILSDSA

KAYYADGRPLLLRLLPYPVAVLFMQLIAAAIPLLGIAYPMFKLLPSAFHWIMRHQFYRVY

SELRQIDRSIGNITEAELKTHLERLENLEQKVTGLKVPITYSTMLYALKGHIGSVLKRVR

DALG

>WP_040541869.1

MNEALQQVINRNDTWQGHLAGQVLSANGDSHWDEDRLSTGYSTLDKELRSDGWPLGSTVE

VLSDGCGLGSMGLFLPAMEKLSAEGRWQVFIAPPFTPYAPLLKARGIDTDQILLVHPKSR

EDLLWATEQALRSTTSSAVFSWLGADEYSYSELRKLQLAAASGDSLSVLFRPQEAARNHA

PASLRLQMREYRKVHILKQRGGNQYIDVTLPPSEDVPEHPQLWEVPSWQASPGQASPGQA

SPKAQPAFSFA

>WP_009773271.1

MISHHYRRAMLPIATLGIAGMVLSGCASGSGGTGGPGDAGDPDGIVTIYGTIADTEAELL

EESWADWESENGIDIQYEASKEFEAQISIRAQGGNAPDLAIFPQPGLLADLASRDYIQPA

PAGVQANVDEFWSADWAAYATTGDTLYGAPLMASVKGFVWYSPADFADWGVEVPETWDEL

LALTQTIADKTGTAPWCAGFGSDAATGWPGTDWVEDLVLREAGPETYDKWVSHEIPFSDP

AIVSAFDSLGEILLNPEYVNAGFGDVKSINSTPFGDPARALGDGTCALHHQASFFDGFIQ

DPKNGNATVGPDADIWAFVTPSVEAGGNAVTGGGEIVGAFSNDEETIAVQEYLSSAEWAN

SRVKLGGVISANNGLDPASASSPILQQAITILQDPDTTFRFDASDLMPGVVGAGSFWTGM

VDWINGKSTEDVLSTIDASWPSE

>WP_009773251.1

MKRTATSVVAAVAAVGLTISMSACSTTSAAGSGDAEGPLTVWVMGDSGANFEMLVADSGI

EVEVVAIPWDSIDEKLTTAVASGSGPDILQIGLSKLRTFADAGALLPLDDEIANHPGIDP

ANFPAGVSGTATSVGGEIVSVPWTSDTRVLFTRTDILSEAGIDAPPATWDELRADAKTLA

ARGDDQYGYYIPQWDAPLPIEMTWSMGGEVIDADGNVNFDTPEFQKAVDVYTGLYADGSV

PVNGDFDQTQGFISGVAPMLVSGPYLGRGIADSAPELDGKWQASPLPAGDGGSISLFAGS

NLGVWFNTDQKETSLDLLEYVSQPEQQLEWYSMTGELPTVSSALEDGDLNSDPNVQVYTD

QLKTAKVLPLVSNWDGAVGTELLNALNAIVLTGADTKSSLDGLYSTTAGLTIN

>WP_009772227.1

MTDQNTNPTAPRRVVVAEDESLIRMDIVEILRDAGYEVVGEAGDGETAVALATELRPDLV

IMDVKMPQLDGISAAERLSANHIAPVVLLTAFSQKELVERASEAGALAYVVKPFTPSDLL

PAIEIALSRYAQIITLEAEVSDLVERFETRKLVDRAKGLLNEKMGLSEPDAFRWIQKASM

DRRLTMHDVSQAIIDQLSAKK

>WP_009771998.1

MHEIICPHCKKAFTIDEAGYADILKQVRDREFKTELHAQLALAEKEKIIAVELAESKIAS

GLGKEAAKKETEIEHLKAELKATDMEKQLAVKDAVSAVEKERDEAKNEREKNVVEKDAAI

ELLRAELKSTELAKQLAINEALSAVERDRDDLVRNLKATEIEQKLLESTLKEQHSTEVRI

LTETIDSYKDFKARLSTKMVGETLEQHCEIEFNRLRSAAFPDAYFEKDNDAKSGSKGDYI

FREHSASNVEIMSIMFEMKNESDMTATKRKNEDFLKELDKDRNEKGCEFAVLVSLLEPES

DLYNGGILDVSHRFPKMYVVRPQFFIPIITLLRNAALSTVQVKLELARVQEQNVDITKFE

GNLRAFKEGFSRNYSLAADQFQESIKRIDEAIKDLEKTKENLLKSSNNLRLANEKADGLT

IKSLTRGNPTMAAKFAELESPDDPENFK

>WP_007235241.1

MKILLAEDDAQVRTELRELLVEQGYDVTTAIDGIDAFEKFRADEMIEILLLDIRMPRATG

IQTLDAIKKIESANERVFETLFITGASDNNAIVSALKLGAFAFLFKPIVVEELLKELSEA

TDSINQKHYRNFQNSMFNPNLESRAPGKSGSIKEGMGATSEIAAIGAEHYAPGIEQHVHR

ISEMALCIAVRLGLETQHCQQIRLASLLHDIGKLGGPTDIYTAERALTEEEFEKTKDHTR

LGAALLEHYDDPIIEVAQNIALQHHENWDGSGYPAGLKGDEISIEAAIVHAVDTYDNLRS

HRPYRAALPHHVAMEILVSGDEKSNPDHFHPGVLQALLSQHREIEAIYERYRPIDVESKI

TDQTPA

>WP_007235034.1

MKVLVVDDESDIREELGEFVEQLDFSVVLASNGEEALGKYFDDPEISIILSDLMMPGLNG

LEMLDNINSAPGAHQRVRRVIFMTGNGNTQSVIRAMHLGAKEFLLKPVDLDQLERHIMSA

KREVATDRARQTEEILLKQQVSLNNAQISSLNRDVEEAYAEALACLAAAAEHKDPETGQH

IIRIGEYAAVLAQALGWDKEWSEMIRLAAPLHDVGKVGMRDSVLLKEGPLDDDELHHMRQ

HPETGYQILSVSNYPTMKMAARIARCHHERWDGTGYPRGLKGSEIPVEASITTLVDVYDA

LRSKRPYKPAFDHKQVMDIILNGDGRTEPKHFRPDVLAAFEAAQDKMADIFERLSDDTDS

SSHTSSSKREVL

>WP_007233471.1

MSELLIDVGNSAVKWAVCHGLNLKSQRHSGSFSDLAEAMWTSAKGDSTVWIASVRDEQSD

QVLVSELHAVGFSNVHLCGTAQKEDGLLNSYAEPSRMGADRWFAMLGARACKRGPLLVID

AGSAVTCDLVASDGRHLGGYIFPGPALMEAALQSNTQKVRYSDSLKLALDPGQSTAECVA

SGISVAMLGAIKQVCDQYPAHQVIFTGGAASGLNAVGLVGDWRPDLVLEGLLSRAHGTEV

AFAT

>WP_007230922.1

AAFHYFYMREVWVMSGDTPTDFRYIDWLLTVPLLMIEFYLILAAITKVAGGVFWRLLIGT

LVMLVPGYMGEAGYLNVTVGFGIGMLGWAFILYEIFFGEASKVAANEAPAAVQKAYTLMK

WTVTIGWSIYPLGYFFGYMAGGTDVGVLNIVYNLADVLNKIAFGLFIWYAANEDTSAKA

>WP_007230850.1

MKILLVEDDLATREEVSDLLETLGHHTVDSDCAEEALQYIRSDGSADLILFDLNMPGTSG

LDMIREVRHTSTQSISTMPAICMTGSRDAHSVVELLKTGITDFLFKPLRLADLKSSLQKV

ESEISRVRAQESQAAALNDKLNEKDKLLEELSLELSESQTESVLCLAYAAEYKDLGTGAH

LRRISKYAERMAELLGWSEERCSSIALAAPLHDVGKIGIPDTVLNKTGTLTSREFNCLKS

HTTLGAEILSASKSPVMRLGAKIAHYHHENYDGTGYPSGLVGSQIPIEAMITAIVDVYDA

LRTSRPYKEAMDHASAIDTMCNGDERTDVSKFHPELLKTFLHNHHDFGEIYRKNTVADVP

PIMLSAVAH

>WP_007230835.1

MSAEFDLKRSCFGDFMKILVVDDEPLIGSETSEYLSLHGYVSDHCHSCDAAMEILSSDPD

IRLVITDLRMPEKDGFTLIEATQPLQRHIEFIVVTGHGGKDEAINAVRTGVSEFLAKPVN

HFELLKAVKNAAQKIADHDHELSVKSSLQGKVFAGEKKIDRLLGNLDTSYAELTYCLATA

SEYKDPETGQHISRIGSYAALIAGLMGWTERKVEMIRLAAPLHDIGKIGTPDSILLKPGK

LGDVELRVMRTHSSIGHAILSQSTSPVLKMAANIALAHHERWDGSGYPNGLSAEEIPVEA

AITALADVYDALRSKRPYKPALDHRTTCDILLYGDGRTEAKHFSPELLQIFKENHDKFDE

IYESMHDQAVSC

>WP_007230577.1

LSLTGTDLEVELVGRVKLASAGDSGELTLPARKLVDICKSLPEGSEISFAAEDSKVTVKS

GRSRFTLSTLPAREFPNVEDSMGTHQFTIKQGQLKRLIDRTGFAMAQQDVRYYLNGMLWE

LKDKQLKVVATDGHRLALCTLPEKIEAGDDAQVILPRKGVLELARLLLAEDEDIAIVIGS

NHIRATTEEFTFTSKLVDGKFPDYQRVLPRSPNKIVLGSRLELRQAFTRTAILSNEKYRG

VRLKLTDNSLDIVANNPEQEEAEEAVPVDYQGESLEVGFNVSYLLDVLAVLSGEQIKLSL

SDPNSSALLEESDEGDSLYVVMPMRL

>WP_007229328.1

MDPIGTALVVDHDTARLEALASLVSSLGFTPENYTDANAAREYLSTRPDLDVILCEMDME

GLTWDSAHRSLQEMDVQIPVILFSDEAQASRMMRALRFGASDFFVRPVDDVEALQRSLDR

CVRQRQVRRELEQSRQRLQAANTELRGTIHVLEQDQQAGRQVQMRMLPATPLVLNDYVFS

HTVIPSLYLSGDFTDYFTVGDHFVTFFMADVSGHGSSSAFTTVLLKNLFARKRSDFLRQN

DDTILSPLALLKRANKEVMDLEVGKFATMVVGVLDMKSNNLRYSVAGHLPQPVLVSGDGA

RYLRGEGSAVGIMDDASYEEHMIDLPDSFMLALFSDGILEILPPKNLIEKEKYFLNVFEE

TANSPEEMVTRLGLDQADTAPDDIAALFISKRN

>WP_007228310.1

MSLCLQLDVGNSSAKWRLLEQGDVLSRGRYSAADANTQRELLESTASVDQIWVSSVAGGD

TEAELKEMLEQRWGVTPWFARTPAATGDLRNSYADPARMGVDRWLAMLGARARCGKRVCV

VDAGSALTIDLISATGQHEGGYIIPGPALMERALLLDTDRVRFTDEVSYDLAPGSSTAEA

VRHGIAVAQVGSVSIVLDGCASEPPALIFCGGAGQVLQQLLDRGGEFIPELVFEGLEIMA

AAP

>WP_007228237.1

MKNVLQHLTRIRDWWIALCTGRDPMRPLDEEHYRQRILAITSFFCLITVIGVPVVIPLVI

DISPQGRFAATTLLAIIGLSVLVSVLILRYLNNRIAALHLLLLVYTGAFAIACAYFGGTR

SPTFALLILAPVMASVVGGTGAGLFWTALVLIIWSVILGLERLGVQFTQIILPQNYNMAI

TLSYGAMGLSVISIIKVYAEMNKHLREALQGANSELEFLSNHDDLTGLYNRRFYEQRMAH

LLERAEITGKTIGLIMFDLDDFKQVNDTHGHGMGDALLKMLGERLRHQVRDIDLIARLGG

DEFVVLMENMRSSDDLPEIAAKLVAAVEQPVKARNEVMALKVSCGFALYPGDGLSRAELE

EKADKAMYRAKKRGSSPDPSLILH

>WP_007227932.1

MTARVLPFVLLLLAGCTTSPPSNVNNICEIFEEKSGWYGDAHDAKKEWGSPIPVMMAIMH

QESRFVAKAKPPRKKIFGFIPGPRPSDAYGYSQAKKSTWKDYKRGGGNYGADRDDFGDAI

DFIGWYNEQSKKRSGISKRDTYGLYLAYHEGHGGYNRRTYKSKKWLTDVARKVERRAGSY

QQQLSTCEKDLEKGGWFFGW

>WP_007226969.1

MILECDIGNTRCKWRVVGEGAEENRGAFDCADGFGELPSLDGIRRVKVSSVAGSTVNEEL

TRTLASAKLEIEFARTSPLKAGVENAYADASKLGVDRWVAMIAGYNRCRGPVLILDAGSA

LTVDLVAANGKHLGGYITPGIQLMKSSLLAETDGVRFDRDNHSSGTAFGTDTASAVHAGV

VAAQVGAAIVAIEEAGRKVSAGFAILLTGGDANVICTNLPATISAEVTMVPELVLDGLQW

VLP

>WP_007225387.1

MTTNLSASDPVGMSFWLISMAMVAATVFFLIERDRVSGKWKTSLTVAGLVTLIAAVHYFY

MRDVWVATGETPTVYRYIDWLLTVPLLIIEFYLILSAITKVPVGVFWRLLAGSLIMLGAG

FVGEVNPDYVVSGFVVGMLGWVWIMYEIFLGEASKINAASGNAIAQKAYGAMRLLVTVGW

AIYPIGYVLGYFTGSTDSATLNLWYNVADLWNKVAFGLVIWAAAVADSE

>WP_007225373.1

MNEIKCPHCKKAFTIDEAGYAEIVKQVHNSEFDQQLHERLELAERETRNAVKLAEEQARS

KLLEAESTKNDEIRKLQSELEAGDYARKLAVSKALKAVEKERDELANKLIQAKNDSTNAS

KLADANHSKKLEKTSAEKDAAIRLLEEKLSASEDTKNHAVTKAVNVAERERDELKSKIYR

TKLENEIAETSLKDNYEAQLKDRDHEIERLKDMKARLSTKMVGETLELHCETEFNLIRAT

AFPRAYFEKDNDSRTGSKGDYIFRDSDEHNTESVSIMFEMKNEIDETATKKKNEDFFKEL

DKDRNEKQCEYAILVSLLEPENSLYNSGIVDVSHRYQKMYVIRPQFFIPIITLLRNAAEK

SLKYKKELAVVKEQNVDITNFESELDEFRSGFARNYELASKKFKTAISEIDKTIDHLQKT

KDALLGSENNLRLANNKADDLTVKKLTKSNPTMEAKFKELNNDEDI

>WP_007225063.1

MNIKQWLNPFDLVVSVRTGFALIIIRRALLLFAVLSAPLAFSADISGVWKHSKNTAWIEI

SLADSSATVLRNDKFPERVGRTILKDLQVDTSTQGLWHGLIYVEKLGDYKDVEVSLPEAG

RMLLKGKVGFMTRTVEWLRVDNIR

>WP_007224727.1

MKLLLVEDDEALAKALLIALRNEGFSVDHVATGNEAIAHGKNNIADIIILDLGLPDIDGL

TVLKELRANKIVTPMLILTARDDLSDKITALDGGADDYLSKPFEIKELMARIRALGRRMN

SSISSVITAGRVSLDSANHHVEVDAIETPLSRREYTVLKALMENVGRIQTKAALENKLYS

WGEEISSNAIEVHISNLRKKMPEGFIKTVRGVGYTIDRSSS

>WP_157361222.1

MAALGVVAALALASCSAGDGGSGESATGDLRVWLVGTDTPQEARDYLIDTFESENPGSTL

TIEEQAWGGLVDLLTTNLSGSDSPDLVEVGNTQAAAFTSAGAFLDLTADYDALGGDDLLP

GFVEAGSYDGKFYAAPLYSGSRLVFYKKDALAAAGLSVPTTLDEYVSNGEALAEANPGAS

GIWWPGQDWYNALPFIWENGGEVATFESSGWKSQFSSPGSIAGLKQVQDVMTNASRAPKD

ANETNPQVGYCEGTTLQLSAPSWVKWSILAPLDAETPGCPDEEANLGVYAMPGKDGGAAQ

VFAGGSNIAVSAKSAHPELAKKALAIILSDGFQEIYGANGLVPAKLSLADTLGTDEVAAA

ISEAAGAARLTPPSPKWADVEASGALTDFFVQIAQGGDVASLAKDLDAKIDSILNG

>WP_157361184.1

MVQNRIGRAVLAAAAAVAVSIAMVGCAPASESEQGEVELRFSWWGTDSRHELTNEALDLF

EEKNPGITVVRDFGGFDGYIDKLLTQAAGNNSPDVFQLYEEVLREFASRGQLYDLNEATS

QGLSLDGWDQGLLDTSTIDGSLSALQFGLTTQAFIFNTELFDQAGVSIPTEGWSWDDLAT

AAKKVSDGTDSGTFGVTDLSTGYQVFEVWANQNGESYLTDDGLGFSAGTLEDFWNYWADL

RASGAATPGSLTSEYPTPFDAIIASKAASGFIFANQMAAVQSSIEAEIAVDRMPGESPEA

GSYLRTAMNIAIGSKTEHPKEAAMLVDFLLNDPEAYAILGIDRGVPANPAVGDAATANVD

DITAKGLTVIDGVREDGAAPPVPPKPGAGNVNALFAELAQEVQFDRMSIKDAVASFIERA

EQELS

>WP_156788299.1

MSRGILGPALVFMVMAIICLSLILPLRQLLIAGTLIVCTDLAVILLYFSGAFVINIDPVQ

AFAAPASWFSYSIVPIYASILIMLVLSRFNKGIEQVLTELEEEKSAAFYLSEHDHLTGLP

NMRVMEIRAHQAILMADRGESKPALLFIDLDHFKVINDKRGHDVGDLVLQEVAIRIQSVI

REGDIVARAGGDEFLVLLPQANDTVDAETVSQKICVTLANPFRLDDDEIYISASFGIAMW

PEHGRDLKSLTRSADQAMYAVKTSGKNGFKVSTSSIENG

>WP_156788178.1

MGDDAGNKTFGSLLKSELRDTDVIARTGGEEFALLIRTDSYSVTIIKAKMIC

>WP_157361215.1

MPVWFEVTSLSILLLILAADLIMAYKRPHIPSTRESALWVGFYVSLALIFALMMFLLGDV

EQAGQFIAGWVTEYSLSIDNLFVFVIIMARFSVPRKYQQEVLMVGILIALVLRGIFILLG

AQLIENFSFIFYIFGAFLLYTAIRQVFENHDDMEETESGIIRFLRKHINISPVFDGGKMR

TVIDGKKVFTPILVVFVALGITDLVFAIDSIPAIFGITTDPFIVFTANIFALMGLRQLYF

LLGDLIDKLEYLHYGIAFVLGFIGVKLFFHALHINELPFINGGEHVEWAPEIGTWTSLIV

ILVSMAVSVIASLVKMNVDKKRELVSIDSE

>WP_157361178.1

MLSSPTFCWVLSRPQDPCQGLPAVITAHYCCGVSDTNNTPDIVPFVARRLLTADQQRTAV

AVAAPVGAPSSASPTSLGVDPGSQQLTAQILHEFGPLAPYVGRPTITDVFVNGAQQVWVD

RGGGLEPVNDLGLTEPELRALAVRLISLGGRHIDEATPCVDVRLAGGVRVHAVLPPISAT

GTLLSIRIPSREPFGLAELDLAGFFTEVPMQRVKGLVDARENLLISGASGAGKTTFLGAL

LGAASETERIVAIEDVAELRVEHAHFVSLEARQANLEGTGSYGLPALVREALRMRPDRLV

LGECRGAEIRELLSALNTGHDGGAGTLHANSLRDVPSRLEALGALAGLDASAIARQAVSA

IGAVLHLDRVGGRRRLTQVGRLILDENERLAIADDE

>WP_157044971.1

MVTAAIGAGDPAQGRALLWLNVAALLLVLLSCFAVIHSTHATRELYTQLQVLESRQWHLQ

EDYGRLLLEESTWASHYRVEKVARTELGMAEPDLAHYKVVRR

>WP_156788350.1

MVLSSLNVARAEVAEIVAAPIEFPSTMAWSAYNLGTTGYNQAVAIGKVLKDHYDTNLRVL

PGKNDVSRLLPLQRGRVQFSANGAATYFAQEGVFQFAEKQWGPMPLRIVMASNGETNQAL

GVAADMGIATYSDLRGKRVPFVRGAPALNVTTEAYLACGGLTWDDVERVDFPGYSAMWTG

IVNDQVDAAYGTTVSGPTRKLEASPRGIFWPPAPHDDAECWARMAKIVPFFQPHMATRGA

AISIANPHEGATYPYPILITLAKTDPDLVYDLAKVIDIHYDEYKSADPGSIGWAMDRQVF

RWVVPFHGGAVRYFKSIGVWDDATQAHNDRLIGRQDVLAKAWRVHKASYPGKEGFADAWG

KARVQALDANGFDPVWR

>WP_156788318.1

MKVPRLLTKLILCALFTSLVAAGFATKNLRDFLNTPMDIQGEGLAYLLEKGGSLSQVGVD

LSLLGVLENRRWLSIYSRISGRGTAIEAGEYWLEPGLTPLELIAKFEQGDVRFFQLTLVE

GWDMSQVLSRLRSADALINTFGADTRVLTADMLGLETSFPSLEGLLFPDTYRYHSGTTDR

ELLLQAYQRMQKVLNDEWSDRSKNLPYDNMYQALIMASLVERETGVAWERAQISGVFVRR

LKLGMRLQTDPAVIYGLGASYTGNLRSRHLKDGSNKFNTYRHHGLTPTPIALAGREAIHA

ALHPADGKTLYFVAKGDGTHYFSETLKEHQKAVRKYQIEQRRKDYSSTPVIKPAG

>WP_007223442.1

MDLATVIGVVGALAIIITSMVLSGGIGMFTNMSAVLIVFVGSMFVVLSKFGMSQFLGAGK

VAAKAFFFKSTDPSAMIDEIVVLADAARKGGLLSLEGKEVGNDFLQGGIQLLVDGHDPDV

VKALLSKDKDKTVERHEQGASIFAALGDVAPAMGMIGTLVGLVAMLSNMDDPKSIGPAMA

VALLTTLYGAMLANMVAIPISDKLILRRGEEEMNKSLVIDALLAIQSGQNPRVIDSMLRN

YLPASQRPQADE

>WP_009774002.1

MVNAGIRIGVVGATGQVGAVVRRLLEERDFPVAEIRYFASSRSAGTTLPFKGEQITVEDA

STADPTGLDVAIFSAGATTSKAQAPRFAAAGVTVIDNSSGWRMDPDVPLVVSEVNPHAID

QAVKGIIANPNCTTMAAMPVLKVLDAEAGLERLIVSTYQAVSGAGLAGGEELLEQAAAAV

AQNTMGLVEDGAAVTMPAPNKFPKNIAFNVVPLAGSIVDDGLNETDEEKKLRNESRKILE

LPGLLVSGTCVRVPVFTGHSLSINVEFARPLSPARATELLATAPGVSLSDIPTPLDAAGA

DPSFVGRIRADEGVPDGRGLALFISNDNLRKGAALNAVQIAELVAAKITAKVSA

>WP_007235113.1

MSEAIDIAVVGATGAVGEAMMEILEQREFPVGKLYALASERSAGKTVRFRGKSITVSDLA

EFDFSKTAIALFSAGGSVSEEHAPRAAASGCVVIDNTSHFRRQEDIPLVVPEVNPGALAA

YRSTRIIANPNCSTIQMLVALKPIYDAVGISRINVATYQAVSGTGKAAIEELAGQTARLL

NGQPTEAKVYSKQIAFNALPHIDTFEENGYTREEMKMVWETQKILEDPDITVNATCVRVP

VFYGHSEAVHIETNTKITADAARKLLQDAPGVTLTDGREDGAYPTAVTDGAGSNPVYVGR

IREDISHPTGLNLWVVADNLRKGAALNSIQIAELLVKEHF

>WP_007227922.1

MSELYDIAVVGATGAVGETMISILEERDFPVGNLYPLASSRSAGKTIMFNGNTVKVTDLA

EFDFSQAQIGLFSAGGSISEKYAPIAAEAGCVVVDNTSHFRRDEDIPLIVPEVNIEALAG

YMTRGIIANPNCSTIQMLVALKPIYDAVGIERINVCTYQAVSGTGKEAIEELAGQTARLL

NGQEAQCEVYPKQIAFNVLPHIDSFQENGYTREEMKMVWETQKIFGDHSIQVNPTCVRVP

VFFGHSEALHIETVDKISAEQARELLQNAPGVQVMDEQADGGYPTAVGDSAGSDPVFVGR

IREDISHPRGLDMWVVSDNVRKGAALNSVQIAESLIATYLD

>WP_009773280.1

MPDTHSERVIEDEIVTDFSERMSYGSYLELDTLLSAQTPQSTPEHHDEMLFIIQHQTTEL

WLKLVIHELTSARDLIANDNLSIALKRIARVKHIQRTLTEQWSVLATLTPSEYSQFRDYL

GSSSGFQSYQYRAVEFLLGNKNAGMLKVFESHPEAHALLSKLLAEPSVYDEFLRYLSRHG

YDIPEAVLNRDVTRGYEQNDDLIETFRHIYDNESEHWLAYEACEEFVDLEDNFQLWRFRH

MKTVMRIIGMKRGTGGSSGVGFLQKALDLTFFPELFAVRTEIGRS

>WP_007236374.1

MDMTIVYGVGMFTAIVLALVMVILAARSRLVSSGNVSIEINGGKTIEVPAGGKLLQTLAD

ANLFLASACGGGGTCAQCKCQVSDGGGSMLPTEESHFTRRQANDGWRLSCQTPVKQDMRI

QIPEEVFGVKQWECTVESNDNVATFIKELVLRLPEGESVDFRAGGYVQLECPPHNVNFDN

FEIGEEYKGDWERFGFFKYGSASEDTTIRAYSMANYPEEKGIVKFNIRIATPPPGSEGIP

PGIMSSWVFDLKPGDKITVYGPFGEFFAKETDAEMVFIGGGAGMAPMRSHLFDQLKRLNS

KRKITFWYGARSLKEMFYVEDYDGLQAENENFTWHTALSDPQPEDNWDGLTGFIHNVLFE

EYLKNHPAPEDCEYYMCGPPMMNAAVIKMLVDLGVERDNIFLDDFGG

>WP_007228144.1

MNAVQETLADWRDSLIDLFPDARPVRWVLLALVAYLLIAIIVGMIWSLPPDHFDPSEKAA

EYAAQDGGEVVTGSTTTAALMGVMETLLEKPGGYLHNDRFPPGIWLDNMPNWEYGALVQV

RDLSRAMREVFSRSQSQSTEDKDLAMAEPRYHFDSDSWILPSSESEYRQAQDYTRGYFRR

LSDSTQAEAQFFARADNLRYWLSTVNTRLGSLSQRLSASVGQRRINTDLAGDAGASQSTA

APREMEVKTPWLEIDDVFYEARGTTWALIHFLKALEVDFADVLAKKNARVSLQQIIRELE

ASQETLWSPLILNGTGFGLVANHSLVMASYISRANAAIIDLRDLLLQG

>WP_007223884.1

MDWQAIKERLVVWREDRFDTATESNSTKVVLIVAAVYLLLAISVGMYWSMMPAQFPVQEN

AIVMAERSQQSVAVGSTTTAALIQVISTLLDKPGGFMSNDVMPPGLWLDNIKNWEYGALI

QSRDLTRALRESFSRSQSQSKEDLDLGKAEPSLNFSSDSWTLPASESEYKSAVKHLNRYL

VRLEDTGNSGSQFYARSDNLRYWLAIVESRLGSLSQRLSASVGKRRLNTDLAGDSAAQQS

TSAPSELEIKTPWTEIDNVYYESRGTSWALLHFLRAIEVDFHEVLKKKNALVSLQQIIRE

LEATQQTIFSPMILNGSGFGVLANHSLVMASYISRANTAIIELRDLLSQG

>WP_009772683.1

MSMFGLKESEIATLVRAVSVDGGGNLDRASIENRFARATWSGLAEPGDRLAGRAIQQLGS

ARSLTAVVEHWDAEQFATELSADGDPVSGDDMRQAIDRWMPRLKSDTALIALRQAARFGS

RLLIPDDSLWPERLHDLDWHAPSALWVRGTDAALAGIVDGIALVGARAATGYGEHITMEA

SAGLVDRGYTIVSGAAYGIDGMAHRAALASHGLTVAFLAGGVDRFYPSGHDSLLSRIVEN

GAVISELPCGSPPTKWRFLQRNRLIAAASIATIVLEAGWRSGSLNTAGHAAALGRPLGAV

PGPVTSAASAGCHRLIRDYDAVCVTNPDQMAELAPLDRAPETDATMTTPESQPPTNLPPE

SSAPKVDADSPPSTETTRLLDALSVRSARTADDIASRSGLALATVRAHLGLLELDGRVVE

SERGWKQASQSRTA

>WP_007234196.1

MSYVLARNAAADQLWFEFLATKMLDRASKRKLLAQGFTPPQLLEPHGTWPFDGAELRASL

RVSKSRQVARLKPLLACHLLHWGRQSSGCDVYPPLLSGLSDAPLALFVSGDINCLSRPAI

AIVGTRRPSRDGLKLADQMGYQLAAAGFLVVSGLARGIDAAAHRGALRSGGQTLAVMATG

MDRIYPSEHYRLAEEVAAQGALLSEFCPGVVPHRGHFPRRNRTLSGLCLATVVIEAGHPS

GSLITANAAVEQGREVFAAPWSLFHRGGAGCLRLLSQGAQLIDTPAAVIEHLAVHLSGWA

ELTADALDYSSNAGETVSLAPLEPAKRQLLTLLGDGEHDLASLASALQCSSRQLLAMVTQ

LELQGYVEQTSAGLRAVRHP

>WP_007224080.1

MKDDETRAWLALSRIPHLPRRVLHRLILATSSAEEIFQLSAFELTAAKVGAEAQKMLREG

VDLRQVEQDFKTVQLQHIKLLPVSSTLYPALLKEINDPPPLLYIRGDLSVLDLPSLAMVG

SRRSSQAGGANAFRFARELAGAGFSIVSGMALGVDTQCHRGALAAGGSTVAVLGTGIDIV

YPRRNKELFESIVCQGAVISEFPMGTDPHPARFPRRNRIISGMSLGVLVVEAALQSGSLI

TARCAMEQGREVFAIPGSIHNSGSKGCHQLIKQGAKLVESVSDVMEELKGWCADAPPVLE

EKGAQNKVSKDLHERERLLLDIIGYDPVSIDSLQQRTDWPMHDLVALVTALELRGLLDCV

AGSYQRTV

>WP_007236375.1

FLAVSKKIQAAFGLGVAVVVVLTITVPVNNLIYQYLLADGALAWAGLPDVDLSFLGLLSY

IGVIAALVQILEMFLDRYVPALYAALGVFLPLITVNCAILGASLLMVERDYTFGESAVFG

AGAGVGWALAIVALAGIREKLKYSDVPDGLQGLGITFITVGLMSLGFMSFGGIDI

>WP_009772673.1

MADFSLEDLKTLRERLGTGMVETKNALVEAGGDLEKATELLRLRGAKSNAKRSDRSTSEG

LIAAQSSGTSTTIIELACETDFVAKSDKFVALGEAVAAAVAAAGASTVEEGLAAPAGSST

VAQLIDDEAAILGEKFELRRLTKLEGDSFEVYMHRTNKDLPPQVGVVVAYSGDDAETARG

IAQHISFAAPTYLSREEVPADDVENERRIVEEISRGEGKPDAALPKIVEGRLGAFFKQVA

LLDQDYARDNKVSIAKVSADAGITVTGFARFKVGA

>WP_148224520.1

MMSVRWTMPLLIAAVLMSSFAIIHSTHASRAYYANLQRLEGTHWYLQEDYSRLMLERSTL

ASPHRIAKMAQDELIMRAPDLATYRTIVEGAY

>WP_148224355.1

MIVILSGIVICFTLGKQALHAPMNLPQPDATVIVEQGDSLKQILTKLKSRGFIESSRLLE

LWARWQGVDRQIHTGEYLLVPGLSGIGFLERLGRGDVLSYKITLPEGITLQQALQRLHDD

RRLVRELRDAHDPLLLELVSPMTSPEGWFLPETYRFVAGDSDYDILRRAHHLMQRELIRV

WEARSSDTPLMTPYEALTLASIVERETSVAKERATIAGVFSRRLQAGMRLQTDPTVIYGL

GSDFDGNLKRRHLKDAANPWNTYRIKGLPPTPIALPGVAALEAAVRPASGAALYFVARGD

GYHVFSETIEEHNAHVQRYQLSRKVDYRSTPKGGD

>WP_007229556.1

MSNQQLGDFLAHSVELESEARERYLELAQAMIAHHNTDVAGFFNRMAEESRLHLEEVAEI

AQDIELPGLKAWEFGWPEEESPEAVSYEAVHYRMSLRQAMLLALENERAAEKFYRSFADA

SSDGETRHLAAQFSAEEASHAAQLEKMLGKLPPDREHHLEEDDQPHMPE

>WP_007234052.1

MSKTNIFVGVFLSLLLGAASQAQSSDLTNPTALVDNGPYTGDLQGLVERRIVRVLTVYGP

GRYYLDNGAKGVTAEYANRLEKVINESFDTGHLKVAVFVLPVARDELFLALEQGRGDIII

AGTTITPAREQRAAFTIPSSKPLKEILVMGPSAPPINSIDDLSGKSVYLRASSSYSDSIA

TLNERFTREGKALVTVEPMSELLEDEDLIEMVDAGLLPWTIVDDYKPTQWSGVFTNLTVR

NDIIFRKGSRHAWAVRQDNPELKKFLNNFLKDNKEGTLFGNILKNRYVRDFNWAANTNAE

SELQRYRDLEALFRRHGVSYGIDPALLAAQGFQESRLDQNVRSGAGAVGVMQLLPSTAAD

KNVGIPNIHEVDPNIEAGAKYLAFLKRRYFSTPGMDPLNGALLALAAYNAGPAKVRRLQE

TARTRGYDPYRWFDNVEVIAAEKIGRETVQYVANIFKYYLSYQMINRESARREAARQAAG

APTHEQRQN

>WP_007235497.1

MIKPRGRRIGLIRLSAIGDVCHAVATVQALQRHAPEDDITWIIGRTEAALVSDLPGITFI

VFDKKQGLTAFRNVLNEIAEPFDVLLHMQVSLRANILAAVVPAKAKLGFPKHLSKELHGM

VVNRRVPMPETPHVLEGFQHFAYALDVPTFAPTWSIPISEADQAWVRERLTAQKPYVVIA

PSASNAERNWLVDRYAALANHLQYRGYNVVLTASPAPSEVALAQQITALAGSNIINLAGQ

TTLKQLLAVVADATLVVAPDSGTAHMAVTQNTPVIGLYAHSNPNRTGPYRFQFLTIDAYQ

KNLQHLFSNSAKSNKWGVRLKGAHLMEDIALSEVIAKADEVLSEAPNPSDHNS

>WP_007235565.1

MFKIRTFNAISVKGLERFPRQSYEVGGEIGSADAMLLRSHKLQADEISASVTAIARAGAG

VNNIPLSHCTELGIPVFNTPGANANAVKELVAAGLLLASRDILGGIDFVNSLSEDLDEQA

MGPLLEAEKKRFAGAELKGKTLGVLGLGAIGSLVAQLGLELGMDVVGFDPAISIEAAWQL

PSSVKRMENMQALFSRADYISIHVPAIESTHHLINQETLKYFRSDACLLNFAREQIVDTE

AVAAALDKQGLGRYITDFPHPLLRGRKDCILMPHIGASTAEAEENCAIMGADQLRAFLEH

GNIRNSVNFPRLELERTTGSRIAITNTNLPGTLSHILTAIGDSQINVVDLLNKSRDEIAY

NLIDLNTTPPADLLEQLRGIEGVINVRCIPDQAAD

>WP_007227712.1

MAKQVLTLNQISLKGLERLPRDSYEIASEFSHPDAILLRSHKLQAQDIADSVLAIGRAGA

GVNNIPVAECSQRGIPVFNSPGANANAVKELVAAGLLLGSRGIVEGIQYVDTLSAMADKT

EMNKTLEAQKKQFKGSELEGKTLGVVGLGAIGSMVAEMALTMGMDVVGYDPALSVEAAWR

LSSQVRKADTLSALFGRCDFITLHLPVLDSTRGLINAELLSSTREGTCLLNFARQEIVDE

EALVQALDGDKLRKYIADFPSPALIGRDNVILMPHIGASTDEAEDNCAIMAANQLKDFLE

NGNIRNSVNFPNLSLERVSGCRLSVTNENVPKILGSVLSILADENINVIDMLNKSRNDIA

YNLIDVVGHTSDEVLDKMRALEGVVNVRMIGDCA

>WP_007226314.1

MSSISKTVAKTFGLILFFISIANAEITGIVVSVTDGDTIKVLDENSNQHKVRLTGIDAPE

RGQPFGQASKKYLASMVSGKEVFVESNKKDRYGRVLGKVWVQPADCPSCGKTLDINHAQL

LAGMAWWYRYYAKQQSPEDRGRYESAEDEAKARGWGLWSAASPINPYNWRKGRR

>WP_007229969.1

MSATKPDLVWDRIRTETQKHAQEEPVLASFLHSTILNHDSLECALSFHLASQLDSPTVTS

LLLREVMLQAMRADDAIGEAIRADLLAVVERDSASHELYIPFLYFKGFHALQSHRIAHWL

WHNNRKSMGLFFQNRISVEFGVDIHPAAKMGQGIMLDHATGLVIGETAVVGNNVSILQSV

TLGGTGKQDGDRHPKIGDGVLISAGAKILGNICVGDGAKVGAGSVVLEDVPPHTTVAGVP

AKVVGRPATNAPALDMNHDFFCDSGDVEG

>WP_007228043.1

MEELLSLFIRSIFIDNMALAFFLGMCTFLAISKKIDAALGLGIAVIVVLTITVPVNYLIY

NYLLADGALAWAGQPDLDLSFLGLLSYIGVIAAIVQIMEMFLDKFVPALYNALGVFLPLI

TVNCAILGATLFMVERDLDFAESVVFGAGSGVGWALAIVALAGIREKLKYSDVPDGLKGL

GITFIIVGLMSLGFMSFGGIDL

>WP_007227945.1

MDMTIVFGVAMFTAIVLALVAIILFARSALVSSGNVSIEINGEKTITVPAGGKLLQTLSE

SGLFLPSACGGGGTCAQCKCIINDGGGSMLPTEEGHFTKRDAAEGWRLSCQTAVKQDMKI

EVPEEVFGVKQWECTVESNPNVATFIKELTLKLPEGEHVDFRAGGYVQLECPAHHVKYSD

FDIEEEYRGDWEHFNFFKHESVVKEDVIRAYSMANYPEEKGVVKFNIRIATPPPGSEGIP

AGQMSSWVFNLKPGDKVKVYGPFGEFFAKDTDAEMVFIGGGAGMAPMRSHLFDQLKRVHS

DRKISFWYGARSLREMFYVEDYDMLARDNENFDWHVALSDPQPEDHWDGLTGFIHNVLFE

EYLKNHPAPEDCEYYMCGPPMMNAAVIQMLIDLGVEPENIMLDDFGG

>WP_050774034.1

MEVIVGEPRKPLPRGSDFYTPEQRLRRDSSVWTTVQGVLAPLQFLAFALSLVFVINFLAN

GTGYSAAVISVLIKTLFLFTIMVTGAIWEKVVFGRYLFAPAFFWEDVVSMLVIFLHVAYV

VSWLFDLQAPREQMWLAIAAYTAYVINAAQFLLKLRAARVGSSQNNTDSVNEYAVEVSR

>WP_040823654.1

MLLAGVDEVGRGPLAGDVVAAAVILDPANPIRGLDDSKKLTEKKREALFPEIQEKALSWF

VARASVREIDELNILQASLLAMKRAVEGLVLQPEHVLVDGNKLPRWAYSAEAVVRGDSRV

QVIGAASILAKVVRDREMVAFDDEYPGYGFAGHKGYPTRVHMTALDVLGVTPIHRSSFGP

VKRKIAQMNRP

>WP_009772453.1

MATPNPLDAVINLAKRRGFVFQSGEIYGGSRSAWDYGPLGMALKENIKKQWWQTIVQGRD

DVVGIDSAVILPRKVWEASGHVEVFSDPLVESLHTHKRYRADHLLEAYEEKHGHPPVNGL

ADIRDPDTGQPGSWTEPQNFSGLLKTFLGPVDNEEGMHYLRPETAQGIFTNFANVMGAAR

MKPPFGIGQVGKSFRNEITPGNFIFRTREFEQMEMEFFVEPGTDEEWHQYWIDESMKWYT

DLGIKPENLRFYEHAQEKLSHYSKRTVDVEYRFRFAGSEWGELMGIANRTDFDLRTHSEA

SGADLSYFDQAKDERWTPYVIEPAFGLTRALMAFLIDAYAEDEAPNAKGGVDKRTVLRLD

RRLSPVKVAVLPLSRNERLSPLARSVAADLRKFWNVDFDDAGAIGRRYRRQDEIGTPFCV

TIDFDSLDDNAVTVRERDTMEQKRMPLEELRGYLAQELIGC

>WP_007236092.1

MSEDRTVWVVDDDRSIRWVMEKALTQAGLLCQSFETAEALLEAITSGAPDVVISDIRMPG

IDGLALLGQLRAAYPELPVIITTAHSDLDSAVASYEEGAFEYLPKPFDVDEIVATVLRTP

TMRKERKAPVTELPDKPTEIIGNAPAMQEVFRAIGRLAHSQITVLINGESGTGKELVARA

LHRHSPRKDGPFIALNMAAIPRELMESELFGHEKGSFTGATARRAGRFEQADSGTLFLDE

IGDMPAETQTRLLRVLADGEFFRVGGAAPVKADVRIIAATHQNLETLVANGQFREDLFHR

LNVIRIHLPRLADRQEDIPKLMQFFLGKAAQELGVEGKVLSTSASRYLCQLPWPGNVRQL

ENTCRWLTVMAAGREIHPSDLPPELLEPAQSQRVDNATTWQDTLATWAQQRLAAGESNVL

RKALPEFERIMIAAALTHTGGKRAEAAETLGWGRNTLTRKIKELEEDGTPAKGA

>WP_007226588.1

MATYRNFALVAAIVIVAFYQTLVDLMGNWLKFDESQSHGLIIIALFIHLFTGQLKQLPSP

PATPNWLGLMGLSASSLVWCLAAMLNIEAIEQLILLPILFFLCWSSLGLRSTVTLTPSIA

LLIFAIPIWDYLTPTLIDASSYVVMTLIQLSSITAFIDGNSIYLPHGRIDIADGCSGLRY

FIIAIALAYYLILTSKTTHLTKVKVLGIAIALGLFTNWLRIYIIIMVAHFTEMESSLVKD

HELFGWFLFFIVCLPLVYFARSLPHYEPTTPSATSAGVTKLTLVVSVIALTSGPLLYQLM

NTKVTAPNLGNWQQLGYQQLSSPTNGPFQLPPSNLNLRKQSGATLRDVAIHWQNSQDSDL

VPYIANSLNRDYWTQLQTSTLQTPKQQSLQLNLYNRKATNQYRCTVSWYRVGGMETTHYN

IAKLLQIPALLSQHNQFSAAVISINSETANCDPHQQQLIDAAIETHNDIVQLTGLTEQ

## Testing dataset

**Bacteriocin**

**----------------------------------------**

>BAC005

TPVVNPPFLQQT

>BAC008

DIDITGCSACKYAAG

>BAC013

CVQSCSFGPLTWSCDGNTK

>BAC052

MNFLKNGIAKWMTGAELQAYKKKYGCLPWEKISC

>BAC076

ATYYGNGLYCNKQKCWVDWNKASREIGKIIVNGNVQHGPWAPR

>BAC110

GGAPATSANAAGAAAIVGALAGIPGGPLGVVVGAVSAGLTTAIGSTVGSGSASSSAGGGS

>BAC112

NKWGNAVIGAATGATRGVSWCRGFGPWGMTACALGGAAIGGYLGYKSN

>BAC116

KSYGNGVQCNKKKCWVDWGSAISTIGNNSAANWATGGAAGWKS

>BAC127

AVNDYEPGSMVITHVQGGGRDIIQYIPARSSYGTPPFVPPGPSPYVGTGMQEYRKLRSTL

DKSHSELKKNLKNETLKEVDELKSEAGLPGKAVSANDIRDEKSIVDALMDAKAKSLKAIE

DRPANLYTASDFPQKSESMYQSQLLASRKFYGEFLDRHMSELAKAYSADIYKAQIAILKQ

TSQELENKARSLEAEAQRAAAEVEADYKARKANVEKKVQSELDQAGNALPQLTNPTPEQW

LERATQLVTQAIANK

>BAC149

MSKRDCNLMKACCAGQAVTYAIHSLLNRLGGDSSDPAGCNDIVRKYCK

>BAC177

DWTCWSCLVCAACSVELLNLVTAATGASTAS

>BAC213

KCPWWNLSCHLGNDGKICTYSHECTAGCNA

>BAC215

VLSIVACSSGCGSGKTAASCVETCGNRCFTNVGSLC

>BAC217

GWVACVGACGTVCLASGGVGTEFAAASYFL

>BAC220

MGAIAKLVAKFGWPFIKKFYKQIMQFIGQGWTIDQIEKWLKRH

>BAC221

KRKKHRCRVYNNGMPTGMYRWC

>BAC226

ITSFIGCTPGCGKTGSFNSFCC

>BAC227

ANLGNYTSQCYSSQCYSSKCYSDSCYSSNCYTGRHMCGYTHGYSC

>BAC228

GIGTAQCAYFKALCYSGGSEWLGGYGGCGSTQNNCELARKYC

>ACA04496.1

MNKKNILPQLGQPVIRLTAGQLSSQLAELSEEALGGVDASTSIAPFCSYDGVDASTSIAP

FCSYDGVDASTSIAPFCSYDD

>CAA74348.1

MENKKDLFDLEIKKDNMENNNELEAQSLGPAIKATRQVCPKATRFVTVSCKKSDCQ

>AAK32694.1

MNKELNALTNPIDEKELEQILGGGNGVIKTISHECHMNTWQFIFTCCS

>sp|Q38L35|Q38L35_STRSL

MKNSKDVLNNAIEEVSEKELMEVAGGKKGPGWIATITDDCPNSIFVCC

>AAZ76602.1

MKSNLLKINNVTEVEKDMVTLIKDEDMELAGGSTPACAIGVVGITVAVTGISTACTSRCI

NK

>sp|Q52052|Q52052_9ZZZZ

MENLSVVPSFEELSVEEMEAIQGSGDVQAETTPVCAVAATAAASSAACGWVGGGIFTGVT

VVVSLKHC

>BAD74571.1

MAKLDDFDLDIVVKKQDNIVQPNITSKSLCTPGCITGILMCLTQNSCVSCNSCIRC

>BAB04172.1

MVNSKDLRNPEFRKAQGLQFVDEVNEKELSSLAGSGDVHAQTTWPCATVGVSVALCPTTK

CTSQC

>NP_940772.1

MENSKVMKDIEVANLLEEVQEDELNEVLGAKKKSGVIPTVSHDCHMNSFQFVFTCCS

>ABI99444.1

MNKKNILPQQGQPVIRLTAGQLSSQLAELSEEALGDAGLEASVAACITFCAYDGVEPSCT

LCCTLCAYDGE

>WP_013079673.1

MTSRFQLLRLGKADRLTRGALVGLLLEDITVARYDPM

>YP_142020.1

MATQTIENFNTLDLETLASVEGGGCSWRGAGGATVQGAIGGAFGGNVVLPVVGSVPGYLA

GGVLGGAGGTVAYGATCWWS

>NP_297556.1

MRELTSIEMNNVSGGDLATRIEASIVFGVSAFFAGSIWGGTRGGDGGGILGVGSIAQGVG

MVYGGIVGGIGGLIAGFVLDKNVTYNYAVGFYNSLFNGTFTK

>CAA11804.1

MKNLKEGSYTAVNTDELKSINGGTKYYGNGVYCNSKKCWVDWGQASGCIGQTVVGGWLGG

AIPGKC

>AAL39164.1

MKQYKVLNEKEMKKTIGGESVFSKIGNAVGPAAYWILKGLGNMSDVNQADRINRKKH

>AAU29394.1

ATRSYGNGVYCNDDKCWVNWNEANQQIAGIVISGWASGLAGMGH

>NP_964622.1

MKQFNYLSHKDLAVVVGGRNNWQTNVGGAVGSAMIGATVGGTICGPACAVAGAHYLPILW

TAVTAATGGFGKIRK

>AAL77872.1

MKKFKELKENELTAITGGSFVGYYLGRFLASATHYYGKTVTKGHMHSSTINN

>NP_664144.1

MTTMKELTINDMASISGGNAPGDAVIGGLGGLASGLKFCKLPHPVLTGGCVVGFTVGGAY

LGYTAN

>AAL09346.1

MMKKIEKLTEKEMANIIGGKYYGNGVTCGKHSCSVNWGQAFSCSVSHLANFGHGKC

>AAZ76605.1

MWGRILAFVAKYGTKAVQWAWKNKWFLLSLGEAVFDYIRSIWGG

>CAA75396.1

MDKIIKFQGISDDQLNAVIGGKKKKQSWYAAAGDAIVSFGEGFLNAW

>CAA75397.1

MNNALSFEQQFTDFSTLSDSELESVEGGRNKLAYNMGHYAGKATIFGLAAWALLA

>AAG02567.1

MKISKIEAQARKDFFKKIDTNSNLLNVNGAKCKWWNISCDLGNNGHVCTLSHECQVSCN

>YP_395172.1

MMIFKKLSEKELQKISGGVGIQKCSLGFSSREYLNKITKWIKHH

>ZP_03845684.1

MLYKIIYRSMILMEKFIELSLKEVTAITGGKYYGNGVHCGKYSCTVDWGTAIGNIGNNAA

ANWATGGNAGWNK

>AAY44084.1

MQNTKELSVVELQQILGGKRASFGKCVVGAWGAGAAGLGAGVSGGLWGMAAGGIGRELAY

MGANGCL

>YP_025353.1

MSGGDGKGHNSGAHDSGGSINGTSGKGGPSSGGASDNSGWSSENNPWGGGNSGMIGGSQG

GNGANHGGENTSSNYGKDVSRQIGDAIARKEGINPKIFTGYFIRSDGYLIGITPLVSGDA

FGVNLGLFNNNQNSSSENKGWNGRNGDGIKNSSQGGWKIKTNELTSNQVAAAKSVPEPKN

SKYYKSMREASDEVINSNLNQGHGVGEAARAERDYREKVKNAINDNSPNVLQDAIKFTAD

FYKEVFNAYGEKAEKLAKLLADQAKGKKIRNVEDALKSYEKHKANINKKINAKDREAIAK

ALESMDVEKAAKNISKFSKGLGWVGPAIDITDWFTELYKAVKTDNWRSLYVKTETIAVGL

AATHVTALAFSAVLGGPIGILGYGLIMAGVGALVNETIVDEANKVIGI

>AAN76832.1

METAVAYYKDGVPYDDKGQVIITLLNGTPDGSGSGGGGGKGGSKSESSAAIHATAKWSTA

QLKKTQAEQAARAKAAAEAQAKAKANRDALTQRLKDIVNEALRHNASRTPSATELAHANN

AAMQAEAERLRLAKAEEKARKEAEAAEKAFQEAEQRRKEIEREKAETERQLKLAEAEEKR

LAALSEEAKAVEIAQKKLSAAQSEVVKMDGEIKTLNSRLSSSIHARDAEMKTLAGKRNEL

AQASAKYKELDELVKKLSPRANDPLQNRPFFEATRRRVGAGKIREEKQKQVTASETRINR

INADITQIQKAISQVSNNRNAGIARVHEAEENLKKAQNNLLNSQIKDAVDATVSFYQTLT

EKYGEKYSKMAQELADKSKGKKIGNVNEALAAFEKYKDVLNKKFSKADRDAIFNALASVK

YDDWAKHLDQFAKYLKITGHVSFGYDVVSDILKIKDTGDWKPLFLTLEKKAADAGVSYVV

ALLFSLLAGTTLGIWGIAIVTGILCSYIDKNKLNTINEVLGI

>CAA33859.1

RFAHDPMAGGHRMWQMAGLKAQRAQTDVNNKQAAFDAAAKEKADADAALSTAMESRKKKE

DNKRDAEGKLNDELAKNKGKIPGLKIDQKIRGQMPERGWTEDDIKNTVSNGATGTSFDKR

SPKKTPPDYLGRNDPATVYGSPGKYVVVNDRTGEVTQISDKTDPGWVDDSRIQWGNKNDQ

>prf||1615299A

RFAHDPMAGGHRMWQMAGLKAQRAQTDVNNKQAAFDAAAKEKSDADAALSSAMESRKKKE

DKKRSAENKLNEEKNKPRKGVKDYGHDYHPDPKTEDIKGLGELKEGKPKTPKQGGGGKRA

RWYGDKGRKIYEWDSQHGELEGYRASDGQHLGSFEPKTGNQLKGPDPKRNIKKYL

>prf||1814449A

MSGGDGRGHNSGAHNTGGNINGGPTGLGGNGGASDGSGWSSENNPWGGGSGSGVHWGGGS

GHGNGGGNSNSGGGSNSSVAAPMAFGFPALAAPGAGTLGISVSGEALSAAIADIFAALKG

PFKFSAWGIALYGILPSEIAKDDPNMMSKIVTSLPAETVTNVQVSTLPLDQATVSVTKRV

TDVVKDTRQHIAVVAGVPMSVPVVNAKPTRTPGVFHASFPGVPSLTVSTVKGLPVSTTLP

RGITEDKGRTAVPAGFTFGGGSHEAVIRFPKESGQKPVYVSVTDVLTPAQVKQRQDEEKR

LQQEWNDAHPVEVAERNYEQARAELNQANKDVARNQERQAKAVQVYNSRKSELDAANKTL

ADAKAFIKQFERFAREPMAAGHRMWQMAGLKAQRAQTDVNNKKAAFDAAAKEKSDADVAL

SSALERRKQKENKEKDAKAKLDKESKRNKPGKATGKGKPVNNKWLNNAGKDLGSPVPDRI

ANKLRDKEFKSFDDFRKKFWEEVSKDPELSKQFSRNNNDRMKVGKAPKTRTQDVSGKRTS

FELHHEKPISQNGGVYDMDNISVVTPKRHIDIHRGK

>YP_194414.1

MVGSITPKLVYRLNGMHHVVAQVGAVNGDHVFALQLLHSAHDVLVYRKHKGLTKDINYTN

PHLVMTGFGHTQTWVPANDNDEYFVGAKPNSGNWTTQIARVKYPRLLSENYTSNTQLPRL

SHLNRVTDVPYDGHNHLHRVEASVSPNGKYFMIASIWNNGSGHFGLFDLDEVNQKLDENG

TTNTPITDLHCLSAFHIDNFDNPSVAPDEEEPTMIDSVQGYAIDNDKNIYISNQLSPKIN

HETGEVTTWARKIVKFPWGETDSNNWQVAMIDGIDLPDRYSEVESIHVNAPDDIYLTVAY

HQKIVKGDEYALRTLENQIFHIDNL

>AAT85003.1

MEMVDQKINAQVLSGVNDDISEMKSLTTLRKRVVTDGEVVSKSQNAFRLAGGKTGVILRN

DGNDFYALVTPEDQAQDGQWNTLRPLSFNLKTGRVSLRNGVDISGGAVVSHDAGISARTT

GPSPIINGQTYSSPSIHTDFTSGNITTQMMMCARVEAGKQDYGLLSYRDWQGSWNELRVR

SNAELDAGQFTKRNSEGWIKAAGNRNVNNDKDRKTNALWIQGAGDLSADFYHYERIGQHH

FLGLHVANGGAQGWYEFRNDGHAYTNGAWNSSSDARMKTQVEKIDNALEKLDCISGYTYL

KQGVTEAGVIAQELEEVLPQAVSKTELTLNDGSVLKDARSININGVVALLIEALKEERQA

RLALEKRLADLEARSGQETE

>AAT90328.1

MAEQKKNALVLNGINDDITELKSLTTLRKRVVSDGEIVSKGVNGFRLAGASTGVILRNDG

KNFNFLTTADGQARDGAFNTLRPFAFSLTTGRVSLRNGVDVSGGAFISHNAGITAQTTGP

DPLINGQTYRAPDIHTDFTTGKKTTTMLMGSRIVTGQEDYGLISYRDMKGSWNELHLKPN

AELSVGQLTKRNTEGWYKAAGVRKVNNGKDNKTNALWIQGAGDLSADFYHYERIGQHHLL

GLHVANGGAQGWYEFRNDGHAYTNGAWNSSSDARMKTDIEKIDNALDRLDRIGGYTYLKQ

GKPEAGVIAQEVETVLPQAVTQTALTLNDGSVLEDARAVNINGVVALLVEALREEKQARL

ALEARLQVLEGTDAVHS

>ZP_00378412.1

MNNLYRDLAPVTDSAWAEIEEEARRTFKRNIAGRRIVDVEGPTGFETSSVGTGHIRTLGS

TGGDISIKQRISQEFIELRVPFTVTRQAIDDVERGSGDSDWQPVKDAATTIAMAEDSAIL

HGLDSAGIGGIVPGSSNTPVAIPDAVEDFADSVAQALSGLRKAGVDGPYSLLLSSEEYTK

VSESTDHGYPVRDHLSRLLGDGEIIWAPALEGALLVSVRGGDYELHLGQDLSIGYHSHNG

DSVELYLQETFGFLALTDESSVPLHR

>YP_121242.1

MNNLHRELAPITSEAWAAIEEEAGRTFKRHIAGRRVVDVAGPHGVDFSAVGLGRTTGIAA

PDEGVQARQRVVAPLVELRVPFTLSREELDNVERGAKDTDLDAVKEAARRIAFAEDRAIF

EGYPAAGITGIRAAGSNAPITVPDDARLVPEAITQALTALRLAGVDGPYSVLLSAELYTE

VSETSDHGYPIRTHIERLIPDGEIIWAPAIDGAFVLTTRGGDYELTLGQDVSIGYLSHDA

DTVRLYFQQTMQFLVHTAEAAVALRR

**Non-bacteriocin**

**-------------------------------------------------------------------**

>WP_177374305.1

MPLDPIHAFYCRKDYLSLAQSCKVKSGGICARCGGVFDLNELRPHHKIELTLDNIDDTNI

TLNPDNIEVLCHACHNAVHSRFGNAIGAKRVYLVYGSPYAGKTTYVALVATRNDIVVDLE

RIHAAICVCGQYDKPDATKRIAFNIRDYLLDEIRTATPRRKWQDAYIIGSYPDRIDRDNF

VREYNAELVHIDTPQDACVKRAYEDIKRVAARDAVVGWIADYWRRYNE

>WP_142482129.1

MIEVSTRQDRANFYGSNTWRKLRLKALERDHYECQWCKEQGKVTTINDAILEVDHIKELE

HYPELATDMDNLRTLCKDCHNKRHGRMNYRGEERKKKFDDEWW

>WP_149877315.1

MTELVVVLFYWGDSMLVACSRCGVIHERGDCKIQDGYSERRIKKRGEVERFRSSALWQRK

RKKILDRDKHLCRVCLDGKYVPKAITNQRLEVHHIVPIVENEKLKLADDNLISICAFCHV

LAEKGNVPRDYLFGLVKIPPRGHYVEN

>WP_121704945.1

MVYIRKRHWVTYNSEKCKMYLRNDFQFECAYCGMKERDNVIGEGLFEKDHFVSRQSDVAW

NLDSYGNMVYSCCKCNGTKSDQNIEIILDPCKDDIYGGQHPHIRRLGAENHYKLYGVTPQ

GQQFIDDLKLNSRFYRKMRQTQAQNEEIRREIYQLLDKSSDFQPSGIDRKIEAYLENGTL

IDERSDEFRCGTSKAGEDVYRVLEKLKERDIKYELLFADDDLDVRVEYCGNIYDCEIRVT

DYAGTEKRGPIVKREKKKTWLKTGNVCGVLYYYKEQDIMDLYIYPNEERTEIVKLG

>WP_086414226.1

MSKEKTASRWGGKGVRVGIIACVIVMVAGIVLWMVQLTGGMIQTGMRNLDAWGLYLTLFM

FFVGLSAGGLIISSIPNAFGMKGFGDISKVAIWSSVCCTCMAIGFVVVDLGGPLRLWELF

VYSNLSSPLMWDILVLSIYLVLSLVYLWAYVRYEQGRMKHTGIRFVSAVALIVAILVHSV

TAWIFSLSPAHEFWHTALMAPWFVASALDCGTALVLIVVIVLRKVGYLELDQHNIVNLAK

MLAVFVCVDLYFFACDLLTSGYFGGTDGAEVVATLTTGSIAPFFWIQMAFMALALVILFV

PKLRTNGGVVVASALVIAGVFCKRCQIMLGGFQIANIDFADTANAFTITNWTDGYSLAGY

SGLVYWPEPIEFGVSLGVIALGALFLLLGLRYLPLRQAKRVSE

>WP_160213184.1

MYGPLIIAYLFFGGTAAGAMLVMAWWSLRFYRKANRPTSRMARAFAAMQQRVYPIGFVLL

LVSMLCLLGDMNYLERAFLVFTRPHPTPITFGAYALAAEMVLAAALSVANILQPLFFTGK

VRRFLEILTVPCSVLLMVYTGVYLFSIMGVPLWNNPAIIPLFFCSSLSSGISAVLLVDYF

ADGSTLLLRAAKPLQKAHMTCIAAEAIVAIAYGASLALDPAAEASLSLLTSPGIAPVLLI

GFAGFGMAVPFCMEGYTLARKECRTIPVSDFVCLVGGFCLRWCVIMCATH

>WP_152931844.1

MLAYLIGLVVTSTLIFIFSEEKVTYRLFAAAITGLTWPLSLIPSIISLMIRKSD

>WP_099730807.1

MIFAPVIRRAAYAQAPRSADLALQRFLMGALAQPAAAPAAGCTVTQDEKATTLQLDVPGL

AREQLSISIEGQVVQVQSVEGAPRKVQRAWELPTEIDASASTAKLENGVLTLTLVRLEPV

SKATTLTIH

>WP_048781921.1

MKASTLRKERKAQSIKSEIIGEVLNAVTHGIGVALAITALVLLLMKAVAVNNTTQIIAFS

VYGASLILLFLASTLYHSFKFTKAAKVFQRIDHSSIYLLIAGTYTPFCLIGIGGQQGFIF

CIAIWVFAIGGVIIEAFFLEKFSKISVFLYLAMGWVSIFTLKPLYESMGWGGILYLFLGG

LSYSLGTIFYKRKYHNFYHVVWHLFVLAGAIFMFLAVFKYL

>WP_005865615.1

MLTAMVIVFLVGYLMIALEHPLKINKAGTALLIGTILWVMYTYAAPFFIPRASAEEFSLF

LESFPSLGSLTFKEQCTRFVVEHQVLDSIGEIAETLIFLIGAMITVELIDAHGGFMFITN

HITTKKKKKLLALIAVITFFMSAVLDNLTTSIVMIMLIRKLLGNYKERWVFGSIIIIAAN

SGGAWSPIGDVTTIMLWVRGNISTSSTIPHLILPSIVSALIPVLIAMRFLHGNVTPPNAF

SQMEADNELLKKLKDKEKLSILIIGVLCLLFVPVFKTVTHLPPFMGILMGVGILWFYTEM

LYARKPIDEDLKLRLSKVVHRIDGATLLFFLGILLAVDALRCSGVLSDFAFWLDDTVGNV

YAVNLIIGALSSIVDNVPLVAGAIGMYPVATDAMVAAATDPAYLANFMQDGVFWQFLAYC

AGVGGSMLIIGSAAGVVVMGLERINFIWYLKNISLLALAGYLSGAVVYILQNLIL

>WP_169170392.1

MIFAPVVRRAAYATRLPMSDLALQRFLRAALARPAAAPGCSAAQDEKAITLQLDVPGLAR

EQLDITIDGAVVRVRSVDGAPRQVQRAWELPEAIDAAASGAKLEHGVLTLTLAKLAPVNR

ATHLTIQ

>WP_110511594.1

MAHYISLFVRAVFVENMALAFFLGMCTFLAVSKKVSTAFGLGVAVTVVLGISVPVNNLIY

NLVLRDGALVEGVDLSFLNFITFIGVIAALVQILEMILDKYFPSLYNALGIFLPLIAVNC

AIFGGVSFMVQRDYNFPESIVYGFGSGIGWMLAIVAMAGIREKMKYANVPAGLRGLGITF

ITTGLMALGFMSFSGVQL

>WP_116624776.1

MFQTLFSSSDVTSTALSVATVATLATAVLTMLGLSWTSERWRVPVALSAVALLASGLVYQ

SALNLWLTGHQLTPATRYVAWFVVQPLQICSVFFFARISGAVPSGVFWRTGAAAILMVLS

RYLGDAQIFNPTLGVLLSIAFWLYILGEMYFGAMAEVVRKSSRPIRLGYFWVRLIMTIGW

AIYPILHFVDVVIGAGHVPSVIVLYTVADLVNLIAVSLIVLAVAGEERF

>WP_140455306.1

MRTSPTRLLGTGLLTALLASLCCIAPLLALVGGVTGAISAFGWVEPFRPYLAGVTVAVLA

LAWYQRLKAGKSAAACACEGEASPTFWKSNKFLLAVSCVALLLLAFPEYAGAFYRQQPVA

KAASVQTDFTQSVKLQVKGMTCTGCEAHVNQEIGKLAGVFSVSTSYEKGNAIIKYDSTKV

KPMQILQAAKKTGYTVAIEDKKP

>WP_168247034.1

MPITKGHGNPTWTREETILALDLLYLHGKPVDRKHQDVSQLSEFLRRVDIHPAQSRTEKF

RNPDGVALKLQNLFSAVEPGRGLTYSKTDLEIVTAFPNSRKSELAEIARLLRSSLLTHEL

VEEHVDEEEVFIEGRWLTSRHRYRDIRLRKRLLQSLPKLCCEICDFSPPSLSRSIQESFF

EAHHTIPISAAEGSVATKVLDMALLCASCHRFIHRLIAEEKRWVTPAEARDYLTGKRNDK

LEDRS

>WP_120447158.1

MGLLGGFFNDLQKVVNDSVGNDYRKIYFSAHPEEQQECACCGATLYRGDSDFTIDHIIPQ

KYNGTNFVTNLQPMCRSCNSRKKDKIDALTLKYSGTMLINEIKNLNRKKEW

>WP_120424551.1

MRKHSKEYSSYMKSDAWSAKREERLQLDGNRCVMCGRPNGLQKDSVTPVLQVHHICYSNL

GNEPMSDLVSICPGCHKKIHKYYRRLRSWEDKEVVARA

>WP_120423357.1

MIKVERKITEKSRRAMDSLERERLKNGSYNTPEVNAALKEMFHGKCYICENKQITSYHIE

HLNPHHGNIELKYSWDNLFLSCAHCNNIKSDKFDPIIDCTKENVEDMIAFRKEGYFGRDE

KLIFDMLDSRIETQNTIKLLQEVYYGSTPQKKMEATILRRTLRKELSDFKEYVREYQESE

DEEKEDLMYLLQMQLSSSSPFAAFKRWLIRDNKDVYPELLEYID

>WP_160581195.1

MKKHQKTLAVLLTAAMLVSLTACSSGGKDSTTAAEAAKTETSAAAGTQAKAETKAEASQE

PVDIAVIVPQKRGDLGFTDSIYKGVEQVMADYADRVNITFTECAGDSSKFESTIYDVCDQ

GPDLIITPSGSGFADLIATKAAHDYSDIKFVLVDNSAAYAGITTDNVAGMSYKQNEATFL

TGALAALLNETGMIGYVAGMSNAVINDFTVGYIQGAQYINPDIKIQISYIGDFADSAKAK

ELAATQIGLGADVVAQVAGTAGLGVLDAAKEAGVWGIGVDADQAAAYKESNPEMSAIIAS

SAMKNGASLLVSIVDRFIGENDLPWGGIESQGLVEGAVEIAPIADNVPDEVKKQISELQE

KVIKGEIEVKSAFTISEEEFNEYVNSCQ

>WP_135856548.1

MATSYDYAPLFRSTVGFDRIFNLLENAQRARSISDWPPYDIIKTGDDSYRISVAVAGFAE

DELDITFQSNLLTVTGKKQDASADEYLHRGIAGRPFEHRFELADHVRVNGADLRNGLLSI

DLVREIPEALKPRKIDIQTSPALQHKVAPAQIEAQKAA

>WP_135901797.1

MSESRIRFRHLQAFLEVARQRSVAKAADFLHVSPPAVTKTLRELEEALGVAVVERDGRGI

RVTRIGEIFLRHAGTAITALRQGVDSVRQDGAINRYPIRIGALPTVSAKVMPHAMSLFLK

ENTSAAIKIVTGENAVLLEQLRTGALDLVVGRLAAPENMTGFFFEHLYSEQVLFVVRSGH

PLLELGADIFARLDAFPVLMPTRESVIRPFVDRLFITNGMTAPATEIETVSDSFGRAFLR

QSDAVWIISAGVVANELGSGAFVALPVDTEETKGPVGLTMRTDTAPSPAFSILLQTIREA

ARPRA

>WP_120435514.1

MLFIWESWHFWLFFVLGACFYYRRNEAEYE

>WP_120446042.1

MKKLLEEIEKNQELKAKIEELDKNPKSTPKDYIQAAAEYGIEIKEEDFKTTRGELSDDEL

DAVAGGKVCSCFVGGGGEGGRRDKICACVAFGAGEDNYADTLRCFCPLAGSGDTHDH

>WP_007225590.1

MDKLIRDSKAAFIIAMALTIWVCAPGAALAGPAYDTYGTVTFDGIKTDVNEYTGGVSSGS

LDLQWFNDHESKNFRYADNVTNALLWEINESSDSPTVWSLNVFFEVPTDARRMIWEDGCT

WIKGGIEGTSCDGLKGLPNGEAILDAYADGSHHFSSKKESKKESKKESKKGSKKESKKES

KKESKKESKKESKKESKKESKKESKKESKKDHEKHSQEGKKEAKMSYSTQTGSEEFSIGE

GEAANNWFGLQKWQDEDENVKDDGSWLTSREYLIENELCDTTFCDAWDSSFSVELLFLFN

TQAGAQNKIKSLTDESVNYAMRLHLSDEANGIDSVTVPEPGPGILLILGLAGLGFARRKA

Q

>WP_140972653.1

MNAYIREPVNAFTHLGGAVLSFIALLAMIVKVSVKMPSFASITAVILFGIGMMVLYTASA

VYHSVVASERVIYFFRKLDHSMIFILIAGTYAPFCLITLHSASGLLLFCLVYATAICGIV

FKMFWFSCPRWLSTAIYITMGWLIVLFFAPLAANLSTGGMVLLVLGGILYTIGGFIYGTK

PKWLEFKYMGHHEIFHVFVLLGSLAHFLSVYCYVI

>WP_169252559.1

MPDSVPTGLIAILRGVRSDEVLDIAEGIVDAGFSAIEVPLNSPDPLASISALVEKFGDSV

EIGAGTVLTADQVRECRQAGARIIVAPDTDRDVITTALELGLTPYPGAATPTEAFAAVKA

GATNVKLFPSSAVGISGMKAWREVLPSGTELFPVGGVGADNAAEWRKAGAAGLGLGSSLY

RRGDRPDDVRTQAQAIASAWAQSI

>WP_169251584.1

MLAVLIGACVGLGVLLWSGSTRRRLQSLLGEGRTDPTGTEAATPEAGPAESGTEPAVADD

QLAFDLDLVAICLTSGLPIPVALTLTAEATDDRSDLQRIARAMTIGGRRLADDDRLLPVL

EVFEFSEHTGVGPAPLIESVAEELRASSRRRRQEAAASLGVQLVLPLGVCILPAFLLLSV

VPVVISLLTDLTTVFF

>WP_169253902.1

MNTDEDVRAAIASLLRIGEPGDGLLKRLVDGIGPVAAQGIIMAVGRGETTAHEAVHGLTV

TGTEAAEHGQMPEAIDRWAVRAGDVETRGDDLDKIARIGGRLVIPDDDEWPRMLDDLGPA

APLGLWVRGAASLSTVLARAVAVVGARAASSYGTKCASDLAWDLAARGITVVSGGAFGID

AAAHRAAIAREAPTVAFMAGGVDRFYPAANADLFEQILSTGAIVSETAPGMTPMRHRFLL

RNRLIAASAQVSVIVEAGWRSGALNTARHALELSRQVAAFPGSVYSASSTGAHKLVREHE

AELVTCSDDVIALMDDETPALFDATAAGAAAENGERPPPPDPREALDEREKICLNALTVS

KPLDVGTIASRAGLTIADALNSLTTLDLAGMAERRDTGWVKLRTSRG

>WP_169253261.1

MNIRRMTVAAVAAVALALTGCGSDGGSGGGGETTDDLTLGTGGTSGTYYPLGGELASIFE

DNVDGVTVNYVESGASAENLGKIYQGEWQLGFTQSDTANTAVNGELEDLDGTKIDNVGWL

ASLYPEAAHIIVREDSGIESVEDLKGKKIAVGDAGSGTRAISDAILDAAGIGESDYTPEI

TDFGASTDMLADKQIDATIFVVGTPVAGLTQLAATTDVKLLGLDDDTTKTIEEGSGAESY

DIPADAYDFLDEDVPTVSVFASLVASTDQVSEDTAYNLTKALFEHTDDITLDVGKLITKD

SAMLGVGDVPLHPGAQKYFEEEGIELP

>WP_040823762.1

MKIFQRVSLRLSLALMCTALFAGTVAAESTASMPLVSPGPHEVVEKTTQQVMEVITSAKG

YYATDPQRFYSEIESVLEDVIDFDGFSRGVMGQYASKKMYVSLETDEEKSAFKERMRRFS

ATFRNGLVQTYAKGLLAFNGNRIDVLPPIESKDLSGTDSVTVTQHIFGEAEKPFVIQYKL

RPNRAGEWKLRNVTIEAINLGIVYRGQFNSAVRLYDGDIDKVIDNWSVDPTGSAKSS

>WP_007235350.1

MQRIDVYWRDIPAQVLIKRGRDRGKHLLSHRFQAAIDRAAMKAGKGGSDAYLEEWRRVTT

SIEAEGSVKDIAQEFGEQIEAQYSDDDIARLVAQKGFDEALT

>WP_007229782.1

MTTISLFPLSGVLLPHGKVPLQIFEQRYIDLVRSSMKTGDPFGIVWIRRGSEVAGRGRAS

SELGDWGTLARIVDWDQLPNGLLGITIQGEGRFDLYETETQSNGLVLGEVVYRDNPASVS

MEAKWQPMLDVLQSLESHPHVQQMGLQLDYGDAWNVAWALIQLLPLEEYLKYELLGLDAI

DEVMSELDLILNQISGED

>WP_007227784.1

MEKVIVCWRDVPAQVIIKHRRKRATVELSERFQKAIDKAAMRAGKADSDAYMEDWCRRSS

PHAGVGSLEDIAKNVADSIESDYSDDVLSKMVADVGYRVR

>WP_007228934.1

MAMDRPQVLIVDDDPRVCRLIQNMACSEQFEYTDIVDPRRLKTVYEDLLPEKIFLDLSMP

GMDGIEALTFLKDSGSTSHICLISGWSEKVLRSSCALGKKMGLNMSPPIHKPFRATEIRA

FLKADQFTHTSVPVSPILPRSRKFKEELKHAIYNVGEIQPFYQPIVDLKTGEVDSLEALC

RWHHPTRGILCPGDFLPLVEKYGLMKDLTYSLLKIILEDMACWDSMASRPNVSINLFPEL

LEEQSLPDVFMAHMKSAGIDPSRITIEITEQSNYGDSVQMMNVISRLRIAGFGLSLDDFG

TGFASMEKVKEIPFTELKIDRSFVSDLLHDPDAKAIVKSSISLAQEIGIPTVAEGIENRE

TLSWLISNGCTRGQGYLLCPPGDFDKSILLAQSSTNHKYEIGEFDSGDVSSSTDTVSTKE

CQTTATLTVTPDIT

>WP_007227234.1

MTGTILLVEDNELNRDMLIRRLVRAGKEVVSAADGQQALDLMRSEKPAVVLMDMNLPILN

GWTACRQARADDTIKHIPIIALTAHASDADRLNALEAGCDDYATKPVDFPGLLIKIEKLT

GNC

>WP_007225255.1

MTLILLVEDNDMNRDMLSRRLQRRQYRVTTANNGAIAVEKATLEKPDLILMDMELPIKDG

WTASREIKATLDTPIIALTAHALSGDRDKALAAGCDDYTTKPINFDHLVAMIEQYLGKKN

N

>WP_007226686.1

MQPPSNHPPLILASSSAYRRQLLLKLNLSFDCVNPCIDETAGSNETADQLVARLAREKAL

AVIHSHPAHLIIASDQVAVLDGVIMTKPGDHGSAIAQLRQCSDKKVIFYTGLTLLNSSTG

RLQNAVEPFSVYFRKLDATTIERYLANEKPYDCAGSFKVEGLGITLFKKLEGDDPNSLIG

LPLIQLTSMLANEGILRP

>WP_007233776.1

MTTHKVLIVDDELPIRDMLRMALETAGYECLEAETIDAAYHQIVDDRPDIVLLDWMLPGG

SGIELLRRIKRAEMTQDLPVIMLTAKAAEHNVIQGLDVGADDYITKPFALRELLARIKAL

LRRAKSSDDRNLLVVRDLTIDIDSRRAFVGEEALQLGPTEFNLLLFFMSHPERAYTRSQL

LDRVWGANIYVEERTVDVHIRRLRAALDAAEGDYSQLIQTVRGTGYRFSEQGG

>WP_007225159.1

MEEKTILIVDDEAPIREMIRMSLDMAGFNCREAADTREAYRVIADSKPDLVLLDWMLPGG

SGIELLRRLRKEELTADLPVIMLTAKTDEDNKIQGLDVGADDYITKPFAPREMLSRIKAL

LRRTSTGIGGSVIEVQGLKLDISSHRVYIDTRPVDMGPTEFRLLSFFMTHQERAYSRGQL

LDHVWGGNVYVEERTVDVHIRRLRKALESEGGCYNECVQTVRGTGYRFSSKSIPPA

>WP_007226710.1

MTQDTNPPGVPEGFRTLRNSAHAETHVGPFYYKKDDDELTLGFLAGDQHSNAIGGVHGGV

LMFFADYAVVMSAMKGQKENCATISASCDFVSSAHTGEWVEAEATITRRTGSMVFVSGRI

YVGDKTVMTVQSVLKRIIPREK

>WP_040541238.1

MSQRIVIDPNDPVTCPDCSHEFPLVQGISHHLIERYEEEYDQKLAEEREALEARAVRTAE

RQLSGRFEEQLGDLTDKLEDAQAEREKAHKKLTKEKARAADQAREEAAEELSDLKQQLGE

KDEKLEDFRKEELALRKAKQVLDQEKRDLELTLQRQLEEQQSALRAELGNEFQLREAELR

KKIDDAHSANEDLKRKLEQGSQQLQGEVLELELEEILSQAFPIDQVDAVSKGVRGADVIQ

TVNLRSGASAGKIVWETKRAENWSNKWVSKLKDDQQSVGGEIGVLVSTAYPANVDEPFTQ

IDGIWLVRPEFAKPLADALRAILIEAFRQRTASSGKNEKMEALYDYVCSAQFAQKVRAVL

DAYAAMRDDLEREKAAMQRLWKKREGQLERITVNVVGICGELQGLSTASLPHLDEIAPIE

VA

>WP_009773675.1

MGKNITVVAAIAVATLLMGACSSGEVIDPAEGNPGADLRAGEAYDPRAFEGESINMLLIE

HPFVNSLRPLIPDFEAATGITVNLEVLNEQQGFDKLQADLSAGVGNYDLFMTDPLHNWQY

SAAGWIEPLDGYVENDAITMPDYNIDDFAPGVLDAGRWNRELLTGLGEGSLWALPVNFES

YNLTYRPSMFEDAGVEVPTTYEDVLDVTESLATSLSGNNYPIVTRFDKYWDLTYLTFGSM

AESYGVNLLNDDGEVDIASDASVEVTDLFIDIIKAGSPQDASAFTWYEVLQGMASGRFAL

ALNEADLFAATYENDAESEIADDVGYALIPEGPEKRAASAWIWQLSMAQASADKGAAWTF

LQWLTSADVLMQTHLAGNMNPVRLSAWEDPELAALVDTWGSEPGQYREVLEGTAEIAAIN

YPPHPELTRALDRWAEAVQQSFFDGNTKANLESAASDIERILLP

>WP_009773511.1

MSPTIVRGMTWEHERGYGSVVKAAEAYRSVAPDVEVQWEYRSLQAFADQDLESLVEQYDL

LVIDHPHIPIAAEEKLFTPLNGRGFDTELATLATQSVGRSHESYKHLGQQWGLALDAAAQ

VAAYRPDLLESPPRNWDEVMALAEEGRVLWPFKPVDAYSSLITIAAGLGEDPMATAGVFL

SEEMLTRAMELLVRLARLVPADNAGFNPIQVADVLAESDIFAYSPLLFGYTNYSRVGYRS

KRVQYTDIPSSTRGVAGSLLGGAGIAVSSRSRVMDAAIAHAFWLASGPVQEGSYYDGGGQ

PGNAVAWESARTNSDSLDFFTGTRATLEGAYMRPRFATYIELQNAVSPFVTSALLGEITI

TELRERLDAGVAEWLVR

>WP_007235958.1

MTIDIDHRRVLIVDDQSTRAHHLIDALGMDAFEFDVANEVPDLQGALGPDSPWDCVLCNA

GLINVSWASVRRAMRNFDVQVPVIVVADEQNVDSMKTALGLGATDFFVKPHARPGLLKRS

IERCVNHRYLQRELKASKEDVERSNTELRHSLRVLEQDQQAGRQVQRALLPSGALHQGDY

WFSHTIVPSLYLSGDFTDYFSVGEDQIAFFLADVSGHGSSSAFATVLLKNLFARKRSDFL

RRGDHSVVSPKDMLELANNELLELAINKYATMIVGVLNFKSHQLTYSIAGHLPHPVLLDE

NSVRYLEGEGPPVGLMRDARYTQHEVVLPEHFVLALLSDGILELLGNGNLIEKEASLLSL

LEGPLESPRSLATRLGLEAVDPNHLPDDVAALFITRGFS

>WP_007227112.1

MGNCETKVLILEDDPLAASELKDCLAREGLHPSIARSKEHFEKIVEQHEFQLLIVDIGLP

DGSGLDVIREVREQSSVGIIVVSGYTTESDVVAAIELGADDYIKKPISIKELRAKVRRMM

IRTSGNGYSRSIAQPNNNEQKFFGDWHLDLDSHRLFYKINHEVGLTSAEYKILLALLNNC

DQVLSRHSLLNHLQSISSPYDERTIDGLINRVRKKLAIPASYEPVQKVRNAGYMFCETVR

TEHQQTTESPGSGFSLTAEKMTIDKAPSPTLEPPVFSSAPESGSTKLTH

>WP_007224478.1

MSSVEQLIQDSRLWRGKHYRDDHSQQTGNSISSGITQLDQQLHWRGWPLHSSSELLCEHW

GIGELSLLMPLLKKVSHKGRIAWINPPFIPYSPALLSQGITPEKCLLLYPSESDQWWAAE

QVLASSAFAIVMTWFTRQASNATPYRRLQAAAEKGHCLHFHFRPLSSKQQSSPARLRIQL

SSSASQLAVEVLKQPGGWSGQQLVISRPESLLFKQQAVEKWPVYHSSRPSYQVVNGRTDI

PSIIDPQHSDRLNDDQSIIHQPSSSAPTQPH

>WP_007223999.1

MAILALIQNNIDRFSDVTGRILAWLCLLLMLLSCSVVFIRYGLGAGSIALQESVTYLHGT

IFMLGAAYTLRHDGHVRVDIFYRNMSARSKAWVNCGGGIIFLLPLCVYFFISSWGFVQQS

WEFREISSEPGGIPAVFLLKTLIPLMAVNLGLQAFAETLRNLLILIAREDSVQL

>WP_007223681.1

MSDLSPQEIEILHEALDDEYLAWSTYDQVIEDFGEISPFINIREAESRHIEALCTLFNRY

GVPVPPNPWLGRVERYKSIQEACEAGVKAEIANGEMYERLMVATQRRDFLEVLGNLQEAS

QKRHLRAFERCVSRRGSGCGAGRGRGRGNGGRC

>WP_007234293.1

MDLATLLGLLGGLAVVGTAIFYGGAGPTFYNVPSILIVIGGTFMTVMVKFSLKQFLGAFK

VAGRAFSNKSHDPESLIAEIVNLANIGRKEGLLALEKAAISESFLKDGIQMLVDGSNQEV

VKAVMAKDMQQTMDRHNWGERVWRAVGDVAPAMGMIGTLVGLVGMLVNMNDPKAIGPQMA

VALLTTLYGAVLANMVALPIADKLHLRKSNEKLIHQMCIDGVLAIQAGQNPRVIESMLKA

YLDPAHRDKNANSGK

>WP_007236262.1

MSAVLVKELRERTGLGLLECKRALKEADNDIDAAIEALRKSSGMKAAKKAGRIAADGVVT

TRTAEDGSYGVLVEVNSETDFVARDENFLGFVGSVADTLYESRSADIDALKSGSLEQARE

ALVQKIGENIGIRRASLVTAENGVVGSYVHGNNRIAVLVELRGGDQDLARDVAMHVAAVN

PQVVSPADMPEALLEKERDIFTAQAQESGKPAEIIEKMIGGRIKKYLAENSLSEQAFVKD

PDVTVGQLVKAADAEVISFSRFEVGEGIEVDKVDFADEVAAQLKG

>WP_007225796.1

MAAVSASMVKELRDRTGLGMMECKKALVEAGGDIDAAIEEMRKNSGMKAAKKAGRTAAEG

VVTAKVAEDGSYGIVVEVNSETDFAARDESLLAFVATVSEKVFTEKQTDVKALMEGDLNT

AREALVQKIGENISVRRSEVVDSDGVVGSYVHSNNRIAVLVSLTGGDAELARDIAMHVAA

VNPQVVRPEDMPEDVVTQEKNIIKAQPDMEGKPEAIVEKMMIGRINKFLKENSLLEQAFV

KDPEITIGKLAKNAGAEVVSFVRYEVGEGIEKEEIDFAAEVAAQLNG

>WP_007233347.1

MSVATRVRTFFQQARDSLVLSGFAKTDGPMGKIVAGVGALYLIVMIVLAIWWSAAPPAFD

MTTKTQDYSTASGQPLVPGSATTLALIEVIDTLLEKDGGYTHNDLLPPGLFIDNMPNWEY

GVLVQSRDLARALREVLSRSQSQSREDVDLTLAEPRINFQSDSWILPASEREYRSANKYL

KAYLARLPEKGPEGARFYARADNLGFWLGMIEKRLGSLSQRLSASVGQRRLNTDLAGDPT

ASAATIDPEEQEIKTPWSEIDDVFYESRGAAWALIHLLKGAEIDFAGVLEKKNARVSLQQ

IIRELEATQGIVWSPIILNGSGFGLWANHSLVMANYISRANAALIDLRELLAQG

>WP_007230569.1

MSVVDRDLRAAGTEVLSLADPLYPPLLKTIPDPPPVLHVRGNPMLLARPQLAIVGARRAS

AAGLQAAHKLAVAAVRAGLGVTSGLALGVDGAAHRGALSAGGDTVAVMATGIETIYPHRH

EPLGQEIASSGCLVTEFPPGTKPLPYHFPKRNRIISGLSLGVLVVESALPSGSLITATSA

MEQGREVFALPWSISHKGGAGCLSLIRDGAKMVLGIEDILEELDSLFGLQQELSQVSTIP

SPESISEQDCLLLELLGFEVISLDQLVVASGLPVGQVMGELSSLELAGRVNRCPGGYIRS

R

>WP_007226893.1

MEHYLSLFVSAIFIKNMALSLFLGMCTLLALSKKMNAAIGLGIAVVVVLSITVPVNYLIY

TYLLREGALVWLSPEFASVDLSFLGLLSYIGVIAALVQILEMFLDKFVPALYNALGVFLP

LITVNCAILGASLLMVEREHDFGESVVFGVGAGVGWAIAIILLAGIREKMKYSDVPAGLQ

GLGITFITVGLMSLGFMSFGGIDI

>WP_007225006.1

MYKILTLNQISTKGLDKFPREDYEIASEFVTSDAVLVRSHKLQPADIQDSVLAIGRAGAG

VNNIPVDYCTEQGIPVFNTPGANANAVKELIVSALTLGSRGILEGIDYVNTLDDLTDGAA

MSKLLEKEKKRFKGNELSGKTLGVIGLGAIGSMVADTALALGMKVAGYDPALSVDAAWRL

SSEVEKVDNITSLVSRADFITLHLPVLDATRKMINRELLSHLKSGAVLLNFAREEIVDTT

AVVEVLDSGKLSKYIADFPTPELIGKRGAVLTPHIGASTDEAEENCAIMAAVQLKDFLEN

GNIKNSVNFPPLYLERTPQSGSVRLSISNRNVPKILGSILSILADENINVIDMLNKSRED

IAYNLIDLQSSPPEQVLEIMRKIDGVVNVRLIG

>WP_007235663.1

MTALSINLNKIALVRNSRVTTVPNIVSHAEMCISAGADGITVHPRPDQRHIRAQDCFDLQ

SALDVELNIEGNPFTEPRASDQPHVGDYPGFIALVQAISPAQVTLVPDSDQQLTSDHGFD

VARDGKRLEPLIKIFKDLGCRVSLFMDPDPSAMATVASLGADRIELYTESYARAHEVGDF

EVSLAAFQETAEAAFAARLGVNAGHDLNLSNLPDFKVPHLEEVSIGHSFTVDALRWGIAN

TIPRYQQALGKNC

# Supplementary Tables

## 2.1 Pearson correlation coefficient-reduced features

**Table S1.** List of features obtained from correlation analysis

| aac_1 | dipep_67 | dipep_153 | dipep_239 | dipep_325 | pseudo_11 | dist_17 |
| --- | --- | --- | --- | --- | --- | --- |
| aac_2 | dipep_68 | dipep_154 | dipep_240 | dipep_326 | pseudo_12 | dist_18 |
| aac_3 | dipep_69 | dipep_155 | dipep_241 | dipep_327 | pseudo_13 | dist_19 |
| aac_4 | dipep_70 | dipep_156 | dipep_242 | dipep_328 | pseudo_14 | dist_20 |
| aac_5 | dipep_71 | dipep_157 | dipep_243 | dipep_329 | pseudo_15 | dist_21 |
| aac_6 | dipep_72 | dipep_158 | dipep_244 | dipep_330 | pseudo_16 | dist_22 |
| aac_7 | dipep_73 | dipep_159 | dipep_245 | dipep_331 | pseudo_17 | dist_23 |
| aac_8 | dipep_74 | dipep_160 | dipep_246 | dipep_332 | pseudo_18 | dist_24 |
| aac_9 | dipep_75 | dipep_161 | dipep_247 | dipep_333 | pseudo_19 | dist_25 |
| aac_10 | dipep_76 | dipep_162 | dipep_248 | dipep_334 | pseudo_20 | dist_26 |
| aac_11 | dipep_77 | dipep_163 | dipep_249 | dipep_335 | pseudo_21 | dist_27 |
| aac_12 | dipep_78 | dipep_164 | dipep_250 | dipep_336 | pseudo_22 | dist_28 |
| aac_13 | dipep_79 | dipep_165 | dipep_251 | dipep_337 | pseudo_23 | dist_29 |
| aac_14 | dipep_80 | dipep_166 | dipep_252 | dipep_338 | pseudo_24 | dist_30 |
| aac_15 | dipep_81 | dipep_167 | dipep_253 | dipep_339 | pseudo_25 | dist_34 |
| aac_16 | dipep_82 | dipep_168 | dipep_254 | dipep_340 | pseudo_26 | dist_35 |
| aac_17 | dipep_83 | dipep_169 | dipep_255 | dipep_341 | pseudo_27 | dist_37 |
| aac_18 | dipep_84 | dipep_170 | dipep_256 | dipep_342 | pseudo_28 | dist_38 |
| aac_19 | dipep_85 | dipep_171 | dipep_257 | dipep_343 | pseudo_29 | dist_41 |
| aac_20 | dipep_86 | dipep_172 | dipep_258 | dipep_344 | pseudo_30 | dist_44 |
| dipep_1 | dipep_87 | dipep_173 | dipep_259 | dipep_345 | amphipseudo_21 | dist_47 |
| dipep_2 | dipep_88 | dipep_174 | dipep_260 | dipep_346 | amphipseudo_22 | dist_49 |
| dipep_3 | dipep_89 | dipep_175 | dipep_261 | dipep_347 | amphipseudo_23 | dist_50 |
| dipep_4 | dipep_90 | dipep_176 | dipep_262 | dipep_348 | amphipseudo_24 | dist_52 |
| dipep_5 | dipep_91 | dipep_177 | dipep_263 | dipep_349 | amphipseudo_25 | dist_53 |
| dipep_6 | dipep_92 | dipep_178 | dipep_264 | dipep_350 | amphipseudo_26 | dist_55 |
| dipep_7 | dipep_93 | dipep_179 | dipep_265 | dipep_351 | amphipseudo_27 | dist_56 |
| dipep_8 | dipep_94 | dipep_180 | dipep_266 | dipep_352 | amphipseudo_28 | dist_58 |
| dipep_9 | dipep_95 | dipep_181 | dipep_267 | dipep_353 | amphipseudo_29 | dist_59 |
| dipep_10 | dipep_96 | dipep_182 | dipep_268 | dipep_354 | amphipseudo_30 | dist_61 |
| dipep_11 | dipep_97 | dipep_183 | dipep_269 | dipep_355 | amphipseudo_31 | dist_62 |
| dipep_12 | dipep_98 | dipep_184 | dipep_270 | dipep_356 | amphipseudo_32 | dist_63 |
| dipep_13 | dipep_99 | dipep_185 | dipep_271 | dipep_357 | amphipseudo_33 | dist_64 |
| dipep_14 | dipep_100 | dipep_186 | dipep_272 | dipep_358 | amphipseudo_34 | dist_65 |
| dipep_15 | dipep_101 | dipep_187 | dipep_273 | dipep_359 | amphipseudo_35 | dist_66 |
| dipep_16 | dipep_102 | dipep_188 | dipep_274 | dipep_360 | amphipseudo_36 | dist_67 |
| dipep_17 | dipep_103 | dipep_189 | dipep_275 | dipep_361 | amphipseudo_37 | dist_68 |
| dipep_18 | dipep_104 | dipep_190 | dipep_276 | dipep_362 | amphipseudo_38 | dist_69 |
| dipep_19 | dipep_105 | dipep_191 | dipep_277 | dipep_363 | amphipseudo_39 | dist_70 |
| dipep_20 | dipep_106 | dipep_192 | dipep_278 | dipep_364 | amphipseudo_40 | dist_71 |
| dipep_21 | dipep_107 | dipep_193 | dipep_279 | dipep_365 | comp_1 | dist_72 |
| dipep_22 | dipep_108 | dipep_194 | dipep_280 | dipep_366 | comp_2 | dist_73 |
| dipep_23 | dipep_109 | dipep_195 | dipep_281 | dipep_367 | comp_3 | dist_74 |
| dipep_24 | dipep_110 | dipep_196 | dipep_282 | dipep_368 | comp_4 | dist_75 |
| dipep_25 | dipep_111 | dipep_197 | dipep_283 | dipep_369 | comp_5 | dist_76 |
| dipep_26 | dipep_112 | dipep_198 | dipep_284 | dipep_370 | comp_6 | dist_77 |
| dipep_27 | dipep_113 | dipep_199 | dipep_285 | dipep_371 | comp_10 | dist_78 |
| dipep_28 | dipep_114 | dipep_200 | dipep_286 | dipep_372 | comp_11 | dist_79 |
| dipep_29 | dipep_115 | dipep_201 | dipep_287 | dipep_373 | comp_13 | dist_80 |
| dipep_30 | dipep_116 | dipep_202 | dipep_288 | dipep_374 | comp_15 | dist_81 |
| dipep_31 | dipep_117 | dipep_203 | dipep_289 | dipep_375 | comp_16 | dist_82 |
| dipep_32 | dipep_118 | dipep_204 | dipep_290 | dipep_376 | comp_17 | dist_83 |
| dipep_33 | dipep_119 | dipep_205 | dipep_291 | dipep_377 | comp_18 | dist_84 |
| dipep_34 | dipep_120 | dipep_206 | dipep_292 | dipep_378 | comp_19 | dist_85 |
| dipep_35 | dipep_121 | dipep_207 | dipep_293 | dipep_379 | comp_21 | dist_86 |
| dipep_36 | dipep_122 | dipep_208 | dipep_294 | dipep_380 | tran_1 | dist_87 |
| dipep_37 | dipep_123 | dipep_209 | dipep_295 | dipep_381 | tran_2 | dist_88 |
| dipep_38 | dipep_124 | dipep_210 | dipep_296 | dipep_382 | tran_3 | dist_89 |
| dipep_39 | dipep_125 | dipep_211 | dipep_297 | dipep_383 | tran_4 | dist_90 |
| dipep_40 | dipep_126 | dipep_212 | dipep_298 | dipep_384 | tran_5 | dist_91 |
| dipep_41 | dipep_127 | dipep_213 | dipep_299 | dipep_385 | tran_6 | dist_93 |
| dipep_42 | dipep_128 | dipep_214 | dipep_300 | dipep_386 | tran_10 | dist_94 |
| dipep_43 | dipep_129 | dipep_215 | dipep_301 | dipep_387 | tran_11 | dist_96 |
| dipep_44 | dipep_130 | dipep_216 | dipep_302 | dipep_388 | tran_14 | dist_97 |
| dipep_45 | dipep_131 | dipep_217 | dipep_303 | dipep_389 | tran_16 | dist_99 |
| dipep_46 | dipep_132 | dipep_218 | dipep_304 | dipep_390 | tran_17 | dist_100 |
| dipep_47 | dipep_133 | dipep_219 | dipep_305 | dipep_391 | tran_18 | dist_102 |
| dipep_48 | dipep_134 | dipep_220 | dipep_306 | dipep_392 | tran_19 | dist_103 |
| dipep_49 | dipep_135 | dipep_221 | dipep_307 | dipep_393 | tran_20 | dist_105 |
| dipep_50 | dipep_136 | dipep_222 | dipep_308 | dipep_394 | tran_21 | ss_1 |
| dipep_51 | dipep_137 | dipep_223 | dipep_309 | dipep_395 | dist_1 | ss_2 |
| dipep_52 | dipep_138 | dipep_224 | dipep_310 | dipep_396 | dist_2 | ss_3 |
| dipep_53 | dipep_139 | dipep_225 | dipep_311 | dipep_397 | dist_3 | ss_4 |
| dipep_54 | dipep_140 | dipep_226 | dipep_312 | dipep_398 | dist_4 | ss_5 |
| dipep_55 | dipep_141 | dipep_227 | dipep_313 | dipep_399 | dist_5 | ss_6 |
| dipep_56 | dipep_142 | dipep_228 | dipep_314 | dipep_400 | dist_6 | qso_1 |
| dipep_57 | dipep_143 | dipep_229 | dipep_315 | pseudo_1 | dist_7 | qso_8 |
| dipep_58 | dipep_144 | dipep_230 | dipep_316 | pseudo_2 | dist_8 | qso_15 |
| dipep_59 | dipep_145 | dipep_231 | dipep_317 | pseudo_3 | dist_9 | qso_16 |
| dipep_60 | dipep_146 | dipep_232 | dipep_318 | pseudo_4 | dist_10 | qso_17 |
| dipep_61 | dipep_147 | dipep_233 | dipep_319 | pseudo_5 | dist_11 | qso_20 |
| dipep_62 | dipep_148 | dipep_234 | dipep_320 | pseudo_6 | dist_12 | pssm_2 |
| dipep_63 | dipep_149 | dipep_235 | dipep_321 | pseudo_7 | dist_13 | pssm_18 |
| dipep_64 | dipep_150 | dipep_236 | dipep_322 | pseudo_8 | dist_14 | pssm_85 |
| dipep_65 | dipep_151 | dipep_237 | dipep_323 | pseudo_9 | dist_15 | pssm_274 |
| dipep_66 | dipep_152 | dipep_238 | dipep_324 | pseudo_10 | dist_16 | pssm_295 |

## 2.2 ADTree-reduced features

**Table S2.** List of features obtained from ADTree

| dist_62 | amphipseudo_35 | dist_70 | dipep_190 | dipep_213 | dist_86 | dipep_260 |
| --- | --- | --- | --- | --- | --- | --- |
| aac_11 | dipep_14 | pseudo_28 | dipep_18 | aac_12 | aac_8 |  |
| pseudo_5 | dist_99 | dist_63 | pseudo_26 | dipep_11 | dipep_64 |  |
| aac_2 | dipep_22 | dipep_380 | dipep_138 | qso_17 | dipep_8 |  |
| aac_13 | dipep_336 | pseudo_2 | dipep_39 | ss_6 | dipep_114 |  |
| pseudo_3 | tran_11 | dipep_387 | dipep_70 | aac_3 | dipep_279 |  |
| comp_15 | ss_4 | dipep_316 | dipep_116 | amphipseudo_22 | dist_7 |  |

## 2.3 GA-reduced features

**Table S3.** List of features obtained from GA

| aac_9 | dipep_110 | dipep_194 | dipep_285 | pseudo_4 | dist_9 |
| --- | --- | --- | --- | --- | --- |
| aac_11 | dipep_111 | dipep_197 | dipep_292 | pseudo_5 | dist_15 |
| aac_12 | dipep_112 | dipep_198 | dipep_293 | pseudo_6 | dist_20 |
| aac_13 | dipep_115 | dipep_201 | dipep_294 | pseudo_9 | dist_22 |
| aac_17 | dipep_116 | dipep_202 | dipep_298 | pseudo_11 | dist_25 |
| aac_19 | dipep_118 | dipep_204 | dipep_304 | pseudo_14 | dist_27 |
| dipep_3 | dipep_119 | dipep_206 | dipep_307 | pseudo_16 | dist_34 |
| dipep_9 | dipep_121 | dipep_209 | dipep_312 | pseudo_22 | dist_37 |
| dipep_11 | dipep_123 | dipep_210 | dipep_313 | pseudo_23 | dist_44 |
| dipep_16 | dipep_129 | dipep_211 | dipep_314 | pseudo_25 | dist_50 |
| dipep_17 | dipep_131 | dipep_218 | dipep_315 | pseudo_28 | dist_52 |
| dipep_18 | dipep_135 | dipep_219 | dipep_317 | pseudo_29 | dist_53 |
| dipep_21 | dipep_140 | dipep_223 | dipep_319 | pseudo_30 | dist_55 |
| dipep_23 | dipep_143 | dipep_229 | dipep_324 | amphipseudo_21 | dist_58 |
| dipep_27 | dipep_144 | dipep_237 | dipep_326 | amphipseudo_22 | dist_61 |
| dipep_31 | dipep_148 | dipep_239 | dipep_332 | amphipseudo_26 | dist_66 |
| dipep_32 | dipep_150 | dipep_240 | dipep_334 | amphipseudo_29 | dist_68 |
| dipep_36 | dipep_151 | dipep_244 | dipep_335 | amphipseudo_30 | dist_70 |
| dipep_37 | dipep_152 | dipep_248 | dipep_338 | amphipseudo_32 | dist_72 |
| dipep_38 | dipep_154 | dipep_250 | dipep_339 | amphipseudo_34 | dist_73 |
| dipep_39 | dipep_156 | dipep_251 | dipep_341 | amphipseudo_35 | dist_75 |
| dipep_41 | dipep_157 | dipep_253 | dipep_343 | amphipseudo_37 | dist_77 |
| dipep_45 | dipep_159 | dipep_254 | dipep_346 | comp_4 | dist_79 |
| dipep_59 | dipep_160 | dipep_255 | dipep_347 | comp_5 | dist_80 |
| dipep_60 | dipep_161 | dipep_258 | dipep_353 | comp_11 | dist_81 |
| dipep_63 | dipep_165 | dipep_260 | dipep_354 | comp_16 | dist_84 |
| dipep_69 | dipep_168 | dipep_262 | dipep_360 | comp_19 | dist_85 |
| dipep_70 | dipep_170 | dipep_263 | dipep_361 | tran_3 | dist_91 |
| dipep_71 | dipep_173 | dipep_266 | dipep_365 | tran_5 | dist_94 |
| dipep_72 | dipep_174 | dipep_267 | dipep_366 | tran_6 | dist_96 |
| dipep_75 | dipep_175 | dipep_268 | dipep_371 | tran_11 | dist_102 |
| dipep_85 | dipep_176 | dipep_270 | dipep_372 | tran_19 | ss_6 |
| dipep_90 | dipep_177 | dipep_272 | dipep_379 | tran_21 | qso_1 |
| dipep_93 | dipep_179 | dipep_273 | dipep_382 | dist_2 | qso_8 |
| dipep_100 | dipep_180 | dipep_276 | dipep_384 | dist_3 | qso_15 |
| dipep_101 | dipep_182 | dipep_277 | dipep_386 | dist_4 | qso_16 |
| dipep_102 | dipep_186 | dipep_279 | dipep_389 | dist_5 | qso_17 |
| dipep_104 | dipep_187 | dipep_281 | dipep_396 | dist_7 | pssm_18 |
| dipep_107 | dipep_193 | dipep_284 | pseudo_2 | dist_8 | pssm_274 |

## 2.4 Linear SVC-reduced features

**Table S4.** List of features obtained from linear SVC

| pseudo_3 | comp_17 | dist_21 | dist_61 | dist_73 | dist_96 |
| --- | --- | --- | --- | --- | --- |
| pseudo_5 | comp_18 | dist_22 | dist_63 | dist_75 | dist_102 |
| pseudo_8 | dist_2 | dist_24 | dist_66 | dist_82 | pssm_2 |
| pseudo_12 | dist_12 | dist_26 | dist_67 | dist_83 | pssm_274 |
| pseudo_17 | dist_15 | dist_27 | dist_69 | dist_84 |  |
| comp_2 | dist_17 | dist_38 | dist_70 | dist_89 |  |
| comp_15 | dist_18 | dist_59 | dist_72 | dist_91 |  |

## 2.5 Tuning machine learning models

**Table S5.** List of parameters for tuning models and their best values

| **Model** | **Tuning parameters** | **Best parameters** | |
| --- | --- | --- | --- |
| RF | *ntree* = {400, 500}, *mtree* = {5, 6} | ADTree | *ntree* =500 and *mtree* = 5 |
|  |  | GA | *ntree* = 500 and *mtree* = 6 |
|  |  | Linear SVC | *ntree* = 500 and *mtree* = 5 |
| SVM | *C* = {4, 8, 16, 32, 64, 128} | ADTree | 4 |
|  |  | GA | 4 |
|  |  | Linear SVC | 16 |
| DT | *cp* = seq(0.01, 0.1, by = 0.01) | ADTree | 0.01 |
|  |  | GA | 0.01 |
|  |  | Linear SVC | 0.01 |
| LR | *alpha* = 0:1, *lambda*=10^seq(3, -2, by = -0.1) | ADTree | *alpha* = 1 and  l*ambda* = 0.01 |
|  |  | GA | *alpha* = 1 and  *lambda* = 0.1 |
|  |  | Linear SVC | *alpha* = 0 and *lambda* = 0.0316 |
| KNN | *k* = c(3, 5, 7, 9, 11) | ADTree | 3 |
|  |  | GA | 3 |
|  |  | Linear SVC | 3 |

**Legend:**

*ntree*: Number of trees in the random forest (RF) model

*mtree*: Number of features considered at each split in the random forest (RF) model

*C*: Regularization parameter in the support vector machine (SVM)

*cp*: Complexity parameter in the decision tree (DT) model

*alpha*: Regularization parameter for L1 regularization in logistic regression (LR)

*lambda*: Regularization parameter for L2 regularization in logistic regression (LR)

*k*: Number of neighbors in the *k*-nearest neighbors (KNN) classification

## Probabilistic outcomes

**Table S6.** Probability values for the testing dataset. In the table, the labels ADTree_neg_, ADTree_pos_, GA_neg_, GA_pos_, SVC_neg_, and SVC_pos_ indicate probability scores for ADTree-based features (with the negative/non-bacteriocin sequences class), ADTree-based features (with the positive/bacteriocin sequences class), GA-based features (with the negative/non-bacteriocin sequences class), GA-based features (with the positive/bacteriocin sequences class), linear SVC-based features (with the negative/non-bacteriocin sequences class), and linear SVC-based features (with the positive/bacteriocin sequences class), respectively.

| **Sequences** | **ADTree_neg_** | **ADTree_pos_** | **GA_neg_** | **GA_pos_** | **SVC_neg_** | **SVC_pos_** |
| --- | --- | --- | --- | --- | --- | --- |
| BAC005 | 0.013475 | 0.986525 | 0.013475 | 0.986525 | 0.048532 | 0.951468 |
| BAC034 | 0.010275 | 0.989725 | 0.010275 | 0.989725 | 0.022715 | 0.977285 |
| BAC042 | 0.005668 | 0.994332 | 0.005668 | 0.994332 | 0.050973 | 0.949027 |
| BAC045 | 0.005204 | 0.994796 | 0.005204 | 0.994796 | 0.106593 | 0.893407 |
| BAC052 | 0.001299 | 0.998701 | 0.001299 | 0.998701 | 0.043899 | 0.956101 |
| BAC074 | 0.010017 | 0.989983 | 0.010017 | 0.989983 | 0.018609 | 0.981391 |
| BAC097 | 0.16745 | 0.83255 | 0.16745 | 0.83255 | 0.004008 | 0.995992 |
| BAC100 | 0.005121 | 0.994879 | 0.005121 | 0.994879 | 0.002094 | 0.997906 |
| BAC116 | 0.001272 | 0.998728 | 0.001272 | 0.998728 | 0.025705 | 0.974295 |
| BAC128 | 0.003695 | 0.996305 | 0.003695 | 0.996305 | 0.015254 | 0.984746 |
| BAC142 | 0.034552 | 0.965448 | 0.034552 | 0.965448 | 0.059181 | 0.940819 |
| BAC150 | 0.000623 | 0.999377 | 0.000623 | 0.999377 | 0.063341 | 0.936659 |
| BAC154 | 0.017515 | 0.982485 | 0.017515 | 0.982485 | 0.038159 | 0.961841 |
| BAC170 | 0.015782 | 0.984218 | 0.015782 | 0.984218 | 0.040605 | 0.959395 |
| BAC178 | 0.020152 | 0.979848 | 0.020152 | 0.979848 | 0.072472 | 0.927528 |
| BAC182 | 0.007429 | 0.992571 | 0.007429 | 0.992571 | 0.032411 | 0.967589 |
| BAC187 | 0.024394 | 0.975606 | 0.024394 | 0.975606 | 0.244967 | 0.755033 |
| BAC196 | 0.010256 | 0.989744 | 0.010256 | 0.989744 | 0.015421 | 0.984579 |
| BAC203 | 0.194019 | 0.805981 | 0.194019 | 0.805981 | 0.468071 | 0.531929 |
| BAC208 | 0.000916 | 0.999084 | 0.000916 | 0.999084 | 0.014903 | 0.985097 |
| BAC219 | 0.015446 | 0.984554 | 0.015446 | 0.984554 | 0.039269 | 0.960731 |
| BAC223 | 0.00768 | 0.99232 | 0.00768 | 0.99232 | 0.077401 | 0.922599 |
| BAC226 | 0.001348 | 0.998652 | 0.001348 | 0.998652 | 0.00885 | 0.99115 |
| BAC227 | 0.011643 | 0.988357 | 0.011643 | 0.988357 | 0.005826 | 0.994174 |
| WP_061432710.1 | 0.009184 | 0.990816 | 0.009184 | 0.990816 | 0.016635 | 0.983365 |
| CAX48972.1 | 0.002087 | 0.997913 | 0.002087 | 0.997913 | 0.00774 | 0.99226 |
| sp\|Q09T02.1\|MICA_CLAMM | 0.381212 | 0.618788 | 0.381212 | 0.618788 | 0.297348 | 0.702652 |
| CAA74348.1 | 0.042364 | 0.957636 | 0.042364 | 0.957636 | 0.046524 | 0.953476 |
| AAL73241.1 | 0.324889 | 0.675111 | 0.324889 | 0.675111 | 0.019186 | 0.980814 |
| sp\|Q52052\|Q52052_9ZZZZ | 0.031895 | 0.968105 | 0.031895 | 0.968105 | 0.041561 | 0.958439 |
| BAD74571.1 | 0.433002 | 0.566998 | 0.433002 | 0.566998 | 0.13045 | 0.86955 |
| BAD72777.1 | 0.009625 | 0.990375 | 0.009625 | 0.990375 | 0.019414 | 0.980586 |
| ANP43734.1 | 0.011373 | 0.988627 | 0.011373 | 0.988627 | 0.074652 | 0.925348 |
| AAK32702.1 | 0.479604 | 0.520396 | 0.479604 | 0.520396 | 0.008805 | 0.991195 |
| ARW80050.1 | 0.449374 | 0.550626 | 0.449374 | 0.550626 | 0.14764 | 0.85236 |
| BAB04172.1 | 0.019609 | 0.980391 | 0.019609 | 0.980391 | 0.037697 | 0.962303 |
| AAC69560.1 | 0.149492 | 0.850508 | 0.149492 | 0.850508 | 0.004751 | 0.995249 |
| WP_043998581.1 | 0.409639 | 0.590361 | 0.409639 | 0.590361 | 0.121758 | 0.878242 |
| CAP64339.1 | 0.227485 | 0.772515 | 0.227485 | 0.772515 | 0.553686 | 0.446314 |
| sp\|Q07642.1\|LANSB_STRGR | 0.076657 | 0.923343 | 0.076657 | 0.923343 | 0.065342 | 0.934658 |
| NP_604414.1 | 0.01105 | 0.98895 | 0.01105 | 0.98895 | 0.011321 | 0.988679 |
| AAG29818.1 | 0.011107 | 0.988893 | 0.011107 | 0.988893 | 0.004845 | 0.995155 |
| AAZ29031.1 | 0.033578 | 0.966422 | 0.033578 | 0.966422 | 0.011506 | 0.988494 |
| ZP_03980216.1 | 0.104993 | 0.895007 | 0.104993 | 0.895007 | 0.04497 | 0.95503 |
| AAQ95741.1 | 0.003885 | 0.996115 | 0.003885 | 0.996115 | 0.012814 | 0.987186 |
| ACR43769.1 | 0.422222 | 0.577778 | 0.422222 | 0.577778 | 0.505739 | 0.494261 |
| CAA75396.1 | 0.132676 | 0.867324 | 0.132676 | 0.867324 | 0.023792 | 0.976208 |
| NP_345048.1 | 0.021084 | 0.978916 | 0.021084 | 0.978916 | 0.002439 | 0.997561 |
| AAY44084.1 | 0.008087 | 0.991913 | 0.008087 | 0.991913 | 0.002053 | 0.997947 |
| AAP44566.1 | 0.00258 | 0.99742 | 0.00258 | 0.99742 | 0.003425 | 0.996575 |
| AAP44567.2 | 0.000786 | 0.999214 | 0.000786 | 0.999214 | 0.010097 | 0.989903 |
| YP_025360.1 | 0.003674 | 0.996326 | 0.003674 | 0.996326 | 0.034882 | 0.965118 |
| AAG29099.1 | 0.01246 | 0.98754 | 0.01246 | 0.98754 | 0.064004 | 0.935996 |
| AAT90329.1 | 0.057799 | 0.942201 | 0.057799 | 0.942201 | 0.137158 | 0.862842 |
| CAE09438.1 | 0.478889 | 0.521111 | 0.478889 | 0.521111 | 0.312039 | 0.687961 |
| YP_366690.1 | 0.218989 | 0.781011 | 0.218989 | 0.781011 | 0.105099 | 0.894901 |
| WP_142482129.1 | 0.990866 | 0.009134 | 0.990866 | 0.009134 | 0.982198 | 0.017802 |
| WP_160213701.1 | 0.996238 | 0.003762 | 0.996238 | 0.003762 | 0.999511 | 0.000489 |
| WP_142428358.1 | 0.99396 | 0.00604 | 0.99396 | 0.00604 | 0.990517 | 0.009483 |
| WP_142422626.1 | 0.980753 | 0.019247 | 0.980753 | 0.019247 | 0.964389 | 0.035611 |
| WP_081722951.1 | 0.986387 | 0.013613 | 0.986387 | 0.013613 | 0.962697 | 0.037303 |
| WP_001372261.1 | 0.999085 | 0.000915 | 0.999085 | 0.000915 | 0.997717 | 0.002283 |
| WP_160212813.1 | 0.996139 | 0.003861 | 0.996139 | 0.003861 | 0.986928 | 0.013072 |
| WP_159120252.1 | 0.999916 | 8.44E-05 | 0.999916 | 8.44E-05 | 0.998554 | 0.001446 |
| WP_105930623.1 | 0.976537 | 0.023463 | 0.976537 | 0.023463 | 0.942439 | 0.057561 |
| WP_142437480.1 | 0.922548 | 0.077452 | 0.922548 | 0.077452 | 0.924775 | 0.075225 |
| WP_032850333.1 | 0.963593 | 0.036407 | 0.963593 | 0.036407 | 0.993064 | 0.006936 |
| WP_149888968.1 | 0.86 | 0.14 | 0.86 | 0.14 | 0.780532 | 0.219468 |
| WP_116624776.1 | 0.989153 | 0.010847 | 0.989153 | 0.010847 | 0.978367 | 0.021633 |
| WP_007273123.1 | 0.999139 | 0.000861 | 0.999139 | 0.000861 | 0.995475 | 0.004525 |
| WP_121699089.1 | 0.973591 | 0.026409 | 0.973591 | 0.026409 | 0.721221 | 0.278779 |
| WP_120424551.1 | 0.974653 | 0.025347 | 0.974653 | 0.025347 | 0.453537 | 0.546463 |
| WP_160581716.1 | 0.999918 | 8.24E-05 | 0.999918 | 8.24E-05 | 0.999516 | 0.000484 |
| WP_135901797.1 | 0.981909 | 0.018091 | 0.981909 | 0.018091 | 0.992598 | 0.007402 |
| WP_007230524.1 | 0.99768 | 0.00232 | 0.99768 | 0.00232 | 0.993401 | 0.006599 |
| WP_140972653.1 | 0.980961 | 0.019039 | 0.980961 | 0.019039 | 0.997677 | 0.002323 |
| WP_140969579.1 | 0.988458 | 0.011542 | 0.988458 | 0.011542 | 0.938359 | 0.061641 |
| WP_040822867.1 | 0.974677 | 0.025323 | 0.974677 | 0.025323 | 0.867829 | 0.132171 |
| WP_169252584.1 | 0.994541 | 0.005459 | 0.994541 | 0.005459 | 0.971283 | 0.028717 |
| WP_169252559.1 | 0.974633 | 0.025367 | 0.974633 | 0.025367 | 0.899083 | 0.100917 |
| WP_169251583.1 | 0.999582 | 0.000418 | 0.999582 | 0.000418 | 0.959133 | 0.040867 |
| WP_169251032.1 | 0.998708 | 0.001292 | 0.998708 | 0.001292 | 0.996003 | 0.003997 |
| WP_169251018.1 | 0.982357 | 0.017643 | 0.982357 | 0.017643 | 0.964958 | 0.035042 |
| WP_169250805.1 | 0.875096 | 0.124904 | 0.875096 | 0.124904 | 0.811684 | 0.188316 |
| WP_169253902.1 | 0.993537 | 0.006463 | 0.993537 | 0.006463 | 0.992921 | 0.007079 |
| WP_148224521.1 | 0.998307 | 0.001693 | 0.998307 | 0.001693 | 0.990031 | 0.009969 |
| WP_009771703.1 | 0.999297 | 0.000703 | 0.999297 | 0.000703 | 0.990986 | 0.009014 |
| WP_007234989.1 | 0.982521 | 0.017479 | 0.982521 | 0.017479 | 0.994344 | 0.005656 |
| WP_007234584.1 | 0.886608 | 0.113392 | 0.886608 | 0.113392 | 0.952232 | 0.047768 |
| WP_007226034.1 | 0.99991 | 8.97E-05 | 0.99991 | 8.97E-05 | 0.99907 | 0.00093 |
| WP_007227708.1 | 0.999219 | 0.000781 | 0.999219 | 0.000781 | 0.995379 | 0.004621 |
| WP_007225159.1 | 0.99936 | 0.00064 | 0.99936 | 0.00064 | 0.999001 | 0.000999 |
| WP_040821591.1 | 0.976058 | 0.023942 | 0.976058 | 0.023942 | 0.984087 | 0.015913 |
| WP_007229945.1 | 0.998384 | 0.001616 | 0.998384 | 0.001616 | 0.999678 | 0.000322 |
| WP_040811348.1 | 0.995784 | 0.004216 | 0.995784 | 0.004216 | 0.966423 | 0.033577 |
| WP_040541238.1 | 0.996764 | 0.003236 | 0.996764 | 0.003236 | 0.800858 | 0.199142 |
| WP_007235241.1 | 0.99521 | 0.00479 | 0.99521 | 0.00479 | 0.982992 | 0.017008 |
| WP_007235034.1 | 0.99836 | 0.00164 | 0.99836 | 0.00164 | 0.986264 | 0.013736 |
| WP_007233471.1 | 0.999811 | 0.000189 | 0.999811 | 0.000189 | 0.993252 | 0.006748 |
| WP_007230850.1 | 0.9995 | 0.0005 | 0.9995 | 0.0005 | 0.997482 | 0.002518 |
| WP_007224478.1 | 0.414455 | 0.585545 | 0.414455 | 0.585545 | 0.980164 | 0.019836 |
| WP_007223999.1 | 0.999846 | 0.000154 | 0.999846 | 0.000154 | 0.999837 | 0.000163 |
| WP_156788299.1 | 0.999998 | 1.59E-06 | 0.999998 | 1.59E-06 | 0.99973 | 0.00027 |
| WP_157361215.1 | 0.999568 | 0.000432 | 0.999568 | 0.000432 | 0.999735 | 0.000265 |
| WP_156788350.1 | 0.995342 | 0.004658 | 0.995342 | 0.004658 | 0.892657 | 0.107343 |
| WP_007234293.1 | 0.997145 | 0.002855 | 0.997145 | 0.002855 | 0.97641 | 0.02359 |
| WP_007235113.1 | 0.990324 | 0.009676 | 0.990324 | 0.009676 | 0.985145 | 0.014855 |
| WP_009773280.1 | 0.998295 | 0.001705 | 0.998295 | 0.001705 | 0.997679 | 0.002321 |
| WP_007236262.1 | 0.988299 | 0.011701 | 0.988299 | 0.011701 | 0.947635 | 0.052365 |
| WP_007224080.1 | 0.999993 | 7.25E-06 | 0.999993 | 7.25E-06 | 0.999796 | 0.000204 |
| WP_007236375.1 | 0.885355 | 0.114645 | 0.885355 | 0.114645 | 0.965316 | 0.034684 |
| WP_007226588.1 | 0.96885 | 0.03115 | 0.96885 | 0.03115 | 0.904633 | 0.095367 |

##

**3 Supplementary Figures**

**3.1 ADTree Construction**

**Figure S1 –** The decision tree constructed using the ADTree algorithm, where the tree contains prediction nodes (rectangles) with associated weights representing their contributions to the cumulative prediction score and decision nodes (ellipses) with conditions. Note that, unlike traditional decision trees, the same node can be present several times in the tree to indicate different roles in different places along the tree.

**
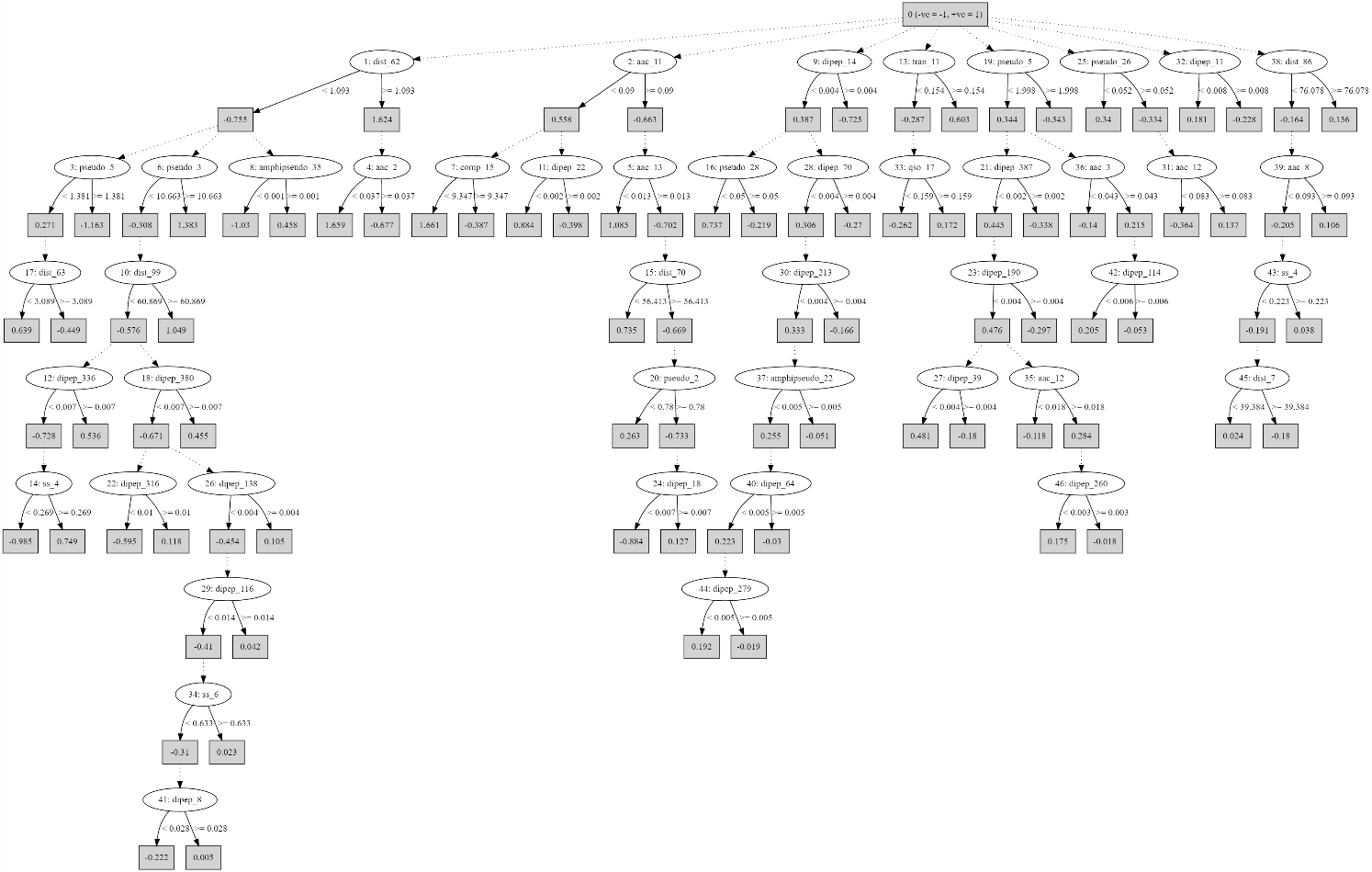
**

**3.2 Confusion matrices**

**Figure S2 –** Confusion matrices of the machine learning models built with the reduced feature sets. In the figure caption, for example, 'F-M', where F indicates the reduced feature set, and M indicates the machine learning model.

| **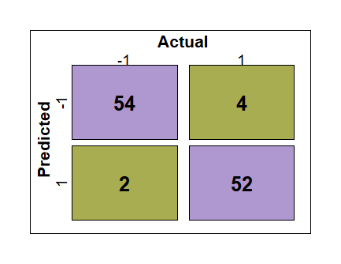**   1. **ADTree-DT** | **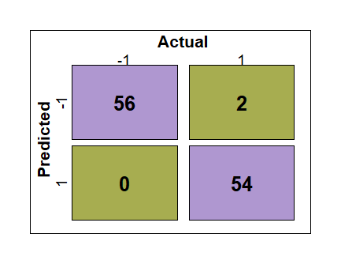**   1. **ADTree-LR** | **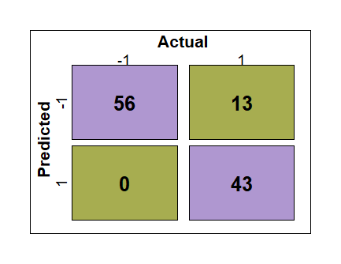**   1. **ADTree-KNN** | **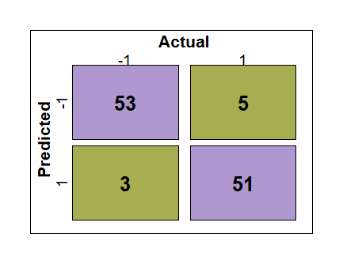**   1. **ADTree-GNB** |
| --- | --- | --- | --- |
| **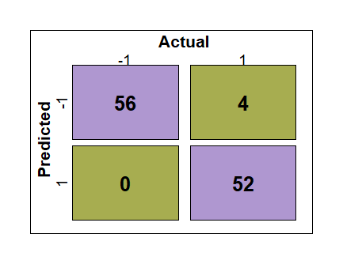**   1. **GA-RF** | **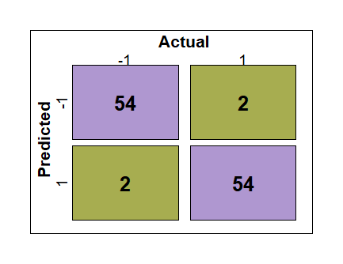**   1. **GA-SVM** | **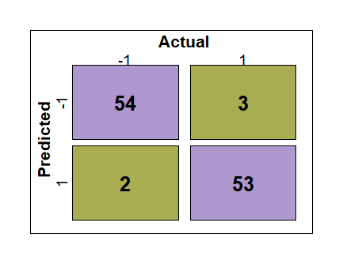**   1. **GA-DT** | **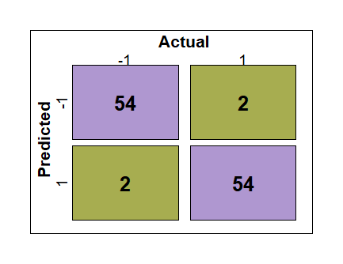**   1. **GA-LR** |
| **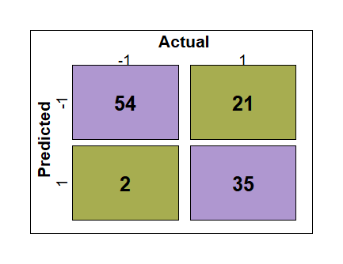**   1. **GA-KNN** | **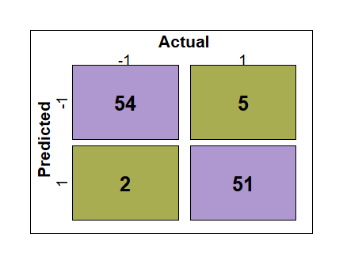**   1. **GA-GNB** | **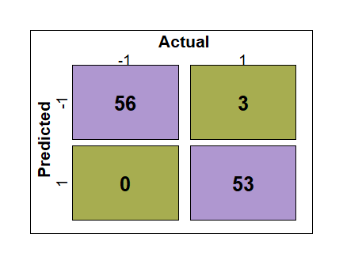**   1. **Linear SVC-RF** | **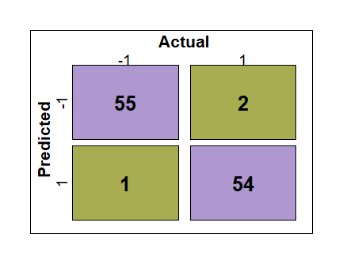**   1. **Linear SVC-SVM** |
| **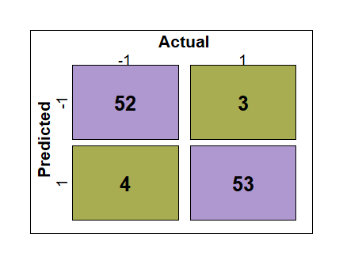**   1. **Linear SVC-DT** | **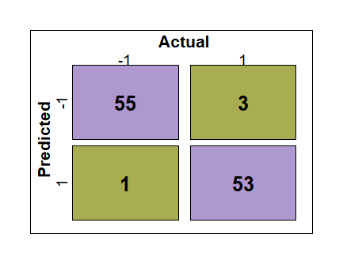**   1. **Linear SVC-LR** | **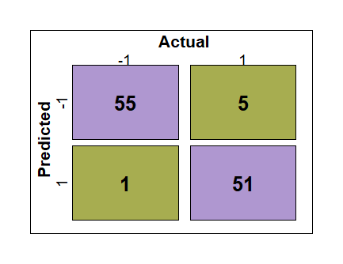**   1. **Linear SVC-KNN** | **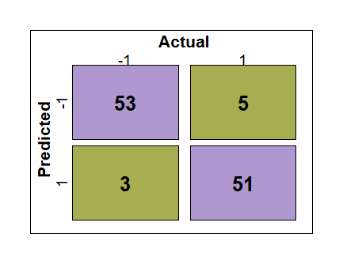**   1. **Linear SVC-GNB** |

**Legend:**

ADTree: Alternative decision tree

GA: Genetic algorithm

Linear SVC : Linear support vector classifier

RF: Random forest

SVM: Support vector machine

DT: Decision tree

LR: Logistic regression

KNN: *k*-nearest neighbors

GNB: Gaussian naïve Bayes
